# The *Callerya* Group redefined and Tribe Wisterieae (Fabaceae) emended based on morphology and data from nuclear and chloroplast DNA sequences

**DOI:** 10.3897/phytokeys.125.34877

**Published:** 2019-06-26

**Authors:** James A. Compton, Brian D. Schrire, Kálmán Könyves3, Félix Forest, Panagiota Malakasi, Sawai Mattapha, Yotsawate Sirichamorn

**Affiliations:** 1 Spilsbury Farm, Tisbury, SP3 6RU, UK Unaffiliated Tisbury United Kingdom; 2 Comparative Plant and Fungal Biology Department, Royal Botanic Gardens, Kew, Richmond, Surrey, TW9 3AE, UK Royal Botanic Gardens Richmond United Kingdom; 3 Herbarium, Royal Horticultural Society Garden, Wisley, Woking, Surrey, GU23 6QB, UK Royal Horticultural Society Garden Wisley United Kingdom; 4 Udon Thani Rajabhat University, Department of Biology, Faculty of Science, Udon Thani 41000, Thailand Udon Thani Rajabhat University Udon Thani Thailand; 5 Silpakorn University, Department of Biology, Faculty of Science, Sanam Chandra Palace campus, Nakhon Pathom 73000, Thailand Silpakorn University Nakhon Pathom Thailand

**Keywords:** Tribe Wisterieae emended, Leguminosae, new genera, *
Austrocallerya
*, *
Kanburia
*, *
Nanhaia
*, *
Serawaia
*, *
Sigmoidala
*, *Wisteriopsis*, molecular phylogeny, morphological key

## Abstract

The Tribe Wisterieae (Zhu 1994), founded on the single genus *Wisteria*, is emended and recircumscribed based on morphology and data from nuclear ITS and *ndhJ-trnF*, *matK* and *rbcL* chloroplast DNA sequences. This newly enlarged tribe comprises 36 species and 9 infraspecific taxa within 13 described genera. Six genera are new, two are reinstated and five were previously placed in Tribe Millettieae. The genus *Adinobotrys* is also reinstated comprising two species including the new combination *A.vastus*. Other reinstated genera include *Whitfordiodendron*, with four species, and *Padbruggea*, with three species, including the reinstatement of *P.filipes* and the new combination P.filipesvar.tomentosa. The existing genera *Afgekia*, *Callerya*, *Endosamara* (with the new combination E.racemosavar.pallida), *Sarcodum* and *Wisteria*, with the new combinations W.frutescenssubsp.macrostachya are evaluated. The new genera comprise three Australasian species in *Austrocallerya*: *A.australis*, *A.megasperma* and *A.pilipes*; *Wisteriopsis* with five species from east Asia has six new combinations: *W.japonica, W.kiangsiensis, W.championii, W.eurybotrya*, *W.reticulata* and W.reticulatavar.stenophylla. Two species comprise the new Thai genus *Kanburia*: *K.tenasserimensis* and *K.chlorantha*. *Nanhaia* comprises the two species: *N.fordii* and *N.speciosa* and the monotypic genera *Sigmoidala* and *Serawaia* are based respectively on the species *S.kityana* and *S.strobilifera*. Lectotypes are designated for the names *Adinobotrysfilipes*, *A.myrianthus*, *Millettiabonatiana, Millettiabracteosa, Millettiachampionii, Millettiacinerea, Millettiadielsiana, Millettiakityana, M.maingayi, Millettianitida, Millettiaoocarpa, Millettiapurpurea*, *M.reticulata*, M.reticulatavar.stenophylla, *Padbruggeadasyphylla*, *Pterocarpusaustralis*, *Robiniaracemosa*, *Whitfordiodendronscandens*, *W.sumatranum* and *Wisteriapallida*. A neotype is designated for the name *Millettialeiogyna*.

## Introduction

The Tribe Millettieae was first described by Miquel (1855: 137), based on the type genus *Millettia* Wight & Arn. (Wight and Arnott 1834: 263). This genus of six species was characterised largely by the pods of the southern Indian type species *M.rubiginosa* Wight & Arn. Miquel emphasised the compressed nature of the pods as a significant distinguishing character and his tribal description very loosely defined the new Tribe Millettieae, which included eight genera: *Brachypterum* (Wight & Arn.) Benth., *Derris* Lour., *Pongamia* Adans., *Padbruggea* Miq., *Aganope* Miq., *Millettia*, *Otosema* Benth. and *Mundulea* Benth. (Miquel 1855: 137).

Miquel (1855: 137) stated:

*stamens monadelphous or diadelphous; calyx campanulate, more or less bilabiate; seed pod indehiscent, woody or leathery, sometimes opening tardily; leaves opposite and pinnate; flowers in racemes*

*Millettia* was, furthermore, distinguished from the genera *Pongamia* Adans. and *Dalbergia* L.f. by the legume being compressed around the seeds and by the fruit’s thick woody texture (Wight and Arnott 1834: 263). Dunn (1912a) in his revision of *Millettia* placed the genus in Tribe Galegeae (Bronn) Torr. & Gray subtribe Tephrosiinae Benth. (as Subtribe “Tephrosieae”). Geesink (1984: 3) described Tribe Millettieae with the characters:

*Inflorescence of panicles, pseudopanicles or derived pseudopanicles; wing petals adherent to the keel; keel petals usually valvately connate; pod dehiscent or indehiscent; seed chamber mostly absent; seeds 1 or few; and without any uniquely defining character”.*Geesink (1984: 4) admitted that “*the contents of this chapter [Delimitations of Millettieae and related tribes] will be disappointing for those who expect a final answer to the questions suggested by the title*”.

Geesink’s major revision of this alliance, which he much enlarged to comprise 43 genera within Tribe Millettieae, was clearly polyphyletic with an assemblage of taxa having a range of unifying as well as contradicting characters. Although far from definitive, this revision was a major step forward and did lay a sound basis for subsequent research in the tribe. Geesink’s (1984) generic treatment included a set of genera in the “*Callerya* Group” (Hu et al. 2002; Hu and Chang 2003): *Wisteria* Nutt. (1818), *Callerya* Endl. (1843), *Afgekia*Craib (1927) and *Endosamara*Geesink (1984). Subsequently, *Sarcodum* Lour. (1790) was also placed in this group (Schrire 2005; Clark 2008) (see Table 1). The genus *Antheroporum* Gagnep., placed tentatively in this grouping by Schrire (2005), has subsequently been shown to belong within the core-Millettieae (LPWG 2016; Mattapha 2017: 53).

**Table 1.** Published treatments of species in the *Callerya* group based on their assignment to genus, from 1984 to the present. Those taxa highlighted in bold represent Chinese species of *Calleryas.l.* for which we were unable to see material. All species epithets are transferable across genera for comparison purposes.

Genus	Geesink (1984) Millettieae	Zhu (1994) Wisterieae	Schot (1994) Millettieae	Lôc (1996) Millettieae	Wei et al. (2010) Millettieae	Sirichamorn et al. (2016) Millettieae	Compton and Lane (2019) Wisterieae
* Adinobotrys *	–	–	–	–	–	–	* A. atropurpureus *
	–	–	–	–	–	–	* A. vastus *
	–	–	–	–	–	–	–
* Afgekia *	* A. filipes *	–	–	* A. filipes *	* A. filipes *	–	–
	* A. mahidoliae *	–	–	* A. mahidoliae *	–	–	* A. mahidoliae *
	* A. sericea *	–	–	* A. sericea *	–	–	* A. sericea *
	–	–	–	–	–	–	–
* Austrocallerya *	–	–	–	–	–	–	* A. australis *
	–	–	–	–	–	–	* A. megasperma *
	–	–	–	–	–	–	* A. pilipes *
	–	–	–	–	–	–	–
* Callerya *	* C. atropurpurea *	–	* C. atropurpurea *	* C. atropurpurea *	–	* C. atropurpurea *	–
	–	–	–	* C. bonatiana *	* C. bonatiana *	–	* C. bonatiana *
	* C. australis *	–	* C. australis *	–	–	–	–
	–	–	–	* C. champoinii *	* C. champoinii *	–	–
	–	–	* C. cinerea *	* C. cinerea *	* C. cinerea *	* C. cinerea *	* C. cinerea *
	–	–	–	–	*** C. congestiflora ***	–	–
	–	–	* C. cochinchinensis *	* C. cochinchinensis *	–	* C. cochinchinensis *	* C. cochinchinensis *
	–	–	–	–	–	* C. chlorantha *	–
	* C. dasyphylla *	–	* C. dasyphylla *	* C. dasyphylla *	–	* C. dasyphylla *	–
	–	–	–	–	* C. dielsiana *	–	* C. dielsiana *
	–	–	–	–	*** C. dorwardii ***	–	–
	–	–	* C. eriantha *	–	–	* C. eriantha *	–
	–	–	* C. eurybotrya *	* C. eurybotrya *	* C. eurybotrya *	* C. eurybotrya *	–
	* C. fordii *	–	* C. fordii *	* C. fordii *	* C. fordii *	–	–
	–	–	–	–	*** C. gentiliana ***	–	–
	–	–	–	–	* C. kiangsiensis *	–	–
	–	–	* C. kityana *	–	–	* C. kityana *	–
	–	–	–	–	*** C. longipedunculata ***	–	–
	–	–	* C. megasperma *	–	–	–	–
	–	–	* C. nieuwenhuisii *	–	–	–	–
	* C. nitida *	–	* C. nitida *	–	* C. nitida *	–	* C. nitida *
	–	–	–	–	* C. oosperma *	–	–
	–	–	* C. pilipes *	–	–	–	–
	* C. reticulata *	–	* C. reticulata *	* C. reticulata *	* C. reticulata *	–	–
	–	–	–	–	*** C. sericosema ***	–	–
	–	–	–	–	*** C. sphaerosperma ***	–	–
	* C. scandens *	–	* C. scandens *	–	–	–	–
* Callerya *	* C. speciosa *	–	* C. speciosa *	* C. speciosa *	* C. speciosa *	–	–
	–	–	* C. strobilifera *	–	–	–	–
	–	–	* C. sumatrana *	–	–	–	–
	–	–	–	–	*** C. tsui ***	–	–
	–	–	* C. vasta *	–	–	–	–
	–	–	–	–	–	–	–
* Endosamara *	* E. racemosa *	–	–	* E. racemosa *	–	–	* E. racemosa *
	–	–	–	–	–	–	–
* Kanburia *	–	–	–	–	–	* C. tenasserimensis *	* K. tenasserimensis *
	–	–	–	–	–	* C. chlorantha *	* K. chlorantha *
* Millettia *	* M. japonica *	* M. reticulata *	–	–	–	–	–
	–	–	–	–	–	–	–
* Nanhaia *	–	–	–	–	–	–	* N. fordii *
	–	–	–	–	–	–	* N. speciosa *
	–	–	–	–	–	–	–
* Padbruggea *	–	–	–	–	–	–	* P. filipes *
	–	–	–	–	–	–	* P. dasyphylla *
	–	–	–	–	–	–	* P. maingayi *
	–	–	–	–	–	–	–
* Sarcodum *	* S. bicolor *	–	–	–	–	–	* S. bicolor *
	* S. scandens *	–	–	* S. scandens *	* S. scandens *	–	* S. scandens *
	–	–	–	–	–	–	* S. solomonensis *
	–	–	–	–	–	–	–
* Serawaia *	–	–	–	–	–	–	* S. strobilifera *
	–	–	–	–	–	–	–
* Sigmoidala *	–	–	–	–	–	–	* S. kityana *
* Whitfordiodendron *	–	–	–	–	–	–	–
	–	–	–	–	–	–	* W. erianthum *
	–	–	–	–	–	–	* W. nieuwenhuisii *
	–	–	–	–	–	–	* W. scandens *
	–	–	–	–	–	–	* W. sumatrana *
	–	–	–	–	–	–	–
* Wisteria *	* W. brachybotrys *	* W. brachybotrys *	–	–	* W. brachybotrys *	–	* W. brachybotrys *
	* W. floribunda *	* W. floribunda *	–	–	–	–	* W. floribunda *
	* W. frutescens *	* W. frutescens *	–	–	–	–	* W. frutescens *
	* W. sinensis *	* W. sinensis *	–	–	* W. sinensis *	–	* W. sinensis *
	–	–	–	–	–	–	–
* Wisteriopsis *	–	–	–	–	–	–	* W. championii *
	–	–	–	–	–	–	* W. eurybotrya *
	–	–	–	–	–	–	* W. japonia *
	–	–	–	–	–	–	* W. kiangsiensis *
	–	–	–	–	–	–	* W. reticulata *

Over the past 30 years a large number of DNA-based phylogenies have analysed many taxa from Tribe Millettieae (Palmer et al. 1987; Lavin et al. 1990; Doyle et al. 1997; Lavin et al. 1998; Doyle et al. 2000; Kajita et al. 2001; Hu et al. 2000; Hu et al. 2002; Hu and Chang 2003; Wojciechowski et al. 2004; Schrire 2005; Schrire et al. 2009; Wink 2013; Li et al. 2014; de Quieroz et al. 2015). Analysis of data from the phytochrome gene family PHY (Lavin et al. 1998) has shown that a core-Millettieae group is monophyletic and may be defined by the presence of pseudoracemes and pseudopanicles. Moreover, while the millettioid-phaseoloid alliance as a whole falls within the large non-protein amino acid accumulating (NPAAA) clade (Wojciechowski et al. 2004; Cardoso et al. 2012; 2013; Wink 2013; Wojciechowski 2013; De Quieroz et al. 2015), Lavin et al. (1998) showed that the core-Millettieae group are diagnosed by a loss of the ability to accumulate the non-protein amino acid canavanine. In these studies the *Callerya* group does not belong with the Millettioid group but rather is accommodated in the Hologalegina clade (Wojciechowski et al. 2000; Lavin et al. 2005). Lavin et al. (1998) also revealed that *Afgekia*, *Callerya*, *Endosamara* and *Wisteria*, i.e. a significant part of the *Callerya* group, did accumulate canavanine rather than alkaloids in their seeds and that they all possessed either true panicles or true racemes. Furthermore, Lavin et al. (1998) postulated that owing to the presence of true racemes, *Sarcodum* was also likely to accumulate canavanine and would therefore not be part of the core-Millettioid group. The morphological distinction between true and pseudoracemes is that in “true” racemes the flowers are inserted singly on the rachis (the unit comprising a flower, pedicel and bract). Pseudoracemes (Lackey 1981) on the other hand, consist of more than one flower inserted at a node on the rachis (the unit comprising two or more flowers, pedicels and bracts all subtended by a secondary bract representing branch reduction). Racemes and pseudoracemes are further compounded into panicles and pseudopanicles.

The *Callerya* group occurs in a more inclusive subset of taxa that all lack one copy of chloroplast DNA, the Inverted Repeat Lacking Clade or IRLC (Palmer et al. 1987; Lavin et al. 1990; Liston 1995; Doyle et al. 1997; Wojciechowski et al. 2000). The loss of a prominent inverted repeat structure in cpDNA in legumes had previously been observed in the genera *Vicia* (Koller and Delius 1980) and *Pisum* (Palmer and Thompson 1981). The genome of *Wisteria* was also discovered to have deleted one half of the inverted repeat amounting to 25 kb of DNA (Palmer et al. 1987). It was apparent that whereas the other legume genera were rearranged genetically as a result of the loss of the inverted repeat, both *Wisteriafloribunda* and *Medicagosativa* remained otherwise unrearranged (Palmer et al. 1987). The IRLC is sister to Tribes Loteae, Sesbanieae and Robinieae (Lewis et al. 2005), which retain the inverted repeat (Lavin et al. 2005; Cardoso et al. 2012; Cardoso et al. 2013; LPWG 2013, 2017). Additional evidence from chloroplast *rbcL* sequence data has also revealed that the Millettieae lie outside the IRLC (Lavin et al. 1990; Doyle et al. 1997; Kajita et al. 2001; Hu and Chang 2003), while the *Callerya* group all fall within the IRLC. These data refute the previously made assumptions that the group belongs with the Millettieae. Lavin et al. (1990) noted that although *Wisteria* and *Millettiajaponica* both showed hypogeal seed germination and a lianescent habit, which are characteristic of many Millettieae genera, these species differed from the Millettieae in their wholly temperate distribution, the lack of the inverted repeat and both had a base chromosome number of x = 8 as opposed to x = 11 or 12. The analyses of Hu and Chang (2003), based on plastid *rbcL* sequence data, confirmed that *Afgekiasericea*, *Calleryavasta*, *Endosamararacemosa*, *Millettiajaponica* and two *Wisteria* species all belonged within the large IRLC. Their results, however, were based on comparatively limited taxon sampling of taxa within the *Callerya* group.

Wink (2013) examined 1276 species of Leguminosae for the distribution of secondary metabolites mapped against phylogenetic trees generated by combined sequence data from cpDNA *rbcL*, *matK* and nrDNA ITS. In the study, it was shown that *Wisteria* and *Callerya* nested within the IRLC and that they possessed isoflavones in common with most but not all other taxa within the IRLC.

A unique marker further distinguishes the *Callerya* group, adding weight to the distinctiveness of this assemblage of genera. Jansen et al. (2008) undertook a comprehensive survey for the retention or loss of two chloroplast introns among 301 legume species representing three subfamilies and 198 genera. Their survey of the presence or absence of the *rps*12 intron revealed that along with 49 of the millettioid-phaseoloids sampled from outside the IRLC, *Afgekiafilipes, A.sericea, Calleryaatropurpurea, C.australis, C.megasperma, C.pilipes, Endosamararacemosa, Millettia* (sic) *japonica, Wisteriabrachybotrys, W.floribunda, W.frutescens, W.macrostachya* and *W.sinensis* - each from inside the IRLC - all retained the intron. Of the 77 other taxa sampled from within the IRLC all - without exception - had lost the intron. Significantly, therefore, all genera within the IRLC surveyed for the presence or absence of the *rps*12 intron showed it to be lacking, except for the *Callerya* group, marking out the latter as unique within the IRLC (Jansen et al. 2008). Seven species of *Glycyrrhiza* surveyed by Jansen et al. (2008), whose position in recent phylogenies (Doyle et al. 2000; Lewis et al. 2005; LPWG 2013, 2017; Li et al. 2014), was placed sister to the *Callerya* group within the IRLC, all lacked the *rps*12 intron. *Glycyrrhiza*, which is represented in our analyses, has therefore not been included as part of the *Callerya* group.

The *Callerya* group is thus uniquely diagnosed by a combination of lacking the 25 kb. inverted repeat of cpDNA and possessing the cpDNA *rps*12 intron. Representatives of *Afgekia*, *Callerya* and *Wisteria* from this subgroup of taxa have also been found to group together according to data from sequences of nuclear DNA ITS spacer regions (Hu et al. 2002; Li et al. 2014).

Zhu (1994) defined her new Tribe Wisterieae comparing only pollen from four species of *Wisteria* to the millettioid-phaseoloid genera *Craspedolobium* Harms, *Derris*, *Millettia*, *Pongamia* and *Tephrosia* Pers. The genus *Pongamia* Adans. is now considered synonymous with *Millettia* Wight & Arn. (Schrire 2005: 383). The pollen grains of the four *Wisteria* species exhibited much broader polar regions (apocolpia) and a distinctive reticulate pollen surface compared to the other taxa examined. Zhu (1994) also made comparisons of *Wisteria* and other millettioid-phaseoloid genera using data from phytochemical and embryological analyses as well as noting the chromosome count of 2n =16 in *Wisteria* compared to those of other genera whose members frequently have 2n = 22 or 2n = 24. It is notable that within the *Callerya* group, two species of *Afgekia*; *A.sericea* and *A.mahidoliae* also have chromosome counts of 2n = 16 (Prathepha 1994; Prathepha and Baimai 2003). Zhu’s (1994) concept of Tribe Wisterieae thus was based solely on four samples of *Wisteria* and one accession of *Millettia* [*Callerya*] *reticulata* (Table 1).

One taxon recently recognised as belonging in the *Callerya* group was included under the names *Wisteriajaponica* or *Millettiajaponica* (Doyle et al. 1997; Doyle et al. 2000; Kajita et al. 2001; Hu and Chang 2003). The inclusion of this taxon in *Wisteria* was originally based on the deciduous leaves, twining habit, pendulous inflorescences and flowers where the wing petals are free from the keel (Siebold and Zuccarini 1839; Bailey and Bailey 1949; Geesink 1984; Iwatsuki et al. 2001; Compton and Thijsse 2013; Compton 2015c). Its summer flowering habit, paniculate inflorescences and absence of callosities on the standard petals have also been used to segregate it from *Wisteria* (Gray 1858; Dunn 1912a; Schot 1994; Valder 1995).

All genera currently comprising the *Callerya* group (Table 1): *Afgekia, Callerya, Endosamara, Wisteria* [incl. *Millettiajaponica*)] and *Sarcodum* possess bracts enclosing the apical floral buds prior to anthesis and all bear either true racemes or true panicles. All are lianas with the exception of two tree species *Calleryaatropurpurea* (Wall.) Schot and *C.vasta* (Kosterm.) Schot.

The genus *Sarcodum* was not included in the analysis of Jansen et al. (2008) and has not been sampled for DNA analysis prior to this paper but the generic morphological characters (Table 4) place it firmly within the *Callerya* group.

The genus *Callerya* Endl., the largest genus within the group with 33 species (Table 1) has subsequently been found to be polyphyletic (Li et al. 2014). Without a comprehensive morphological study of the genus and its near relations and in the absence of additional DNA evidence, it is fair to state that the genus has been something of a catch-all for taxa that bear some morphological affinities with each other (Schot 1994; Lôc 1996). The purpose of this paper therefore is to test generic boundaries within the *Callerya* group by reassessing morphological characters and by a comprehensive molecular sequencing investigation of representative species of all taxa within the group using both nuclear and chloroplast genes.

## Materials and methods

### Molecular preparation and sequencing

Taxon sampling included those taxa in the DNA based phylogenies of Doyle et al. (1997), Hu et al. (2002), Hu and Chang (2003), Jansen et al. (2008) and Li et al. (2014 – but not including several species they recognised in the *Calleryacinerea* complex). Three chloroplast regions were included in the study. Two protein coding genes: *matK* and *rbcL*, and the intergenic spacer *ndhJ*-*trnF.* One nuclear gene region was also included in the study ITS1, 5.8S and ITS2. Fresh DNA was extracted from the previously unsampled *Sarcodumscandens* (Tables 1, 2). For the ITS dataset, 12 additional sequences representing the *Callerya* group and 26 outgroup sequences were included from GenBank; the *matK* dataset comprised an additional five *Callerya* group sequences and 17 outgroup sequences from GenBank and the *rbcL* dataset, a further two ingroup and 12 outgroup sequences from GenBank (Table 2). *Millettiajaponica* has also been confused with *Wisteriafloribunda* in DNA sampling (see GenBank KT119544) and as a result of this, we have chosen to include three different verified samples in this study in order to ascertain its placement within the *Callerya* group.

**Table 2.** Vouchers of taxa used in the phylogenetic analyses. Included are all the *Callerya* group taxa as well as all outgroup taxa used (marked x in the last column). The W numbers represent taxa sampled in this analysis for one or more of three plastid genes (*rbcL*, *ndhJ-TabE* and *matK*) and/or the ITS nuclear spacer regions and G numbers are additional sequences of both ingroup and outgroup taxa downloaded from GenBank.

DNA Extract	Taxon	ITS + GenBank	*ndhJ-TabE* + GenBank	*matK* + GenBank	*rbcL* + GenBank	Vouchers	Outgroup
**G7**	* Abrus precatorius *	AF467015.1				*Hu* 1136 (DAV)	x
**G52**	* Abrus precatorius *			JN407123.2		*Shawpc* 1046K	x
**G74**	* Abrus precatorius *				U74224.1	no voucher data in GenBank	x
**G44**	* Adinobotrys atropurpureus *			AF142734.1		* Callerya atropurpurea *	
**G9**	* Adinobotrys atropurpureus *	AF467015.1				*Liston* 876 (OSC) as *Calleryaatropurpurea*	
**W061**	* Adinobotrys atropurpureus *	MK953946	MK953996	MK965686	MK954049	*S.Mattapha s.n..* Songkhla University, Songkhla Prov. Cult. Thailand (BKF) as *Calleryaatropurpurea*	
**G66**	* Adinobotrys vastus *				AY308806.1	* Callerya vasta *	
**W069**	* Afgekia mahidoliae *	MK953947	MK953997	MK965687	MK954050	*Y.Sirichamorn s.n..* Kanchanaburi, Thailand (BKF)	
**W073**	* Afgekia mahidoliae *		MK953998	MK965688	MK954051	*Y.Sirichamorn* Cult. Suan Luang Rama IX Park, Bangkok, Thailand (BKF)	
**G41**	* Afgekia sericea *	KF294863				*John Mood* 85s47	
**W071**	* Afgekia sericea *		MK953999	MK965689	MK954052	*Y.Sirichamorn* Cult. Suan Luang Rama IX Park, Bangkok, Thailand (BKF)	
**W096**	* Afgekia sericea *		MK954000	MK965690	MK954053	*S.Mattapha* 1158 (BKF)	
**G75**	* Alhagi graecorum *				KX298978	KX298978	x
**G76**	* Alhagi graecorum *	AB854475				TARI 62837	x
**G77**	* Alhagi graecorum *			AB854561		TARI 62837	x
**G73**	* Alhagi maurorum *			JF501101		no voucher data in GenBank	x
**G74**	* Alhagi maurorum *	AB854476				Tabiat Modares University Herbarium 2008-1	x
**G1**	* Astragalus eremiticus *	AF121736.1				no voucher data in GenBank	x
**G22**	* Astragalus mongholicus *	EF685969.1				no voucher data in GenBank	x
**G50**	* Astragalus mongholicus *			EF685993.1		no voucher data in GenBank	x
**G67**	* Astragalus mongholicus *				EF685979.1	no voucher data in GenBank	x
**G10**	* Austrocallerya australis *	AF467024.1				*Beesely* 1053 as *Calleryaaustralis*	
**W087**	* Austrocallerya australis *	MK953949	MK954001	MK965691	MK954054	*R.Johnstone, G.Errington & K.Kupsch* 3251, 4 Feb. 2013. Limpinwood valley Rd. NSW., Australia (K) as *Calleryaaustralis*	
**W068**	* Austrocallerya megasperma *	MK953950	MK954002	MK965692	MK954055	E19697220A Cult. RBG Edinburgh voucher: BROWP1121 (E) as *Calleryamegasperma*	
**W084**	* Austrocallerya megasperma *	MK953951	MK954003	MK965693	MK954056	*J.A.Elsol* 121, 11 May 1977. Moreton Distr. Kinder Park, Qeensland, Australia (K) as *Calleryamegasperma*	
**W092**	* Austrocallerya pilipes *	MK953952				*B.Gray 05144*, 25 Oct. 1989. Cape York, Riflemead, Lerra Logging Area, Queensland, Australia (K) as *Calleryapilipes*	
**W6**	* Austrocallerya pilipes *	MK953953				*B.Gray* 20267v, Australia, Cape York, Harvey Creek, 23 October 1989 (K) as *Calleryapilipes*	
**W115**	* Austrosteenisia glabristyla *	MK953954	MK954004	MK965694	MK954057	*D.L.Jones* 1807 KEW DNAbank 45392	x
**W10**	* Callerya bonatiana *	MK953955		MK965695	MK954058	*E.E.Maire* 1603, China, Yunnan, received 28 October 1912 (K)	
**W063**	* Callerya cinerea *	MK953956	MK954005	MK965696	MK954059	*S.Mattapha* 1113. Doi Phu Ka, Nan Province, Thailand (BKF)	
**W080**	* Callerya cinerea *	MK953957	MK954006	MK965697	MK954060	E00287111, *Calleryacinerea*, Gaoligong Shan Exped. 26273, Yunnan, Longyang, 2 June 2005	
**G31**	* Callerya cochinchinensis *	KF294864.1				*Tang Shaoqing* 201152907 (GNU)	
**G56**	* Callerya cochinchinensis *			KF294877.1		*Tang Shaoqing* 201152907 (GNU)	
**W029**	* Callerya dielsiana *	MK953958	MK954007	MK965698	MK954061	*Song Xianghou* 291, August 1982, Lipo County, Guizhou, China (K)	
**W099**	* Callerya nitida *	MK953959	MK954008	MK965699	MK954062	*Shiu Ying Hu* 6239, 31 October 1971. Tiu Keng Ling, Cha Kuo Ling, Hong Kong, China (K)	
**W101**	* Callerya nitida *	MK953960	MK954009	MK965700	MK954063	*Shiu Ying Hu* 11126, Nov. 24 1968. Victoria Island, Wong Nai Chung, Hong Kong, China (K)	
**G33**	* Callerya oosperma *	KF294870.1				*Tang Shaoqing* 201161901.2 (GNU)	
**G15**	* Clitoria ternatea *	AF467038.1				*Hu* 1068 (DAV)	x
**G75**	* Clitoria ternatea *				U74237.1	no voucher data in GenBank	x
**G29**	* Coronilla coronata *	GQ246136.1				no voucher data in GenBank	x
**G54**	* Coronilla coronata *			JQ619970.1		*A.Mayer* 39 (M)	x
**G73**	* Coronilla varia *				U74222.1	no voucher data in GenBank	x
**G46**	* Coursetia axillaris *			AF543854.1		no voucher data in GenBank	x
**G36**	* Coursetia polyphylla *	KT281061.1				no voucher data in GenBank	x
**G23**	* Disynstemon paullinioides *	EU729484.1				no voucher data in GenBank	x
**W075**	* Endosamara racemosa *		MK954010	MK965701	MK954064	*S.Mattapha & K.Chantavongsa* PK21 Phou Khao Khouay Nat. Park, Vientiane Pref. Laos, 31/10/2105 (HNL)	
**W097**	* Endosamara racemosa *	MK953961	MK954011	MK965702	MK954065	*S.Mattapha s.n..* Sakon Nakhon Prov. Thailand (BKF)	
**W107**	* Endosamara racemosa *	MK953962	MK954012	MK965703	MK954066	*S.Mattapha & M.Norsaengsri s.n..* Mae Kam Pong waterfall, Mai On Distr. Chiang Mai Prov. Thailand (QBG)	
**G5**	* Gliricidia brenningii *	AF398809.1				CEH 1009	x
**G6**	* Gliricidia sepium *	AF398816.1				no voucher data in GenBank 01/11/1986	x
**G47**	* Gliricidia sepium *			AF547197.1		no voucher data in GenBank	x
**G71**	* Gliricidia sepium *				KX119294.1	no voucher data in GenBank	x
**G38**	* Glycyrrhiza glabra *	KY645509.1				no voucher data in GenBank	x
**G63**	* Glycyrrhiza glabra *				AB045804.1	no voucher data in GenBank	x
**G48**	* Glycyrrhiza lepidota *			AY386883.1		no voucher data in GenBank	x
**G51**	* Glycyrrhiza pallidiflora *			EF685997.1		no voucher data in GenBank	x
**G68**	* Glycyrrhiza pallidiflora *				EF685983.1	no voucher data in GenBank	x
**G16**	* Glycyrrhiza uralensis *	AF467050.1				*Hu* 1142 (DAV)	x
**W074**	* Kanburia chlorantha *	MK953963	MK954013	MK965704	MK954067	*Y.Sirichamorn* Y2014-15-1, Sai Yok Distr. Kanchanaburi, Thailand (BKF)	
**W066**	* Kanburia tenasserimensis *	MK953964	MK954014	MK965705	MK954068	*Y.Sirichamorn* YS2015-8 , Suan Phueng Distr. Khoa Chon, Ratchaburi, Thailand (BKF)	
**G25**	* Lotus cytisoides *	FJ938329.1				no voucher data in GenBank	x
**W113**	* Lotus uliginosus *		MK954015	MK965706	MK954069	*J.Compton s.n..* 2018, Wiltshire, England (WSY)	x
**G20**	* Millettia grandis *	AY009139.1				no voucher data in GenBank	x
**G64**	* Millettia pulchra *				AB045810.1	no voucher data in GenBank	x
**G65**	* Millettia richardiana *				AF308714.1	no voucher data in GenBank	x
**G60**	* Millettia xylocarpa *			KY241807.1		no voucher data in GenBank	x
**W2**	* Nanhaia fordii *			MK965707	MK954070	*Yinkun Li* 401972 China, Guangxi Prov. 9 October 1958 (K) as *Calleryafordii*	
**W114**	* Nanhaia speciosa *	MK953965	MK954016	MK965708	MK954071	Kew DNAbank 46791 *Shiu Ying Hu* 8420; ITS, KC441034 as *Calleryaspeciosa*	
**G17**	* Ophrestia radicosa *	AF467484.1				no voucher data in GenBank	x
**G26**	* Padbruggea dasyphylla *	ITS GQ246023.1				* Callerya dasyphylla *	
**W103**	* Padbruggea dasyphylla *		MK954017	MK965709	MK954073	*Y.Sirichamorn* YSM2017-4 , Songkhla, Thailand (BKF) as *Calleryadasyphylla*	
**W102**	* Padbruggea filipes *	MK953968	MK954018	MK965710	MK954074	*Y.Sirichamorn* YSM2017-9, Kanchanaburi, Thailand (BKF) *as Afgekiafilipes*	
**W104**	* Padbruggea filipes *	MK953969	MK954019	MK965711	MK954075	BKF5208, Thung Yai Naresuan, Umphang, Tak, Thailand (BKF) as *Afgekiafilipes*	
**W105**	* Padbruggea filipes *	MK953970	MK954020	MK965712	MK954076	*Y.Sirichamorn* YSM2017-1, Chiang Mai, Thailand (BKF) as *Afgekiafilipes*	
**W036**	Padbruggea filipes var. tomentosum	MK953967	MK954021	MK965713	MK954077	*P.J.Cribb et al.* ASBK 230, 28 March 1997. Napo County, Nonghua, Nongli, Guangxi Prov. China (K) as *Afgekiatomentosa*	
**G28**	* Parochetus africanus *	GQ246124.1				no voucher data in GenBank	x
**G55**	* Parochetus africanus *			JQ619993.1		*J.M.Grimshaw* 94204 (K)	x
**G21**	* Parochetus communis *	DQ311987.1				AL4979	x
**G45**	* Parochetus communis *			AF522115.1		no voucher data in GenBank	x
**G3**	* Phylloxylon xyllophylloides *	AF274684.1				no voucher data in GenBank	x
**G19**	* Platycyamus regnellii *	AF467491.1				no voucher data in GenBank	x
**G30**	* Poissonia orbicularis *	HQ283438.1				*Hughes* 2384 FHO	x
**G4**	* Poitea dubia *	AF398803.1				no voucher data in GenBank	x
**G49**	* Poitea glyciphylla *			AY650278.1		no voucher data in GenBank	x
**W108**	* Sarcodum scandens *	MK953971	MK954022	MK965714	MK954078	*Phan Ke Loc & Vu Xuan Quang*P11554, 27 April 2017, Quang Binh Prov. Vietnam (CPNP, IBSC)	
**W112**	* Sarcodum scandens *	MK953972	ndhJ-TabE (MK954023	MK965715	MK954079	*S.Lanorsavanh* 1299, July 2017. Sop Teuang, Xaychamphone Distr. Bolikhamxai Prov. Laos (HNL, FOF)	
**G24**	* Schefflerodendron usambarense *	EU752495.1				no voucher data in GenBank	x
**G59**	* Schefflerodendron usambarense *			KX652187.1		no voucher data in GenBank	x
**W095**	* Schefflerodendron usambarense *	MK953973	MK954024	MK965716	MK954080	*H.Fandey, K.A.Siwa & H.O.Suleiman* TTSA/MSB 48, 28 July 2007. Tanga Reg, Muheza Distr, Kisiwani, Tanzania (K)	x
**G2**	* Securigera varia *	AF218537.1				no voucher data in GenBank	x
**G62**	* Securigera varia *			MG221137.1		JAG 0617	x
**W3**	* Serawaia strobilifera *	MK953966			MK954072	*Lomudin Tadong* 308, Sabah, Ranau distr. 15 May 1995 (K) as *Calleryastrobilifera*	
**G39**	* Sesbania arborea *	JX453663				*W.Wagner et al.* 4912 (F)	x
**G40**	* Sesbania cavanillesii *	JX453671				*T.Gonzalez Guizer*	x
**G43**	* Sesbania herbacea *			HQ730419		no voucher data in GenBank	x
**G76**	* Sesbania herbacea *				KJ773881	no voucher data in GenBank	x
**G42**	* Sesbania punicea *			HQ730418		no voucher data in GenBank	x
**G77**	* Sesbania vesicaria *				KJ773882	no voucher data in GenBank	x
**G61**	* Sigmoidala kityana *			KY241809.1		*Calleryakityana* voucher 1117	
**W064**	* Sigmoidala kityana *	MK953974	MK954025	MK965717	MK954081	*S.Mattapha* 1117, Chalerm Prakiat Distr. Nan Province, Thailand (BKF) as *Calleryakityana*	
**W5**	* Sigmoidala kityana *	MK953975			MK954082	*R.P.Clark* 245,Thailand, Loei Prov. Nong Hin, 15 November 2011 (K) as *Calleryakityana*	
**W106**	* Whitfordiodendron erianthum *	MK953976	MK954026	MK965718	MK954083	*Y.Sirichamorn* YSM2017-3, Songkhla, Thailand (BKF) as *Calleryaeriantha*	
**G12**	* Whitfordiodendron nieuwenhuisii *	AF467029.1				*Ambriansya & Arifiu 293 (L)* as *Calleryanieuwenhuisii*	
**G27**	* Whitfordiodendron nieuwenhuisii *	GQ246025.1				no voucher data in GenBank as *Calleryanieuwenhuisii*	
**W098**	* Whitfordiodendron scandens *	MK953977	MK954027	MK965719	MK954084	*S.M.Hi* 416, 11 March 1984. Palawan, Taytay municip. Philippines (K) *Calleryascandens*	
**W009**	* Wisteria brachybotrys *	MK953978	MK954028	MK965720	MK954085	*Yuri Kurishigi* 581, Ishimi river, Honshu, Japan (GENT)	
**W021**	* Wisteria brachybotrys *	MK953979	MK954029	MK965721	MK954086	*J.Compton* W021. Ushijima, Honshu, Japan, cult. (WSY)	
**W043**	* Wisteria brachybotrys *	MK953980	MK954030	MK965722	MK954087	*J.Compton* W043. Kitakyushu, Kyushu, Japan (RNG)	
**W017**	* Wisteria floribunda *	MK953981	MK954031	MK965723	MK954088	*J.Compton* W017. Ushijima, Honshu, Japan cult. (WSY); f. multijuga	
**W019**	* Wisteria floribunda *	MK953982	MK954032	MK965724	MK954089	*J.Compton* W019. Ushijima, Honshu, Japan cult. (WSY)	
**W020**	* Wisteria floribunda *	MK953983	MK954033	MK965725	MK954090	*J.Compton* W020. Kameido Tenjin shrine, Tokyo, Honshu, Japan cult. (WSY); multijuga	
**W044**	* Wisteria floribunda *	MK953985	MK954034	MK965726	MK954091	*J.Compton* W044. Fukuoka, Kyushu, Japan (RNG)	
**W046**	* Wisteria floribunda *	MK953986	MK954035	MK965727	MK954092	W20051758-A (WSY); ‘Hime’	
**W012**	Wisteria frutescens subsp. frutescens	MK953987	MK954037	MK965728	MK954094	*C.Lane* Cult. (WSY); ‘Amethyst Wave’	
**W037**	Wisteria frutescens subsp. macrostachya	MK954125	MK954038	MK965729	MK954095	*C.Lane* Cult. (WSY); ‘Bayou Two o’Clock’	
**W038**	Wisteria frutescens subsp. macrostachya	MK954126	MK954039	MK965730	MK954096	*C. Lane* Cult. (WSY); ‘Clara Mack’	
**W002**	* Wisteria sinensis *	MK954113	MK954040	MK965731	MK954099	*M Libert* ML 211A , Beijing Botanic Garden seed, Cult. (GENT)	
**W024**	* Wisteria sinensis *	MK954121	MK954041	MK965732	MK954100	*J.Compton* W024 Cult. Koishikawa Bot. Garden (WSY)	
**W025**	* Wisteria sinensis *	MK954122	MK954042	MK965733	MK954101	*J.Compton* Cult. Forbes Place, Chichester, UK (RNG)	
**W110**	* Wisteria sinensis *	MK954156	MK954044	MK965734	MK954102	W2006.0790 Hengshan, Hunan, China (ML234 Ghent B. G.) Cult. (GENT)	
**W8**	* Wisteriopsis championii *				MK954103	*Shiu Ying Hu* 10476, Hong Kong, Fo-tan valley, 20 June 1970 (K) as *Calleryachampionii*	
**G11**	* Wisteriopsis eurybotrya *	AF467027.1				*Tao* 578 KUN as *Calleryaeurybotrya*	
**G32**	* Wisteriopsis eurybotrya *	KF294868.1				*Tang Shaoqing* 201161501 (GNU) as *Calleryaeurybotrya*	
**G57**	* Wisteriopsis eurybotrya *			KF294879.1		*Tang Shaoqing* 201161501 (GNU) as *Calleryaeurybotrya*	
**W032**	* Wisteriopsis japonica *		MK954046	MK965737	MK954104	*M.Furuse* 9745, 22 Oct. 1975. Kyushu Island, Tarumi Ku, Koobe-shi, Japan (K)	
**W045**	* Wisteriopsis japonica *	MK954129	MK954047	MK965738	MK954105	*J.Compton s.n..* Nagasaki, north side of harbour, Kyushu, Japan (RNG)	
**W088**	* Wisteriopsis japonica *		MK954048	MK965739	MK954106	*M.Togashi* 7888, 20 Aug. 1961. Honshu, Minoo, Higashidani Pref. Oosaka, Japan (K) as *Wisteriajaponica*	
**W1**	* Wisteriopsis japonica *	MK954147		MK965740	MK954107	JCRaulston Arboretum, N. Carolina, USA. 980008-17 Cult. Ex Japan. as *Millettiajaponica*	
**G13**	* Wisteriopsis reticulata *	AF467031.1				*Liston* 877 (OSC) as *Calleryareticulata*	
**G34**	* Wisteriopsis reticulata *	KF294872.1				*Tang Shaoqing* 201152906 (GNU) as *Calleryareticulata*	
**G53**	* Wisteriopsis reticulata *			JQ619954.1		*M.F.Wojciechowski* 1278 (ASU) as *Calleryareticulata*	
**G72**	* Wisteriopsis reticulata *				KX527123.1	CPG10050 as *Calleryareticulata*	
**W109**	* Wisteriopsis reticulata *		MK954049	MK965741	MK954108	*J.Compton s.n..*Cult. (WSY) as *Calleryareticulata*	
**G18**	* Xeroderris stuhlmannii *	AF467485.1				no voucher data in GenBank	x

Outgroup taxa (Table 2) for each analysis comprised several accessions that represented taxa from other Tribes within the IRLC e.g. Hedysareae, Galegeae and Trifolieae (Lewis et al. 2005) and several from outside the IRLC, e.g. Robineae, Loteae, Sesbaneae, Millettieae, Abreae, Phaseoleaeand Indigofereae. The genus *Schfflerodendron* Harms was selected as the outgroup upon which to root all trees owing to its position in the LPWG (2017) RAxML Maximum Likelihood analysis. Its position as sister to *Calleryaatropurpurea* – and these two to *Glycyrrhiza* – in turn all sister to the *Callerya* group and the rest of the IRLC suggest that it is the most appropriate candidate for choice as outgroup. Additional outgroup sequences were generated of W095 (see codes to samples, Table 2) *Schefflerodendronusambarense* (Tribe Millettieae), W113 *Lotusuliginosus* (Tribe Loteae) and W115 *Austrosteenisiaglabristyla* (Tribe Millettieae) with the addition of 14 other legume genera from GenBank representing additional Tribes all of which sit outside the IRLC (Table 2).

We generated 49 sequences of the nrDNA ITS spacer region, including one for the outgroup taxon *Schefflerodendronusambarense*. Sequence data was also generated for three plastid markers: 51 *Callerya* group sequences from the *ndhJ-trnF* cpDNA intergenic spacer, 53 sequences from the *matK* gene and 57 sequences from the *rbcL* gene. Sequences of three outgroup taxa (i.e. *Austrosteenisiaglabristyla, Lotusuliginosus* and *Schefflerodendronusambarense*) were also obtained for these three plastid markers (see Table 2). Summary statistics of support levels at critical nodes of all trees generated in this study (Suppl. material 1 Figs S1–S6), derived from Maximum Likelihood (ML) analysis and Bayesian inference (BI) analysis, are shown in Table 3.

**Table 3.** Summary of support values at critical nodes for trees derived from the six phylogenetic analyses (Suppl. material 1: Figs S1–S6). Levels of ML Bootstrap (BS) – and Bayesian Inference (BPP) – support are listed for each of the combined plastid and nuclear, plastid only and ITS analyses, for genera and clades discussed in the text. Rows in bold represent genera. Single accessions are where only one sequence was available for a taxon and comments are included in the table highlighting those conflicting arrangements of taxa between the plastid and ITS analyses. BS and BPP support of 85%/0.95 and higher are considered strong, 65–85%/0.9–0.95 as moderate, and below 65%/0.9 as weak.

Clade/Clades	Taxa	Combined Bootstrap support Maximum Likelihood (ML)	Combined Bayesian Posterior Probability (BPP)	Plastid Bootstrap support Maximum Likelihood (ML)	Plastid Bayesian Posterior Probability (BPP)	ITS Bootstrap support Maximum Likelihood (ML)	ITS Bayesian Posterior Probability (BPP)
IRLC	*Parochetus – Wisteria*	99%	1	92%	1	68%	1
A–E + *Glycyrrhiza* + *Adinobotrys*	*Glycrrhiza (Gly) – Adinobotrys (Adin) – Wisterieae (Wist)*	Grade	Grade *Adin* sister to *Gly* + *Wist* (0.65); *Gly* sister to *Wist* (0.49)	Grade	Clade *Gly* sister to *Adin* (0.42), both sister to *Wist* (0.6)	*Gly* sister to all IRLC (23%); *Adin* sister to IRLC excluding *Wist* (22%)	*Gly* sister to all IRLC (0.63); *Adin* sister to IRLC excluding *Wist* (0.41)
	*** Adinobotrys ***	**100**%	**1**	**100**%	**1**	**100**%	**1**
A–E	*Tribe Wisterieae*	100%	1	100%	1	61%	1
A	*Sarcodum – Sigmoidala*	100%	1	100%	1	59%	0.86
A2 + A3	*Sigmoidala – Endosamara*	98%	1	61%	0.9	*Endosamara* excluded	*Endosamara* included 0.72
**A1**	*** Sarcodum ***	**100**%	**1**	**100**%	**1**	**100**%	**1**
**A2**	*** Endosamara ***	**100**%	**1**	**100**%	**1**	**100**%	**1**
**A3**	*** Sigmoidala ***	**99**%	**0.99**	**99**%	**1**	**100**%	**1**
B–E	*Nanhaia – Wisteria*	75%	0.99	87%	0.91	groups with	0.83
B	*Nanhaia – Wisteriopsis*	100%	1	100%	1	91%	0.99
**B1**	*** Nanhaia ***	**100**%	**1**	**100**%	**1**	**Single accession**	**Single accession**
**B2**	*** Wisteriopsis ***	**91**%	**1**	**97**%	**1**	**100**%	**1**
C–E	*Callerya – Wisteria*	99%	1	91%	1	clade C groups with elements of clades A and B	clade C groups with clade B
C	*Callerya – Afgekia*	87%	0.98	*Serawaia* excluded	*Serawaia & Kanburia* excluded	*Endosamara* included 28%	groups with clade B
**C1**	*** Callerya ***	**64% (100% above *C.bonatiana***	**including *C.bonatiana* 0.95**	**48% (74% above *C.bonatiana***	**including *C.bonatiana* 0.96**	*** C. bonatiana ***	***C.bonatiana* excluded**; groups, with no support, in Clade B
C2 + C3 + C4	*Whitfordiodendron – Afgekia*	72%	0.99	* Serawaia *	*Serawaia & Kanburia* excluded	53%	0.8
**C2**	*** Whitfordiodendron ***	**100**%	**1**	**99**%	**1**	**100**%	**0.99**
**C2/C3**	*** Serawaia ***	**Single accession; groups with C2, with no support (13%)**	**Single accession; groups with C2, with moderate support (0.93)**	**Single accession; unresolved position relative to Clades C, D & E**	**Single accession; no support in Clade C, unresolved position relative to Clades D & E**	**Single accession; groups with C3 65**%	**Single accession; groups with C3 0.72**
**C3**	*** Kanburia ***	**100**%	**1**	**100**%	**1**	**85**%	**0.99**
**C4**	*** Afgekia ***	**100**%	**1**	**100**%	**1**	**100**%	**1**
D + E	*Padbruggea – Wisteria*	98%	1	68%	0.97	90%	1
D	*Padbruggea – Austrocallerya*	99%	1	69%	0.97	97%	1
**D1**	*** Padbruggea ***	**91**%	**1**	**93**%	**1**	**91**%	**1**
**D2**	*** Austrocallerya ***	**98**%	**1**	**94**%	**1**	**93**%	**1**
**E**	*** Wisteria ***	**100**%	**1**	**100**%	**1**	**99**%	**1**
E1	American Clade	100%	1	100%	1	100%	1
E2	Asian Clade	100%	1	100%	1	99%	1

The DNA extraction protocol for all 54 samples (with numbers from W002 to W115) and the seven samples labelled W1, W2, W3, W5, W6, W8 and W10 (Table 2) used a modified CTAB protocol (Doyle and Doyle 1987). DNA extraction from herbarium specimens followed the protocol used by Särkinen et al. (2012) with some minor amendments.

For all accessions labelled W002 to W115 (Table 2), a circa 800 bp part of *matK* was amplified with the previously unpublished primers designed by Ki-Joong Kim: 1RKIM-f – ACCCAGTCCATCTGGAAATCTTGGTTC and 3FKIM-r – CGTACAGTACTTTTGTGTTTACGAG (Dunning and Savolainen 2010). PCR reactions were performed in 25μl volumes containing final concentrations of 1× Bioline Biomix Red, 0.35μM of each primer, 0.2mg/ml BSA (bovine serum albumin), 4% v/v DMSO (dimethyl sulfoxide) and 20ng DNA template. Cycling conditions were 94 °C for 120s, then 35 cycles of 94 °C for 30s, 48 °C for 30s, 72 °C for 60s, and finally 72 °C for 7 minutes.

For all accessions (Table 2) the gene *rbcL* was amplified with primers 1F (Fay et al. 1997) and 1460R (Fay et al. 1998). To amplify degraded and/or low quality DNA two overlapping shorter fragments were amplified with the original primers and internal primers 636F and 724R (Fay et al. 1997). PCR reactions for all primer combinations were performed in 25μl volumes containing final concentrations of 1× Bioline Biomix Red, 0.35μM of each primer, 0.2mg/ml BSA (bovine serum albumin), and 20ng DNA template. Cycling conditions for the reactions using primers 1F and 1460R were 94 °C for 120s, then 30 cycles of 94 °C for 60s, 51 °C for 30 s, 72 °C for 120s, and finally 72 °C for 7 minutes. For the shorter fragments this protocol was modified by decreasing the elongation time to 90s and increasing the number of cycles to 40 for weaker amplicons.

Again for all accessions, the intergenic spacer *ndhJ*-*trnF* was amplified with the primers *ndhJ* and *TabE* using the PCR protocol listed in Shaw et al. (2007). Low quality or degraded DNA necessitated the utilisation of primers that amplified two shorter, overlapping segments of this region. We designed two additional primers internal to the *ndhJ-trnF* spacer in order to overcome this problem: 456F – ATGGGCCGGATTCTATTTGT and 725R – TGATTAGTGGTCTAGATCATCAT. The PCR protocol for the shorter fragments was the same as above, apart from increasing the number of cycles to 40 for weaker amplicons.

For all accessions the nrDNA Internal Transcribed Spacers (ITS1 and ITS2) were amplified with primers ITS4 and ITS5 (White et al. 1990; Baldwin et al. 1995) or with 17SE and 26SE (Sun et al. 1994). The PCR reactions for ITS4 and ITS5 were performed in 25μl volumes containing final concentrations of 1× Bioline Biomix Red, 0.5μM of each primer, 0.2mg/ml BSA (bovine serum albumin), and 10 - 25 ng DNA template. Cycling conditions were 94 °C for 120s, then 30 cycles of 94 °C for 60s, 48 °C for 60 s, 72 °C for 90s, and finally 72 °C for 7 minutes. The PCR reactions for 17SE and 26SE were performed in 25μl volumes containing final concentrations of 1× Bioline Biomix Red, 0.35μM of each primer, 0.2mg/ml BSA (bovine serum albumin), and 20ng DNA template. Cycling conditions were 94 °C for 120s, then 40 cycles of 94 °C for 30s, 63 °C for 60 s, 72 °C for 60s, and finally 72 °C for 7 minutes.

Sequencing of 44 taxa for ITS and 54 taxa for *ndhJ-trnF*, *matK* and *rbcL* were performed at GATC Biotech (www.gatc-biotech.com; Konstanz, Germany).

For the seven accessions labelled W1 to W10 (see Table 2) DNA extractions used a similar protocol to that mentioned above. PCR amplifications were performed using the same primers as already mentioned for nrDNA ITS, cpDNA *matK* and cpDNA *rbcL* with the following different protocol: PCRs were performed in 25 μL volumes, containing 12.5 μL DreamTaq PCR Master Mix (2×) (4 mM MgCl2; Thermo Fisher Scientific, Waltham, MA, USA), 0.5 μL of each primer (100 ng μL−1), and 1 μL DNA template. TBT-PAR [trehalose, bovine serum albumin (BSA) and polysorbate-20 (Tween-20)] was added to reduce the inhibitory effects of polysaccharide and phenolic compounds (Samarakoon et al. 2013).

For the accessions W1 to W10 (Table 2) all amplifications were performed on a 9700 GeneAmp thermocycler (ABI, Warrington, UK). All PCR products were purified with either the QIAquick PCR kit (Qiagen, Hilden, Germany) or the Nucleospin Extract II kit (Machery-Nagel, Düren, Germany), following the manufacturers’ protocols. Cycle sequencing reactions were performed in 5 μL reactions using 0.5 μL BigDye Terminator cycle sequencing chemistry (v3.1; ABI) and the same primers as for PCR. Complementary strands were sequenced on an ABI3730 automated sequencer.

### Phylogenetic procedures and analyses

Sequences of each region were edited and compiled in Geneious (version 8.1.9; Kearse et al. 2012) and aligned with the MUSCLE algorithm (Edgar 2004) implemented in AliView (Larsson 2014). The ends of the alignments were trimmed to the point where all sequences were present and base calls were unambiguous.

Phylogenetic analyses were conducted on the plastid, ITS and combined plastid/ITS matrices using two approaches, Maximum likelihood (ML) and Bayesian inference (BI). For the ML approach, we used the software RAxML (v. 8.2.8; Stamatakis 2014) as implemented on the CIPRES portal (www.phylo.org) with 1,000 rapid bootstrap replicates followed by the search of the best ML tree; the GTRCAT model was used and all the other parameters were set as default settings. The Bayesian Markov Chain Monte Carlo (MCMC) approach was performed using the software MrBayes (version 3.2.6; Ronquist and Huelsenbeck 2003) as implemented on the CIPRES portal. The best-fit DNA substitution model was tested using JModelTest 2 (version 2.1.6; Darriba et al. 2012) as implemented on the CIPRES portal. The General Time Reversible (GTR) model with a proportion of invariable sites and a gamma shape to account for rate heterogeneity among sites (GTR+I+G) was selected for both partitions. The analyses were run twice each for 10 million generations and sampled every 1000^th^ generation. The MCMC sampling was verified using Tracer (Rambaut and Drummond 2009) and was considered adequate when the effective sampling size was higher than 200. A burn-in period of one million generations was applied to each run. The remaining trees from both runs were compiled using the ‘‘allcompat’’ option in MrBayes to produce a maximum credibility tree with Bayesian posterior probabilities (BPP) for each node. In both combined ML and BI analyses, the plastid and ITS partitions were allowed to have partition-specific model parameters. *Schefflerodendronusambarense* was designated as outgroup taxon in all analyses. Support values for nodes of critical taxa in the Discussion are shown in Table 3.

### Morphological study

The morphological key to the species was based on examination of living material in cultivation in UK and USA and in the wild in China, Japan, Laos, Myanmar, Thailand and Vietnam. Herbarium specimens were examined including the collection of all relevant genera in the *Callerya* group at K and BM. Online collections were examined at the Chinese Virtual Herbarium, CVH (http://www.cvh.ac.cn/en); JSTOR Global Plants (https://plants.jstor.org/); Herbarium, Muséum National d’Histoire Naturelle, Paris, MNHN (https://science.mnhn.fr/institution/mnhn/collection/p/item/search/form?lang=en_US); Herbarium Royal Botanic Garden, Edinburgh, RBGE (http://data.rbge.org.uk/search/herbarium/) and Nederlandse Natuurhistorische Collecties, Naturalis (http://bioportal.naturalis.nl/). See Appendix 1 for a full list of all specimens used as the basis for the new generic descriptions. Herbarium acronyms follow Thiers (2019, http://sweetgum.nybg.org/science/ih/). A full list of all Herbaria cited is found in the acknowledgements. A list of the critical characters measured for this study is shown in Tables 4, 5.

**Table 4.** Morphological character comparison across genera in Tribe Wisterieae. Comparison is made of critical distinguishing characters for the 14 genera treated here. Character traits highlighted in bold are considered uniquely grouping (or autapomorphic) within Tribe Wisterieae.

Characters	* Adinobotrys * ^1^	* Endosamara * ^2^	* Sigmoidala * ^3^	* Sarcodum * ^4^	* Wisteriopsis * ^5^	* Nanhaia * ^6^	* Callerya * *s. str.* ^7^	* Serawaia * ^8^	* Kanburia * ^9^	* Whitfordiodendron * ^10^	* Afgekia * ^11^	* Padbruggea * ^12^	* Austrocallerya * ^13^	* Wisteria * ^14^
Habit and leaf persistence	trees, evergreen	liana, evergreen	liana, evergreen	liana, evergreen	liana, deciduous (*W.japonica*) or evergreen	liana, evergreen	liana, evergreen	liana, evergreen	liana, evergreen	liana, evergreen	liana, evergreen	liana, evergreen	liana, evergreen	liana, deciduous
Leaflet number	7–11	9–13	7–9	9–45	5–15	5–17	3–7	5–7	5	3–13	9–17	9–19	5–19	9–15
Stipule length	2–4 mm	5–10 mm	3–6 mm	3–12 mm	2–4 mm	2–4 mm	1–6 mm	5–8 mm	1–4.5 mm	1.5–4 mm	**10–25 mm**	1–8 mm	1.5–6 mm	2–6 mm
Gibbosity presence/absence below stipule	absent	absent	absent	absent	**prominent gibbosities below stipule insertions**	**prominent gibbosities below stipule insertions**	absent	**prominent gibbosities below stipule insertions**	absent	absent	absent	absent	absent	absent
Inflorescence type	panicle	panicle	panicle	**raceme**	panicle	panicle	panicle	**raceme** / panicle with few branches	panicle	panicle	**raceme**	panicle	panicle	**raceme**
Pedicel length	5–6 mm	3–6 mm	3–4 mm	4–12 mm	2–7 mm	4–11 mm	2–8 mm	4–6 mm	2–6 mm	0.5–3 mm	7–20 mm	4–25 mm	3–20 mm	5–50 mm; (5–20 mm *W.frutescens*)
Floral bract length and persistence	2–3 mm; persistent (caducous *A.vastus*)	6–15 mm; caducous	5–6 mm, caducous	6–20 mm; caducous	3–6 mm; persistent	3–11 mm, persistent (caducous *N.fordii*)	1–8 mm; caducous	15–18 mm; persistent	1–4 mm; caducous	3–7 mm; caducous	15–35 mm; caducous	4–20 mm; caducous	2–15 mm; caducous	5–15 mm; caducous
Bracteoles present/absent	present at base of calyx, persistent	**absent**	**absent**	present, apex of pedicel	present, persistent, at tip of pedicel	present, persistent, at tip of pedicel/ base of calyx	present, often caducous placed on the petioles (to base of calyx in *C.nitida*)	present, caducous, near top of pedicel	**absent**	present, placed on the calyx above the base (at base in *W.sumatrana*)	absent	present on upper half of pedicel	present on top of pedicel	present **(absent** in *W.frutescens*)
Flower size - small 0.7–1.4 (1.5) cm; large (1.5) 1.6–3.5 cm	large, (1.4)1.5–2 cm long	small 1.2–1.6 cm long	large, 1.6 -– 2 cm long	small, 0.6–1.3 cm long	small (0.7)1.0–1.5 (1.6) cm long	large (1.5) 1.6–3.2 cm long	large (1.2)1.6–2.8 cm long	large (1.5) 1.6–21 mm, yellow	small, 1–1.4 (1.5) cm long	small, 1– 1.5 cm long (to 2.3 cm long in W.eriantha s.s.)	large, 2–2.5 cm long	small, 1.3–2.5 cm long	small, 1.1–1.6 cm long	large, 1.5–3 cm long
Standard dorsal surface indumentum presence/absence	**glabrous**	**glabrous**	densely pubescent	**glabrous**	**glabrous**	**glabrous**	sericeous	pubescent	sericeous	sericeous	pubescent	pubescent	pubescent	sparsely pubescent (glabrous in *W.frutescens*)
Callosity presence and type at base of standard petal	boss	boss	boss	boss	boss	boss	ridge or boss	boss	ridge	ridge or boss	**papillate with 2 upper corniculate**	ridge/ **papillate** (in *P.filipes*)	**arched**	**papillate** (ridge in *W.frutescens)*
Length of wings in proportion to keel; wing attachment to keel	slightly longer than and adherent to keel	slightly longer than and adherent to keel	**wings sigmoid, reflexing after anthesis**, longer than and adherent to the keel	shorter than or equalling and adherent to keel	more or less equalling and **mostly free** from keel	more or less equalling and adherent to keel	**shorter than** and adherent to keel	more or less equalling **and free from** keel	more or less equalling the keel to slightly longer, adherent to keel	more or less equalling and adherent to keel	more or less equalling and adherent to keel	more or less equalling and adherent to keel	more or less equalling and adherent to keel	more or less equalling keel, **sometimes free from keel**
Keel indumentum presence/absence	glabrous	glabrous	glabrous	glabrous	glabrous	glabrous	glabrous	glabrous	glabrous	**densely sericeous particularly along lower margin**	pubescent	glabrous or densely pubescent along lower margin	glabrous or very sparsely hairy along lower margin	glabrous
Staminal column free or enclosed within keel at anthesis	enclosed	enclosed	enclosed	enclosed	**free**	enclosed	enclosed	enclosed	enclosed	enclosed	enclosed	enclosed	enclosed	enclosed
Style length at anthesis; short 2–4 (5) mm long; long 6–9(10) mm long	5–6 mm	4–5 mm	2–3 mm	3–4 mm	3–4 mm	2–3 mm	6–9 mm	4 - 6 mm	2–3 mm	2–4 mm	1–3 mm	3–4 mm	3–5 mm	3–5 mm
Ovary indumentum presence/ absence	sparsely to densely hairy	**glabrous**	**glabrous**	**glabrous**	**glabrous**	sericeous	sericeous	sparsely hairy	sericeous	sericeous	densely pubescent	sericeous	sericeous	pubescent
Pod shape, surface structure; indumentum	7–20 × 3–6 cm; inflated, obovoid or oblong; glabrous, rugose	8–25 × 1–2 cm; flattened; linear, glabrous, smooth, raised above seeds contracted between them, veins visible?	7–11 × 1–2 cm, flattened, narrowly obovate, glabrous, smooth	3.5–5 × 0.7–1.2 cm; botuliform, linear; glabrous (exocarp fleshy, endocarp thin, forming transverse septae between seeds), not convex around seeds	8–12 × 0.8–3 cm, linear to narrowly ovate, compressed, glabrous, finely corrugated	15–25 × 1–2.5 cm, linear to narrowly obovate, flattened, not or slightly inflated, not convex around seeds, densely hairy, smooth	5–21 × 0.7–4 cm; flattened, linear to narrowly ovate, elliptic or obovate - or - inflated, convex around seeds and contracted between them; tomentose to densely pubescent, smooth	19–30 × 2.5–3.5 cm; narrowly obovate, flat, beaked, shortly hirsute, smooth	5–13 × 1–1.8 cm., compressed, strap-shaped, glabrescent, slightly inflated and convex around seeds, contracted between seeds	4–9.5 × 2–5 cm; inflated, ovoid; rugose to ridged or ruminate, velutinous (sparsely pubescent in *W.nieuwenhuisii*)	6–15 × 3–4 cm; inflated; oblong, obliquely obovate to fusiform, velutinous , smooth to slightly wrinkled	10–25 × 5–11 cm; inflated, obovoid or oblong; coarsely ridged to rugose; tomentose	7–23 × 3–5.2 cm, inflated, fusiform; torulose, finely ridged or striate, velutinous	10–24 × 1.2–3 cm; compressed; oblanceolate; velutinous (*W.frutescens* 8 × 12 cm long; linear- oblanceolate; glabrous)
Fruit endocarp septae type	subseptate	**septate**	subseptate	**septate**	subseptate	subseptate	subseptate	subseptate	subseptate	subseptate	subseptate	subseptate	subseptate	subseptate
Seed number, shape	1–3; ovoid; 30–38 × 20–35 × 20–26 mm thick, sometimes laterally compressed	2–5; ellipsoid; 9 - 12 × 5–8 × 3–6 mm thick; seeds separated in pod, enclosed in lomented endocarp with a flat wing	1–5(8) ; orbicular; 12–14 × 12–14 × 12 mm	4–10; ellipsoid; 5–7.5 × 3.5–5 × 2.5–4.5 mm thick, separate in pod	(1)6–8, lenticular , suborbicular, oblate-spheroid, smooth, brown 5–28 × 4–20 × 1–5 mm, separate in pod	2–10, lenticular, ovoid to flat, 7–28 × 5–20 mm, 1–7 mm thick, separate in pod	1–6; flattened-lenticular to ovoid or globose; 7–30 × 6–35 × 0.5 - 20 mm, separate in pod	2–3, flattened, orbicular, 17 × 17 × 10 mm, smooth	1–6, lenticular, 10–12 × 9–11 × 3–5 mm, separate in pod	1–3; ovoid to elliptic; 12–45 × 14–35 × 8–30 mm thick, if more than one then seeds becoming fused together	2–3; flattened ellipsoid-orbicular; 15–25 × 10–14 × 8–13 mm thick, seeds separate in pod	1–2; obovoid or oblong; 50–80 × 40–45 × 30–45 mm, if more than one, seeds often fused together or forced laterally out of shape by compression	(1)2–6; ellipsoid or broadly ovoid; 12–43 × 12–42 × 12–41 mm, if more than one, seeds sometimes forced out of shape by lateral compression	1–5; lenticular-orbicular; 8–10 × 8–12 × 2–4 mm (*W.frutescens* 4–8; reniform-cuboid; 8–10 × 4–6 × 4–6 mm thick)
Seed hilum (elliptic to oval or strap-shaped) and size (short - 1–5 mm long; long - 10–40 mm)	2–3 × 2 mm; circular to elliptic, short	1.5–3 × 2 mm; broadly elliptic, short	1.6–2 mm long, elliptic, short	2–2.5 × 1 mm; elliptic, short	1–2 × 1 mm; elliptic, short	2 - 3 × 1 mm, elliptic, short	2–5 × 1 mm; elliptic or oval, short	1–2 mm; elliptic, short	1–2 × 0.5–1 mm, elliptic, short	3–5 × 1.5 mm; broadly elliptic, short	**15–30 × 2–4 mm; strap-shaped, long**	**18–36 × 4–7 mm; strap-shaped, long**	**16–30 × 2–4 mm long; strap-shaped**	1–2 × 1 mm; linear or elliptic, short (3–4 mm, broadly elliptic in *W.frutescens*)
Seed wing	absent	**present, seed enclosed in lomented endocarp with a flat wing**	absent	absent	absent	absent	absent	absent	absent	absent	absent	absent	absent	absent

**^1^** (*A.atropurpureus*,*A.vastus*); **^2^** (*E.racemosa*); **^3^** (*S.kityana*); **^4^** (*S.scandens*, *S.bicolor*, *S.solomonensis*); **^5^** (*W.japonica*, *W.championii*, *W.kiangsiensis*, *W.reticulata*, *W.eurybotrya*); **^6^** (*N.fordii*, *N.speciosa*); **^7^** (*C.nitida*, *C.bonatiana*, *C.cinerea*, *C.cochinchinensis*, *C.dielsiana* & including all segregates in Wei and Pedley (2010)); **^8^** (*S.strobilifera*); **^9^** (*K.tenasserimensis*, *K.chlorantha*); **^10^** (*W.scandens*, *W.eriantha*, *W.nieuwenhuisii*, *W.sumatrana*); **^11^** (*A.sericea*, *A.mahidoliae*); **^12^** (*P.dasyphylla*, *P.filipes*, *P.maingayi*); **^13^** (*A.australis*, *A.megasperma*, *A.pilipes*); **^14^** (*W.frutescens*, *W.brachybotrys*, *W.floribunda*, *W.sinensis*)

**Table 5.** Morphological characters distinguishing *Afgekia* and *Padbruggea*. Comparison is made of critical characters of these genera in support of the transfer of *A.filipes* to *Padbruggea*.

List of characters	* Padbruggea dasyphylla *	*Padbruggea* (=*Afgekia*) *filipes*	* Afgekia sericea *	* Afgekia mahidoliae *
Stem sap colour	blood red	blood red	colourless	colourless
Stipule type	4–8 mm, ovate-lanceolate, caducous	1–2.5 mm; deltoid; caducous	15–25 mm; ovate-lanceolate; persistent	10–20 mm; ovate-lanceolate; persistent
Leaflet number	9 –17	13–19	15–17	9 –11
Inflorescence type	multi-branched panicle	multi-branched panicle	simple axillary raceme	simple axillary raceme
Inflorescence axis	robust, thickened, woody,	robust, often thickened and becoming woody	slender, not thickened or woody	slender, not thickened or woody
Pedicel length	4–7 mm long	15–25 mm long	7–20 mm long	7–10 mm long
Calyx teeth	acute; 1–3 mm long	obtuse; 3–6 mm long	linear-lanceolate; 4–17 mm long	linear-lanceolate; 5–15 mm long
Floral bracteole	3–6 mm long; caducous	0.5–1 mm long; caducous	absent	absent
Floral bract type	4–5 mm long, ovate; apex acute; as wide as flower buds at anthesis	15–20 mm long; broadly ovate; apex acute; wider than flower buds prior to anthesis	20–35 mm long; lanceolate; apex attenuate; narrower than flower buds at anthesis	15–30 mm long; lanceolate; apex attenuate; narrower than flower buds at anthesis
Floral fragrance	fragrant	fragrant	scentless	scentless
Callosities on standard petal	1 pair; ridge type	1 pair; papillate	2 pairs; 1 papillate, 1 corniculate	2 pairs; 1 papillate, 1 corniculate
Wing petal appendage at base	one claw	one claw	two claws	one claw
Keel petal shape and pubescence	white or pale pink; glabrous; falcate	white or lilac; glabrous; cochleate	white; densely pubescent; naviculate	white; densely pubescent; naviculate
Filament hairs above and below anthers	absent	absent	present	present
Pod size, shape and surface ornamentation	100–170 x 50–90 mm; oblong; apex obtuse; velutinous; obliquely ridged	170–250× 50–110 mm; ovoid-fusiform; apex obtuse; velutinous; obliquely ridged and furrowed	70–150 × 30–40 mm; ellipsoid-obovoid; apex acute; velutinous; smooth, lacking ridges	60–90 × 30–35 mm; ellipsoid-obovoid; apex acute; velutinous; smooth, lacking ridges
Seed size, number per fruit, shape and surface texture	40–50 × 30–40 mm; 1 or 2; oblong, testa smooth	60–80 × 40–50 mm; 1, rarely 2; oblong-orbicular, testa rugose or wrinkled	15–20 × 10–12 mm; 2 or 3; lenticular-orbicular, testa smooth, glossy	18–25 × 12–14 mm; 2 or 3; lenticular-orbicular, testa smooth, glossy
Hilum shape and length	18–20 x 4–5 mm; narrowly elliptic; 1/3 circumference of seed	20–36 × 5–7 mm; narrowly elliptic; 1/6 to 1/8 circumference of seed	15–22 × 2–4 mm; ligulate; 1/2 circumference of seed	18–30 × 2–4 mm; ligulate; 1/2 circumference of seed

## Results

New diagnoses (emended where necessary) – and full descriptions – are given for all genera in the taxonomic treatment section, because nearly all established genera have been modified over various historical treatments to include and/or exclude species such that their present concepts are often significantly different from the original protologue. Keys to genera and to all species (excluding those Chinese taxa of *Calleryas.str.* that we were unable to access) and extensive synonymy and typifications are also provided.

**Figure 1.**
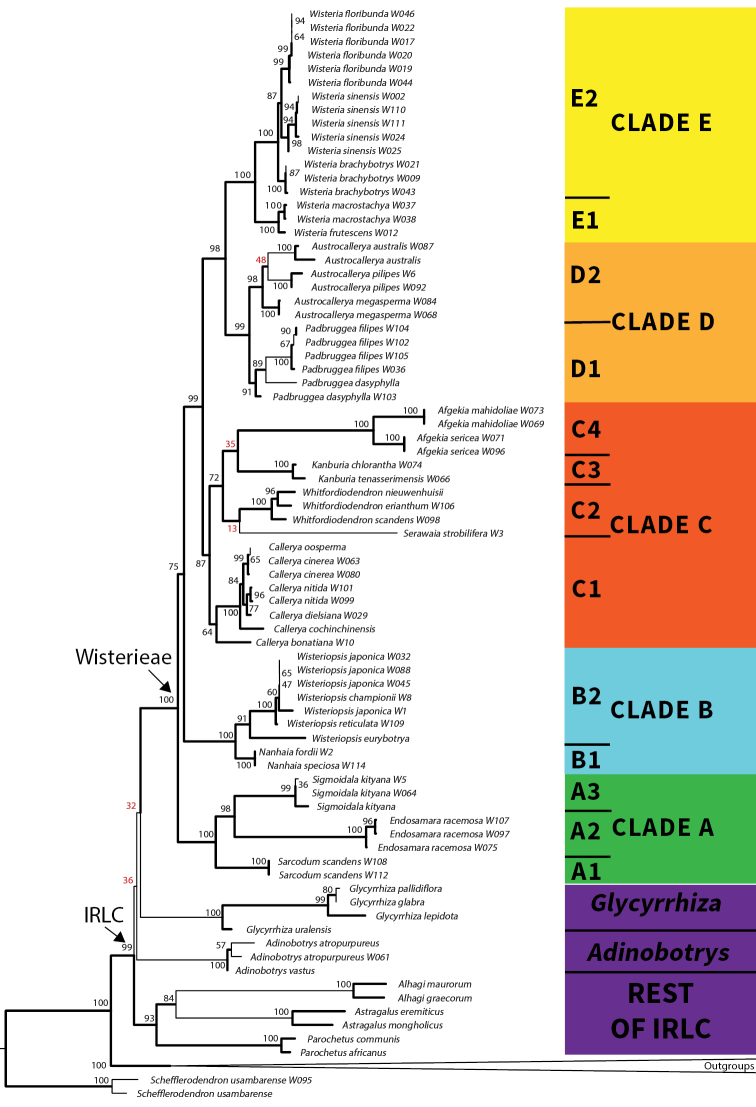
Combined Maximum Liklihood (ML) and Bayesian Inference (BI) Phylogenetic tree of Tribe Wisterieae. The tree is derived from the combined plastid and ITS, RAxML bipartitions analysis representing 77 (36) ingroup samples (taxa) and 59 (40) outgroup samples (taxa). The outgroup *Schefflerodendron* is used to root the trees. Lines in bold on the phylogeny incorporate results from the combined Bayesian Inference analysis, demarcating clades with BPP (0.95) support and above. Nodes are marked up with bootstrap values as percentages derived from the combined ML analysis with values of 50% or less marked in red. The collapsed portion of the tree, below the IRLC and above *Schefflerodendron*, represents the following genera (see Suppl. material 1: Figs S1–S6): Tribe Robinieae (*Coursetia, Gliricidia, Poissonia* & *Poitea*); Tribe Sesbanieae (*Sesbania*), Tribe Loteae (*Coronilla, Lotus* & *Securigera*); Tribe Millettieae (*Millettia*), Tribe Abreae (*Abrus*); Tribe Phaseoleae (*Clitoria* & *Ophrestia*); Tribe Indigofereae (*Phylloxylon*) and basal millettioids (*Austrosteenisia*, *Disynstemon*, *Xeroderris* & *Platycyamus*). Tribe Wisterieae is treated within five clades (Clades A–E), colour coded green for Clade A (*Sarcodum*, *Endosamara* & *Sigmoidala*); cyan for Clade B (*Nanhaia* & *Wisteriopsis*), red for Clade C (*Callerya*, *Serawaia, Whitfordiodendron, Kanburia* & *Afgekia*); orange for Clade D (*Padbruggea* & *Austrocallerya*) and yellow for Clade E (*Wisteria*). Each clade is further subdivided to represent the genera (except for the single accession of *Serawaia* which is incorporated with *Whitfordiodendron* in Clade C2) and E1 and E2 represent the geographical disjunction of species in *Wisteria*. Outgroups within the IRLC in purple include *Glycyrrhiza*, *Adinobotrys* and representatives of the Temperate Tribe block. The ingroup (IRLC) and Tribe Wisterieae are demarcated with arrows on the tree.

The combined analyses are consistent with respect to their ingroup topologies and are combined in the reference phylogenetic tree of this paper, Fig. 1 (see also Suppl. material 1: Figs S1, S2). In the plastid analyses (Suppl. material 1: Figs S3, S4) the main difference to Fig. 1 is *Serawaia* grouping with Clades D + E with no support in the BI analysis - and with Clades C + D + E, with no support, in the ML analysis. The ITS BI analysis is similar to Fig. 1 except for the merging of Clades B + C. In the ITS ML analysis Clade A also breaks down together with Clades B + C as *Endosamara* is attracted into Clade C from Clade A. As an indication of variability across the four genes, the average percentage identity over the alignments (i.e., pairwise percentage of identity) is: plastid vs. ITS analyses (92.5%, 77.1%) and within the plastid analyses, *matK*, *rbcL* and *ndhJ-trnF* (95.4%, 96.9% and 86.6%).

The *Callerya* group *sensu*Schot (1994); Lôc and Vidal (2001); Wei and Pedley (2010) and Sirichamorn et al. (2016) is not supported in its entirety in our analyses but rather, what emerges are four elements comprising the IRLC, each fully supported here (Fig. 1; Table 3), i.e. Tribe Wisterieae, *Glycyrrhiza*, *Adinobotrys* and a clade containing the Galegeae*s.l.*, Hedysareae, Cicereae, Fabeae and Trifolieae*s.l.* (henceforth the “Temperate Tribe block”). *Glycyrrhiza* (Bootstrap or BS 100%; Bayesian Posterior Probability or BPP 1) is not supported as sister to Tribe Wisterieae and neither is *Adinobotrys* (BS 100%; BPP 1), which, in addition, is not supported to have a position within Tribe Wisterieae either. *Adinobotrys* is thus reinstated as a genus here and removed from the *Callerya* group. The positions of Tribe Wisterieae, *Glycyrrhiza* and *Adinobotrys* remain equivocal as regards their sister group relationships to the rest of the Temperate Tribe block of the IRLC.

The *Callerya* group i.e. Tribe Wisterieae without the genus *Adinobotrys* comprises five strongly supported clades with the first two in a basal grade leading to Clades C + D + E. The crown node of the tribe is fully supported in both the combined and plastid analyses (BS 100%; BPP 1) and in the ITS BI analysis (BPP 1), although only weakly so in the ITS ML analysis (Fig. 1, Table 3, BS 61%). Clade A (fully supported in the combined and plastid analyses but weakly so in the ITS analyses [BS 59%, BPP 0.86]) contains the genus *Sarcodum* (BS 100%; BPP 1), sequenced and analysed for the first time here, which is sister to *Endosamara* (BS 100%; BPP 1) and the new monospecific genus *Sigmoidala* (BS 99%; BPP 0.99) described in this paper. Clade B comprises two new genera described here, *Nanhaia* (BS 100%; BPP 1) and *Wisteriopsis* (BS 91%; BPP 1) and both are fully supported as Clade B (Fig. 1; Table 3). Our results confirm that the incorrectly attributed *Millettiajaponica* is strongly supported within our new genus *Wisteriopsis*.

Clade C is strongly supported (BS 87%; BPP 0.98) in the combined analyses but is more labile with some genera excluded and others included in the plastid and ITS analyses (Fig. 1; Table 3). A much reduced *Calleryas.str.* together with *C.bonatiana* is strongly supported in the combined and plastid BI analyses (BPP 0.95; 0.96), but only weakly so in the ML analyses. The single accession of *C.bonatiana* has no support for grouping with *Callerya* in the ITS analyses. *Callerya* above *C.bonatiana* is strongly supported in the combined ML and BI analyses (BS 100%; BPP 0.95). The grouping of *Afgekia* (BS 100%; BPP 1), the resurrected genus *Whitfordiodendron* (BS 100%; BPP 1) and the two new genera described here, *Kanburia* (BS 100%; BPP 1) and *Serawaia* (single accession), is moderately supported in the combined ML (BS 72%) and well supported in the combined BI (BPP 0.99) analyses. This grouping breaks down in the plastid analyses and is weakly supported in the ITS analyses (Table 3). *Serawaia* is strongly supported within Clade C in the combined analyses (BS 87%; BPP 0.98) in a position (Fig. 1, Table 3), with no support, as sister to *Whitfordiodendron*. It is in an unresolved position in the plastid analyses and is weakly supported as sister to *Kanburia* in the ITS analyses (Table 3).

Clade D comprises two genera, *Padbruggea* (BS 91%; BPP 1) which is reinstated as a genus here and *Austrocallerya* (BS 98%; BPP 1), a new genus described here. Our results reveal that *Afgekiafilipes* belongs in our reinstated genus *Padbruggea* and the transfer back is made in this paper. The two genera are also strongly supported together as Clade D (BS 98%; BPP 1). Clades D + E are strongly supported in all analyses (combined ML [98%] & BPP [1]; plastid BPP [0.97] and ITS ML [90%] & BPP [1]), but in the plastid ML analysis support is weak (BS 68%). Finally Clade E comprises *Wisteria* (BS 100%; BPP 1), with two North American taxa fully supported as sister to the three Asian species of the genus (BS 100%; BPP 1). The relationship of *W.brachybotrys* as sister to *W.floribunda* and *W.sinensis* is also fully supported (BS 100%; BPP 1).

## Discussion

### Morphology of the *Callerya* Group

Schot (1994) segregated her species of *Callerya* into two groups based on the presence or absence of stipels and, when present, whether they were persistent or caducous. We have found no evidence that stipel presence or absence has any taxonomic significance in *Calleyas.l.*Lôc (1996) and Wei and Pedley (2010) segregated species on the basis of the presence or absence of an indumentum on the dorsal surface of standard petals. We concur with Lôc (1996) and Wei and Pedley (2010) but in addition regard the inflorescence type and various floral, fruit and seed types to be equally significant in delimiting taxa (see the Key to the Genera and Table 4 for a list of significant characters). The key to fruiting specimens of *Calleryas.l.* (Lôc 1996: 56) emphasised stipellae characters as distinctive, an observation for which we find no support.

Using our revised generic concepts and species assigned to them (Table 1) and comparison of morphological characters (Table 4), the synapomorphies diagnosing the *Callerya* group are: the lianescent habit (except for the tree species *Adinobotrysatropurpureus* and *A.vastus*); flowers inserted singly on the axis in either axillary or terminal racemes and/or panicles, and bracts either fully or largely enclosing the flower buds at the inflorescence apex, which are usually longer and often wider than the buds. There are, however, some exceptions. Floral bracts are caducous at anthesis in most of the *Callerya* group except in *Adinobotrysatropurpureus*, *Nanhaiaspeciosa*, *Serawaiastrobilifera* and *Wisteriopsis* where they are persistent.

**Figure 2.**
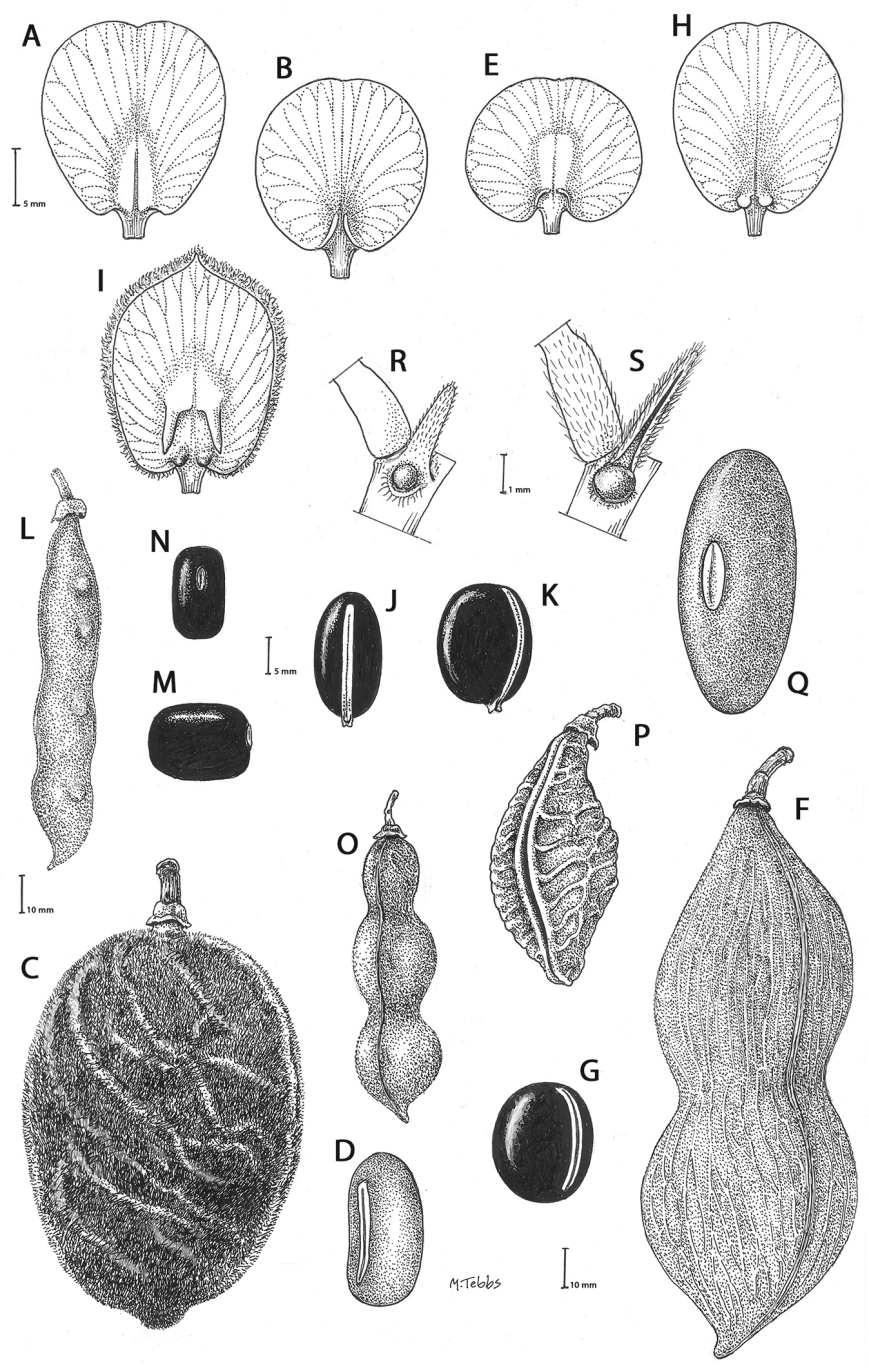
Distinctive morphological characters in Tribe Wisterieae. **A***Endosamararacemosa* standard petal inner surface **B***Padbruggeadasyphylla* standard petal inner surface **C***Padbruggeadasyphylla* pod **D***Padbruggeadasyphylla* seed lateral view **E***Austrocalleryaaustralis* standard petal inner surface **F***Austrocalleryapilipes* pod **G***Austrocalleryapilipes* seed lateral view **H***Padbruggeafilipes* standard petal **I***Afgekiasericea* standard petal inner surface **J***Afgekiasericea* seed lateral view **K***Afgekiasericea* seed angled lateral view **L***Calleryanitida* pod **M***Calleryanitida* seed ventral view **N***Calleryanitida* seed polar view **O***Calleryacinerea* pod **P***Whitfordiodendronnieuwenhuisii* pod **Q***Whitfordiodendronerianthum* seed **R***Wisteriopsiseurybotrya* gibbosity **S***Wisteriopsischampionii* gibbosity **A** from *Luang Vanpruk* 188 **B** from *Scortechini* 429 **C, D** from *Lamb* 395/91 **E** from *L.J.Brass* 32129 **F, G** from *B.Gray* 04319 **H** from *Maung Po Khant* 15326 **I** from *C. Chermsirivathana* 996 **J, K** from *Mrs Collins* 104/9 **L–N** from *Theophilus Sampson***O** from *G.Forrest* 19279 **P** from *J.P.Mogea* 4182 **Q** from photo *Y.Sirichamorn s.n..***R** from *J. & M.S.Clemens* 3637 **S** from *Shiu Ying Hu* 10476. See Appendix 1 for voucher details. Drawn by Margaret Tebbs.

Gibbosities, which are small protuberances that develop beneath the leaf pulvinus above the stipule where it is attached to the stem, are absent in most of the *Callerya* group but are present in both *Wisteriopsis*, *Nanhaia* and *Serawaia* (Table 4; Fig. 2R–S). Bracteoles may be found either at the base of the calyx or along the pedicel. They are present in most genera but absent in *Endosamara*, *Sigmoidala*, *Kanburia* and *Afgekia*. They are also absent in *Wisteriafrutescens* (Table 4).

Genera in the *Callerya* group often differ from each other (Table 4, Fig. 2) according to the presence of callosities at the base of the standard petals. Callosities occur in five distinct types:

a) **Boss** callosities form two slightly raised domes or swellings on either side of the midline of the standard lamina, at the point of its upward flexion above the claw (Fig. 2A). The standard in the latter case often appears to be smooth but the bosses hold the two wing petals close to the standard prior to anthesis. Boss callosities are found in *Adinobotrys, Endosamara, Sigmoidala, Sarcodum, Nanhaia, Serawaia* and *Wisteriopsis* and occasionally in *Callerya* and *Whitfordiodendron*;

b) **Arched** callosities are paired half-moon or crescent shaped arches forming ridges of hardened tissue that curve up from the base towards the midline over the staminal sheath (Fig. 2E). These are found only in *Austrocallerya*;

c) **Ridge** callosities form a straight ridge or rim of hardened tissue on either side of the midline of the standard near the base (Fig. 2B) and are seen in most *Calleryas.str.* species, *Whitfordiodendron*, *Kanburia*, *Padbruggeadasyphylla* and in the North American *Wisteriafrutescens*;

d) **Papillate** callosities are those where a pair of papillate projections protrude from the area of hardened tissue on the surface usually at the point of upward flexion of the standard lamina above the claw (Fig. 2H). These are present in *Afgekia, Padbruggeafilipes* (see Dunn 1911: 195 as *Adinobotrysfilipes*) and all the Asian species of *Wisteria* (see Valder 1995: 26 as “auricles”);

e) **Corniculate** callosities are present in the two species of *Afgekia* (Fig. 2I). Uniquely in the *Callerya* group, these two species have, in addition to a basal papillate pair, a second pair of corniculate or horned callosities which project out from the lamina above the basal pair.

There are notable differences in the fruits and seeds among the genera. In *Endosamara* and *Sarcodum* the exocarp separates from the endocarp and some degree of separation also occurs in *Wisteriopsis*. In *Endosamara* the pods are clearly septate with transverse walls between each seed, forming loments (see *Endosamararacemosa* in Geesink 1984: 63 Pl. 1, 5; Lôc and Vidal 2001: 17, Pl. 3). In *Sarcodum* the sausage- shaped or botuliform pods which initially have a fleshy exocarp, are also fully septate but do not form loments (see *Sarcodumscandens* in Geesink 1984: 63 Pl. 1, 7; Lôc and Vidal 2001: 9, Pl. 1). The North American *Wisteriafrutescens* has nonseptate pods. In all other genera in the *Callerya* group the endocarp is subseptate with seeds making indentations in the surrounding pith while areas between the seeds appear as irregular, often indistinct transverse septa. In *Afgekia* the funicle as well as the hilum (see *Afgekiasericea* funicle in Geesink 1984: 64 Pl. 2, 10; Fig. 2J–K) are both significantly longer than those of other taxa in the group with the exception of *Padbruggea* (Fig. 2D) and *Austrocallerya* (Fig. 2G).

### *Callerya* group taxonomy 1: The genus *Callerya* including genera previously placed in synonymy prior to this study

***Callerya* Endl., Gen. Pl. Suppl. 3: 104 (1843)**

Endlicher (1843) first described the genus *Callerya* based on *Marquartiatomentosa* Vogel, which had been described that same year from southern China (Vogel 1843: 37). Endlicher used the new name *Callerya* to replace Vogel’s *Marquartia*, the replacement name being necessary because the generic name *Marquartia* Hassk. had already been provided a year earlier for a species of *Pandanus* by Justus Karl Hasskarl, assistant curator of the Buitenzorg Botanic Garden [Bogor] on Java (Hasskarl 1842: 14). Hasskarl’s *Marquartia* is moreover, now considered as synonymous with *Pandanus* L.f. (1782).

In addition, the description and illustration of the species on which Vogel based his new but superfluous generic name *Marquartia* [i.e. *M.tomentosa*] (Vogel 1843) had already been described by Bentham as *Millettianitida* Benth. (Bentham 1842: 484). Vogel’s illustration clearly shows the leaves with persistent stipules, each with five leaflets, long persistent floral bracts and densely sericeous ovary, all characters diagnostic of that species. This meant that not only the generic name *Marquartia* but also *Calleryatomentosa* (Vogel) Endl. had to be replaced according to Art. 11.4 of the ICN (Turland et al. 2018). The replacement *Calleryanitida* (Benth.) Geesink was published formally by Geesink within his revision of Tribe Millettieae (Geesink 1984: 82). *Calleryanitida* is thus the type species of the genus *Callerya*. Endlicher’s description of *Callerya* mentioned the compressed, woody and leathery pod which he stated contained either a few seeds or a single seed which was ovoid-circular and flattened-compressed. *Calleryanitida* (Fig. 2L–N) does indeed possess flattened pods with up to ten compressed seeds (see Schot 1994: 28, Fig. 3). The species may also become a scandent shrub but is not arborescent as Endlicher implied.

**Figure 3.**
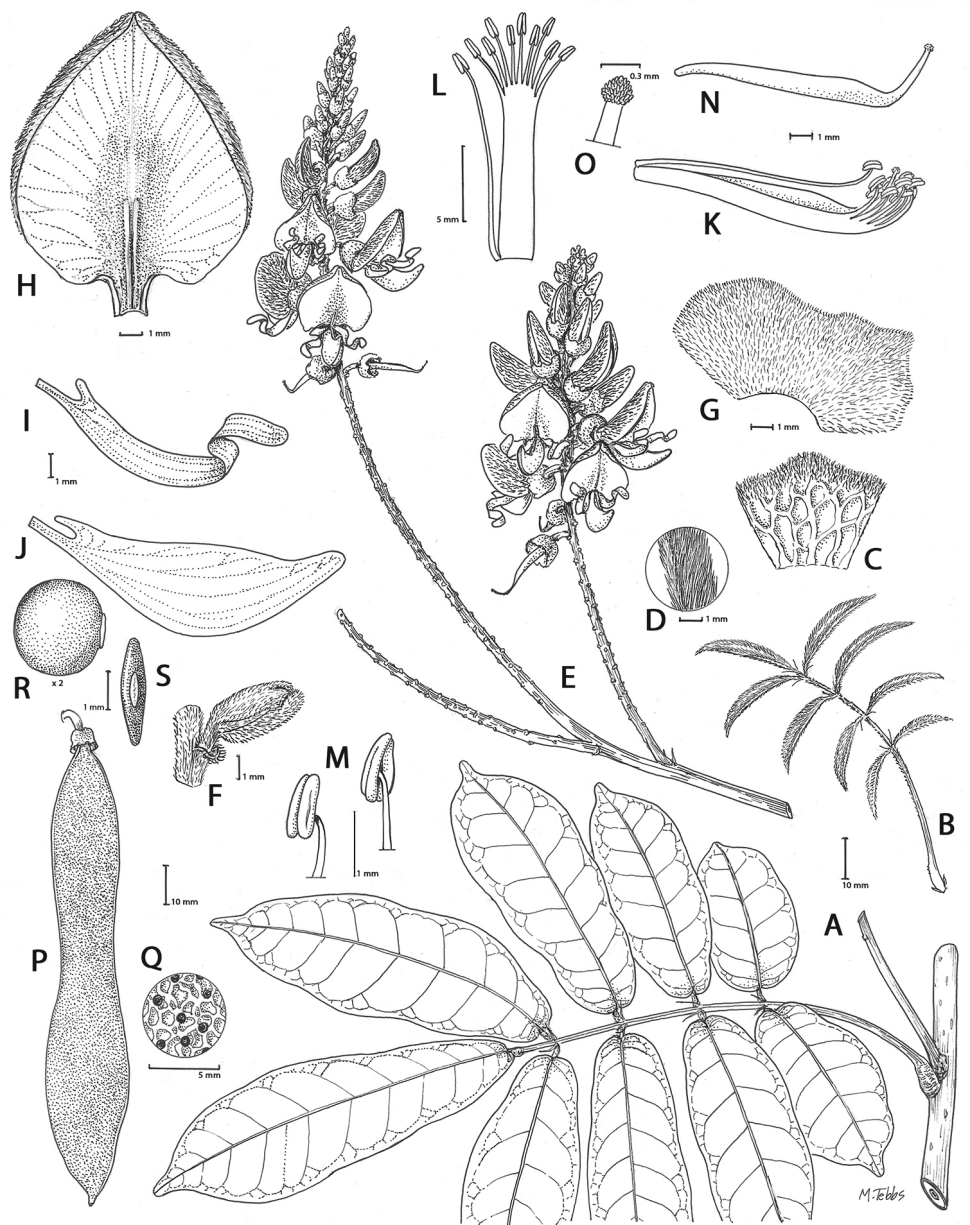
*Sigmoidalakityana* (Craib) J.Compton & Schrire. **A** Habit **B** young leaf **C** lower surface of leaf **D** leaflet detail of hairs **E** inflorescence **F** flower bud with bracteole and pedicel **G** calyx external surface **H** standard petal inner surface **I** wing petal **J** keel petal **K** staminal column lateral view **L** staminal column ventral view **M** stamen dorsal and ventral views **N** ovary lateral view **O** style and stigma **P** pod **Q** pod detail of surface **R** seed ventral view **S** seed lateral view (all from *Clark* 245). Drawn by Margaret Tebbs.

Geesink (1984) recognised that species of *Callerya* had true paniculate inflorescences. He also sank three genera; *Adinobotrys* Dunn, *Padbruggea* Miq. and *Whitfordiodendron* Elmer into his redefined *Callerya* and transferred two Sections created by Dunn (1912a) from Millettia–Sect.EurybotryaeDunn andSect.Austromillettia Dunn – into *Callerya* and created a new monotypic genus EndosamaraGeesink fromSect.Bracteatae Dunn based on *Robiniaracemosa* Roxb. (1832).

*Callerya* was revised by Schot (1994) and was treated as belonging in Tribe Millettieae (see Table 1). Schot recognised 19 species from China, south-east Asia and Australasia and since then many more species have been described, bringing the total number to 33 (Sirichamorn et al. 2016). All species *sensu*Schot (1994) except *C.atropurpurea* and *C.vasta*, are vigorous climbing or scandent woody shrubs. Inflorescences are paniculate with either axillary racemes or secondary panicles and frequently possess rather thick to woody inflorescence axes with prominent bud scars. Floral bracts are generally short and can be narrow or broad according to species and are in most species caducous, rarely persistent. Flowers may be white, green, red, brownish-yellow, lilac, pink or deep purple. In the type species *Calleryanitida*, the wings are distinctly shorter than the keel. Fruits, which can be either flattened or inflated are velutinous and ribbed, occasionally wrinkled or smooth and the seed chambers are subseptate. Seeds 2–9, large, ovoid to ellipsoid (Tables 1, 4).

Schot (1994: 2) chronicled the transfer of species from other genera into *Callerya* and included eleven species from *Millettia*, the first two of which were *Pterocarpusaustralis* Endl. Prod. Fl. Norfolk (Endlicher 1833: 49) and *Pongamiaatropurpurea* Wall. (Wallich 1830: 70).

Although the genus *Pterocarpus* is placed in Tribe Dalbergieae (Klitgaard and Lavin 2005), the species *P.australis* has been shown to belong in the *Callerya* group (Li et al. 2014). *Pongamia* Adans. is now treated as being synonymous with *Millettia*, with the type *P.pinnata* (L.) Pierre being transferred to *Millettiapinnata* (L.) Panigrahi by Panigrahi and Murti (1989). *Pongamiaatropurpurea* which was transferred into *Callerya* by Schot (1994: 15) has also been found to belong in the *Callerya* group (Li et al. 2014). Schot (1994) further transferred the Australian species originally described as *Wisteriamegasperma* F.Muell. (1858) into *Callerya*. This too has been confirmed to belong within the *Callerya* group (Li et al. 2014).

In their analyses using combined data from chloroplast *trnK* and *matK* sequences, Hu et al. (2000) showed that *Calleryareticulata* was sister to a clade supported by BS 100% with *Wisteriafrutescens* and *W.sinensis*. In a later paper using sequence data from nuclear DNA ITS spacers and a larger sampling of *Callerya* and *Wisteria* as well as *Afgekiafilipes*, Hu et al. (2002) found that *Callerya* was polyphyletic occurring in four different clades. *Wisteriafrutescens* was strongly supported sister to *W.brachybotrys*, *W.sinensis* and *W.floribunda* with BS 100%. The *Wisteria* clade was sister to a clade with *Calleryamegasperma*, *C.australis* and *Afgekiafilipes* with strong BS (85%) support. In a later analysis Hu and Chang (2003) using the more conserved *rbcL* chloroplast gene, found that *Calleryavasta* was early branching to a clade of *Wisteriasinensis* sister to *Afgekiasericea* while *Endosamararacemosa* was sister to *Millettiajaponica*.

Schot included nine synonyms within her concept of *C.cinerea*, a species that she recognised to have a wide distribution from Nepal in the west to the Chinese coast in the east (Schot 1994: 17). In our analyses we have utilised two specimens of *Calleryacinerea*, one from Thailand, the other from China, (Table 2) but these sheets lack diagnostically significant fruiting material and may not equate fully with the holotype material seen at Kew from Bangladesh, Sylhet, Chittagong, (*Wallich* 5888; K000881022). Within the *C.cinerea* complex, leaflet number, pod thickness and seed shape and number appear to be important characters. Wei and Pedley (2010) split *C.cinerea* into groups of species based on leaflet number: 3–5 in *C.tsui, C.dorwardii* and *C.sphaerosperma* and 5–7 in the other seven species. Wei and Pedley (2010) also segregated the species on the degree of pod inflation: flattened with lenticular seeds in *C.congestiflora*, *C.dielsiana* and *C.longipedunculata*; inflated with globose seeds in *C.cinerea*, *C.dorwardii*, *C.gentiliana*, *C.oosperma* and *C.sericosema*. We have only been able to sample material of two of the resurrected species recognised by Wei and Pedley (2010) that were included in *C.cinerea* by Schot (1994), namely *C.dielsiana* and *C.oosperma*. A further investigation of this group of Chinese species is needed to fully assess species delimitations. If all these species belong together with *C.nitida* and *C.cochinchinensis* as indicated both by Li et al. (2014) and from our preliminary results here, it seems that *Calleryas.str.* might comprise as many as twelve species.

***Padbruggea* Miq. Fl. Ned. Ind. 1(1): 150 (1855)**

Miquel (1855: 150) described the genus *Padbruggea* including the statement:

“*legumen oblongum, stipitatum crassum? exalatum*” [*legume oblong, possibly on a thickened stipe and not winged*] and he particularly noted the presence of callosities on the standard petal “*vexillum infra medium quidem texturae crassioris ac perinde subfoveolatum*” [*standard with a thickened area just below the middle with a somewhat pitted texture*]

Reference to the unwinged nature of the fruit was in comparison to some species of *Pterocarpus* Jacq. (Tribe Dalbergieae) whose fruits have a distinct wing-like exocarp. Miquel also noted that he had not seen mature fruits.

Our examination of the species described as *P.dasyphylla* Miq. (1855), revealed that the pods were readily distinguished from others in the *Callerya* group by their inflated but broadly flattened-cuboid shape with distinct longitudinal ridges and furrows and by the 1 or 2 compressed obovoid seeds possessing long strap-shaped hila 18–36 × 4–7 mm (Table 5).

In his protologue of *Adinobotrysfilipes*, Dunn (1911: 196) noted that in its appearance the species had more slender pedicels than those in what he considered to be the closely related *A.erianthus* (here in *Whitfordiodendron*) and that it approached *Padbruggea* in its auriculate standard (Dunn 1911). Dunn’s latter reference may also refer to the papillate callosities present on the standard of *A.filipes* but which are absent on the smooth standard of *A.erianthus*. In her monograph on *Callerya*, Schot (1994: 3) commented:

*Craib (1928) argued that Padbruggea and Whitfordiodendron were congeneric. He based his arguments on the intermediate position of Adinobotrysfilipes Dunn (now Afgekiafilipes Geesink). This species resembles in habit mostly Padbruggeadasyphylla, but has the generic characters of Adinobotrys*.

Craib recombined *Adinobotrysfilipes* in *Padbruggea*, along with a good measure of uncertainty as to whether he believed the species really belonged in that genus or in *Adinobotrys* (Craib 1928: 397). He also postulated that Elmer’s *Whitfordiodendron* may belong in *Padbruggea* thereby highlighting the morphological difficulties with respect to these taxa faced by later workers such as Geesink (1984).

Schot in her synonymy of *Calleryadasyphylla* also included *Milletiaoocarpa* Prain, distinguished from *P.dasyphylla* by its ovoid as opposed to compressed obovoid fruits and *M.maingayi* Baker which differs in its more numerous, smaller and more densely tomentose leaflets (Schot 1994: 20). We have recognised this as *Padbruggeamaingayi* in this paper (see below).

The status of *Afgekiafilipes* has long been debated as it has true panicles as opposed to racemes and shorter calyx teeth than those of the other two species of *Afgekia* (see Table 5). It also has a single pair of papillate callosities on the standard as opposed to two pairs found in both *A.sericea* and *A.mahidoliae*. *Afgekiafilipes* has entirely glabrous anthers as opposed to anthers with a basal tuft of hairs and it has much larger fruits and seeds (Table 5). It was originally described as *Adinobotrysfilipes* Dunn on the basis of its large single seeded pods (Dunn 1911: 195).

Geesink (1984: 76) transferred *Adinobotrysfilipes* into *Afgekia* adding:

*the general habit, the shape of the calyx, and the glabrous anthers are indeed similar to certain species of Padbruggea. It differs in the absence of bracteoles and the long pedicels. The pods were unknown until 1975, but then it appeared that the seeds showed an elongated fleshy funicle with a corresponding elongated hilum*.

Geesink (1984: 76) concluded that the morphology of *A.filipes* indicated that it was a less derived species than *A.sericea* and *A.mahidoliae* and alluded to its affinities with *Padbruggea* within which it had been placed by Craib (1928) along with three other species in *Adinobotrys*.

In his transferral of the species into *Afgekia*, Geesink noted the apparent absence of bracteoles, the length of the pedicels and the elongated hilum on the seeds (Geesink 1984: 77). Both Lôc and Vidal (2001) and Wei and Pedley (2010) followed Geesink, maintaining the species in *Afgekia*. Sirichamorn (2006) examined 37 living specimens of *A.sericea*, 50 specimens of *A.mahidoliae* and 32 specimens of *A.filipes* from wild material in Thailand and found that *A.filipes* posseses bracteoles, that pedicel length among the species overlaps and that the overall size of the seeds are three times that of the other two species of *Afgekia*, i.e. c. 80 mm vs. 15–25 mm long (Tables 4, 5).

Prathepha and Baimai (2003) mentioned the existence of *Afgekiafilipes* in their RAPD and nucleotide sequence analyses of *A.mahidoliae* and *A.sericea*, although they did not state their reason for excluding the species. Sirichamorn (2006), based on morphometric and molecular data, clearly showed that *Adinobotrysfilipes* does not belong with *Afgekia* (Table 5).

We have examined material of both *Afgekiafilipes* and *Calleryadasyphylla* and agree that there are indeed similarities between the two species. We have confirmed Sirichamorn’s (2006) discovery that *Afgekiafilipes* does have short, linear bracteoles that are attached at the base of the calyx (Table 5). Both species possess inflated fruits with a velvety indumentum and oblique longitudinal ridges and furrows but those of *C.dasyphylla* are broader and flatter, with the dorsal midline flanked by two large folds or flanges that meet at the apex. Our results show that *Afgekiafilipes*, originally described by Dunn (1911) in *Adinobotrys*, belongs in the genus *Padbruggea* and it is reinstated in that genus here (see Taxonomic treatment below) following Craib (1928). The diagnostic characters of *Padbruggeafilipes* are shown in Table 5.

***Whitfordiodendron* Elmer. Leafl. Philipp. Bot. 2: 689, 743 (1910)**

Elmer (1910) in his protologue of the illegitimate but valid name *Whitfordiascandens* stated that he had only seen young not mature fruits but that they were “thick, hard, canescently velvety and 1-seeded”. He also noted the puberulent dorsal surface of the standard petal of the deep red flowers (Elmer 1910: 691). The generic name *Whitfordia* was already utilised for the fungal genus *Whitfordia* Murrill (Murrill 1908: 407), a synonym of *Amauroderma* Murrill (Murrill 1905: 366) and although *Whitfordia* Elmer (with *W.scandens*) was described entirely in English, together with that of the transfer to *Whitfordiodendron* as an erratum in an appendix to the same volume, the name is nevertheless still valid. Under the International Rules of Nomenclature adopted in Vienna in 1905 and Brussels in 1910, a Latin diagnosis was a requirement for valid publication of a name of a new taxon on or after 1 January 1908 (Art. 36). As a result of discrepancies and disagreements between the American Code of Botanical Nomenclature [see Bull. Torrey Bot. Club 34(4): 167–178 (1907)] and the *Vienna Rules* and *Brussels Rules*, a rapprochement was made in the *Cambridge Rules* (1935) changing the implementation date to 1935. As a result, Elmer’s *Whitfordiodendron* based on *W.scandens* Elmer is validly published (J. McNeill pers. comm.).

Despite the nomenclatural wrangles on the validity and usage of *Adinobotrys* versus *Whitfordiodendron* (discussed by Merrill [1934: 159] who independently concluded that Art. 38 of the *Cambridge Rules* validated Elmer’s *Whitfordiodendron*), Merrill proceeded to describe the new species *Whitfordiodendronsumatranum* Merr., which he stated was close to *W.myrianthus* (i.e. to *W.nieuwenhuisii* (J.J.Sm.) Dunn). Merrill (1934: 160) also made what he believed to have been four new combinations in *Whitfordiodendron* but he was evidently unaware that *W.atropurpureum*, *W.erianthum*, *W.myrianthum* and *W.nieuwenhuisii* had already been combined in *Whitfordiodendron* by Dunn (1912b: 364).

Our morphological examination has revealed that two species previously included within *Callerya* share a suite of characters with *Whitfordiodendronscandens*. The most notable characters are: a) the flowers borne on extremely short pedicels 0.5–3 mm long vs. (2–)3–8 mm long in *Calleryas.str.*; b) the inflated, ovoid, rugose to ridged or ruminate pods with 1–3 seeds (if more than one-seeded then these often becoming fused together, Fig. 2P–Q) vs. pods flattened or if inflated then convex around seeds and contracted between them, the seeds being separate in the pod in *Calleryas.str.*; and c) most significantly, the sericeous keel petals which are particularly densely hairy along their lower margins (keel glabrous in *Calleryas.str.*, see Table 4).

Based on nrDNA ITS sequence data, Li et al. (2014) showed that *Calleryaeriantha*, *C.scandens* and *C.nieuwenhuisii* formed a clade with BS (100%) which is sister to *C.eurybotrya* and *C.reticulata*.

***Adinobotrys* Dunn, Bull. Misc. Inform. Kew 1911(4): 194 (1911)**

The genus *Adinobotrys* was described by Dunn (1911: 194) in comparison to *Millettia* and *Padbruggea* with the statement:

“*affinis Millettieae Wight et Arn. sed ovario stipitato, legumine monospermo indehiscente differt [related to Millettiae Wight & Arn. but differs by having a stipitate ovary and indehiscent one-seeded pod*]”.

Dunn made a further distinction between *Padbruggea* (which he understood to comprise *P.dasyphylla* and *P.maingayi*) and *Adinobotrys*, stating that the inflorescence in *Padbruggea* was lax and that *Padbruggea* lacked any appendages on the wings and keel petals (Dunn 1911: 197). Dunn (1911) included five species in *Adinobotrys* without assigning any one of them as the type species; *A.erianthus* (Benth.) Dunn, *A.filipes* Dunn (see above), *A.nieuwenhuisii* (J.J.Sm.) Dunn, *A.myrianthus* Dunn and *A.atropurpureus* (Wall.) Dunn. Geesink (1984: 83) typified *Adinobotrys* on the species *A.atropurpureus*, the only species of the five which is a tree and not a liana. The following year Dunn (1912b) added *A.scandens* (Elmer) Dunn in the belief that Elmer (1910)had not validly published the name under *Whitfordiodendron*. Dunn, recognising the uncertainty of the validity of *Whitfordiodendron*, validated Elmer’s names firstly in *Whitfordiodendron* (including *W.scandens*) and then into his new genus *Adinobotrys* (Dunn 1912b: 364, 365).

Schot (1994) included *A.atropurpureus, A.erianthus* and *A.nieuwenhuisii* in *Callerya* and recognized *A.myrianthus* as conspecific with *C.nieuwenhuisii*. Schot did not include *A.filipes* in *Callerya* as she treated the species as belonging in *Afgekia* (Schot 1994: 3).

*Adinobotrys* has several morphological characters that separate it from the other allied genera within the *Callerya* group; trees vs. lianas, stipules 2–4 mm long, floral bracts short, c.1–3 mm long, standard petal glabrous (although this is not unique to *Adinobotrys*), pods inflated with glabrous, rugose surfaces and large ovoid seeds with short elliptic or circular hila (see Geesink 1984: 64 Pl. 2, 14).

Results from sequence data of nuclear ITS and chloroplast *matK* showed that *Calleryaatropurpurea* was placed sister to the rest of the *Callerya* group (Li et al. 2014).

### *Callerya* group taxonomy 2: Additional genera within the *Callerya* group as defined in treatments prior to this study.

***Sarcodum* Lour., Fl. Cochinch. 2: 462 (1790)**

The Portuguese Jesuit missionary and botanist João de Loureiro was the first to describe the genus *Sarcodum* in 1790 based on its seed pods which are fleshy when young [sarcos = Gk fleshy] (Plate 2G). The type species *S.scandens* was described as having rose-coloured flowers in simple spikes and was found growing in woods of Cochinchina, i.e. modern day Vietnam. It has since been collected in China on Hainan Island, in Indonesia and in the Philippines. In 2017 it was discovered by co-author S. Mattapha in Bolikhamxai Province in Laos. *Sarcodumscandens* has standard petals 10–13 × 6–8 mm and leaves with 17–45 leaflets. Two further species have recently been described; *S.bicolor* in 1999 with standard petals 13 × 8 mm and leaves with 9–15 leaflets from Sumba in the Lesser Sunda islands of Indonesia and *S.solomonensis* in 2008 from the Solomon Islands with standard petals 6 × 5 mm and leaves with 17–27 leaflets (Clark 2008: 156, Table 4).

Distinguishing characters are the many small sericeous, elliptic leaflets; long stipules; long caudate floral bracts; racemose inflorescences; flowers with campanulate calyces and five very short, acute teeth; glabrous standard petals with boss callosities, elongating persistent styles on the developing pods post anthesis, and fleshy, cylindrical, botuliform fruits becoming hard when mature. The glabrous exocarp dries to dehisce from the septate tan-coloured chartaceous endocarp in which lie the 4–10 ellipsoid to reniform seeds (Kirkbride et al. 2003, Table 4).

None of the three species of *Sarcodum* have been included in any DNA based phylogeny prior to this study, although its affinities with other genera in the *Callerya* group have been postulated (Geesink 1984; Lavin et al. 1998).

***Endosamara* R.Geesink Leiden Bot. Ser. 8: 93 (1984)**

**Plate 1.**
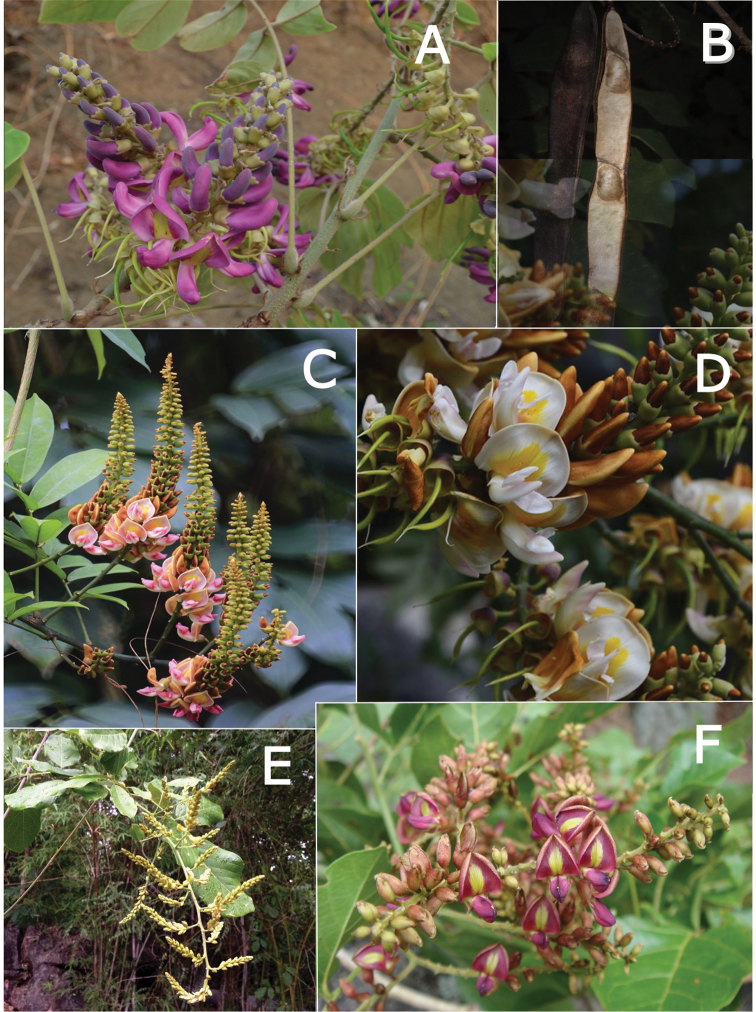
*Endosamara, Sigmoidala* and *Kanburia*. **A, B***Endosamararacemosa*, Thailand, Sakon Nakhon Prov., *S.Mattapha* s.n.. **C, D***Sigmoidalakityana* Thailand, Nan Prov. *S.Mattapha 1117***E***Kanburiachlorantha* Thailand, Kanchanaburi Prov. *Y.Sirichamorn Y2014-15-1***F***Kanburiatenasserimensis* Thailand, Ratchaburi, Khao Chon waterfall *Y.Sirichamorn YS2015-8*.

The unique monospecific genus *Endosamara* has never been included in *Callerya* but is nevertheless considered to be a close relative (Geesink 1984: 94). The Scottish surgeon and botanist William Roxburgh spent several decades in India where he described *Robiniaracemosa*, a climbing shrub from the Circar Mountains [Eastern Ghats] north of Madras, which had rose-coloured flowers in what he described as racemes but we now know to be panicles (Roxburgh 1832: 329). The species was introduced to the Calcutta Botanic Garden by Henry Colebrooke in 1803 from the Coromandel region of south-east India (Roxburgh 1814: 56). The single species has a widespread distribution across India, Laos, Myanmar, Philippines, Sri Lanka and Thailand. It was placed in *Millettia* by Bentham (1853) and was later recombined in *Wisteria* (Dalzell in Dalzell and Gibson 1861: 61). Dunn (1912a: 135) placed *Millettiaracemosa* in his monotypic Sect. Bracteatae, a taxon which was later recognised as *Endosamararacemosa* (Roxb.) Geesink (Geesink 1984: 93). Geesink recognised that the plant had some unusual characters (Table 4) that separated it from the genera in which it had previously been placed, notably that each of the 4 to 5 ellipsoid seeds is covered in a wing-like papery endocarp forming samaroid loments that enables the wind dispersal of each seed (see Plate 1B). The condition where a thin inner membranous layer surrounds the seeds forming loments is very rare among legume genera but is found in some species of the genus *Sesbania* Adans., *Entada* Adans. and in *Plathymenia* Benth. (Lavin and Schrire 2005: 452; Kirkbride et al. 2003: 332).

Chloroplast DNA data from the *rbcL* gene (Hu and Chang 2003), showed that *Endosamararacemosa* was sister to *Millettiajaponica* (*Wisteriopsisjaponica*) with BS (82%).

***Afgekia* Craib, Bull. Misc. Inform. Kew 1927(9): 376 (1927)**

This genus was named by Craib (1927) for Dr Arthur Francis George Kerr, the Irish botanist who collected widely in Siam [Thailand] from 1902–1932. Kerr’s Thai associate Anuwat collected the type specimen of *A.sericea* in Korat [now called Nakhon Ratchasima Province].

**Plate 2.**
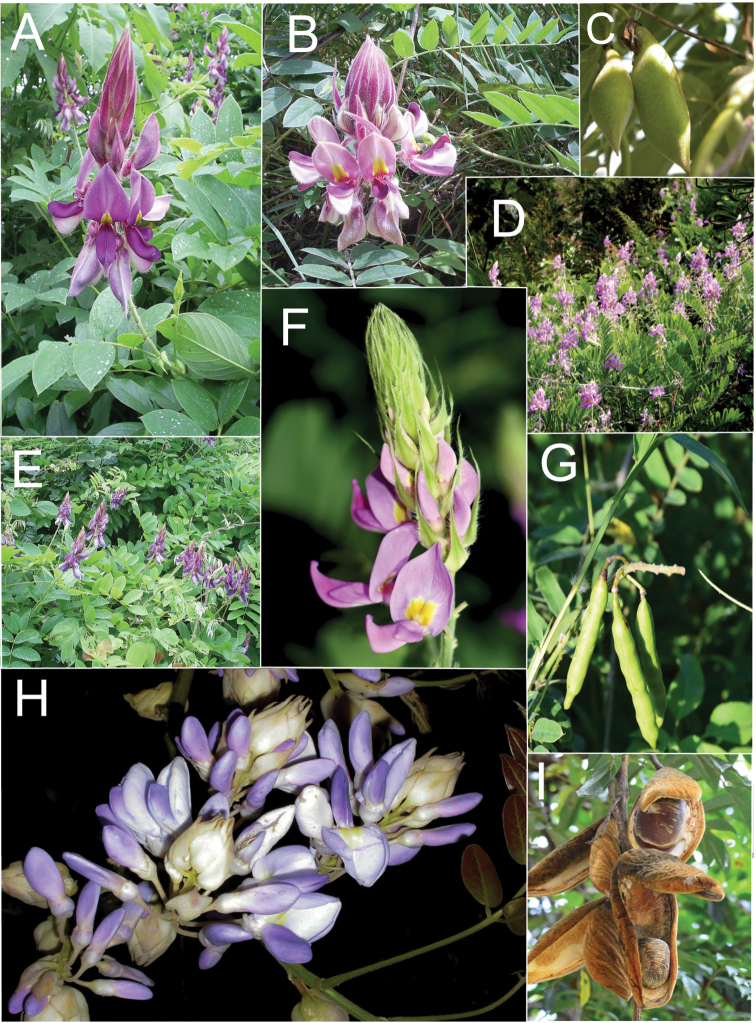
*Afgekia, Sarcodum* and *Padbruggea*. **A***Afgekiamahidoliae*, Thailand, Sai Yok distr. Kanchanaburi, *Y.Sirichamorn* s.n.. **B, C***Afgekiasericea* Thailand *S.Mattapha 1158***D***Sarcodumscandens* Vietnam, Quang Binh Prov. *Lôc & Quang P11554***E***Afgekiamahidiliae* Thailand, Sai Yok distr. Kanchanaburi *Y.Sirichamorn s.n.*. **F***Sarcodumscandens* Vietnam, Quang Binh Prov. *Lôc & Quang P11554***G***Sarcodumscandens* Laos, Sop Teuang, Bolikhamxai Prov. *S.Lanorsavanh 1299***H, I***Padbruggeafilipes* Thailand, Chiang Mai, *Y.Sirichamorn & S.Mattapha YSM2017-1*.

Previously, three species were recognised in the genus; *A.mahidoliae* (Plate 2A, E) from Kanchanaburi Province, west Thailand; *A.sericea* (Plate 2B) from north-east Thailand and *A.filipes* (Plate 2H, I). The latter is a much more robust climber from southern China, Laos, Myanmar, Thailand and Vietnam with panicles bearing robust and thickened inflorescence axes and fragrant pale to dark bluish-lilac flowers enclosed by caducous, broad, floral bracts. This species is fully discussed above and recombined in *Padbruggea*. The range of both *A.mahidoliae* and *A.sericea* is now known to extend into Laos and Vietnam in regions bordering Thailand (Lôc and Vidal 2001: 13, 14).

*Afgekia* (without *A.filipes*) has several distinguishing generic characters (Tables 4, 5): stipules 10–25 mm long (the longest by far in the *Callerya* group); racemes axillary or terminal (panicles in *A.filipes*); odourless flowers (fragrant in *A.filipes*); callosities in two pairs on the standard petal, (a unique character in the *Callerya* group); stamens with a distinctive ring of retrorse hairs on the filament immediately below the anthers and seeds with hila 15–30 mm long (Table 5).

In their analysis of *rbcL* sequence data, Hu and Chang (2003) found *Afgekiasericea* placed sister to the two *Wisteria* species sampled. Chloroplast *matK* sequence data of nine species of *Calleryas.l.*, *Afgekiasericea* and 11 samples from four taxa of *Wisteria* (Li et al. 2014) revealed, however, that *Afgekiasericea* was placed sister to a clade comprising *Calleryas.str.* This clade in turn was sister to another containing all the *Wisteria* samples and the two Australasian species *Calleryamegasperma* and *C.australis. Afgekiafilipes* was not included in this analysis. In the same paper, Li et al. (2014) also published their results from analyses of the nuclear DNA ITS spacer region. Those results showed that 14 samples of five taxa of *Wisteria* formed a discrete clade sister to one containing *Afgekiafilipes* and *Calleryadasyphylla* sister to *C.australis* and *C.megasperma*. *Afgekiasericea*, however, was placed in a separate clade (with poor support) sister to *Calleryaeurybotrya* and *C.reticulata*. In a majority consensus tree of the combined nuclear and chloroplast data *Afgekiasericea* is sister to *Calleryaeurybotrya* and *C.reticulata* while *Afgekiafilipes* is sister to *Calleryaaustralis* and *C.megasperma* (Li et al. 2014).

***Wisteria* Nutt., Gen. Amer. Pl. 2: 115 (1818)**

**Plate 3.**
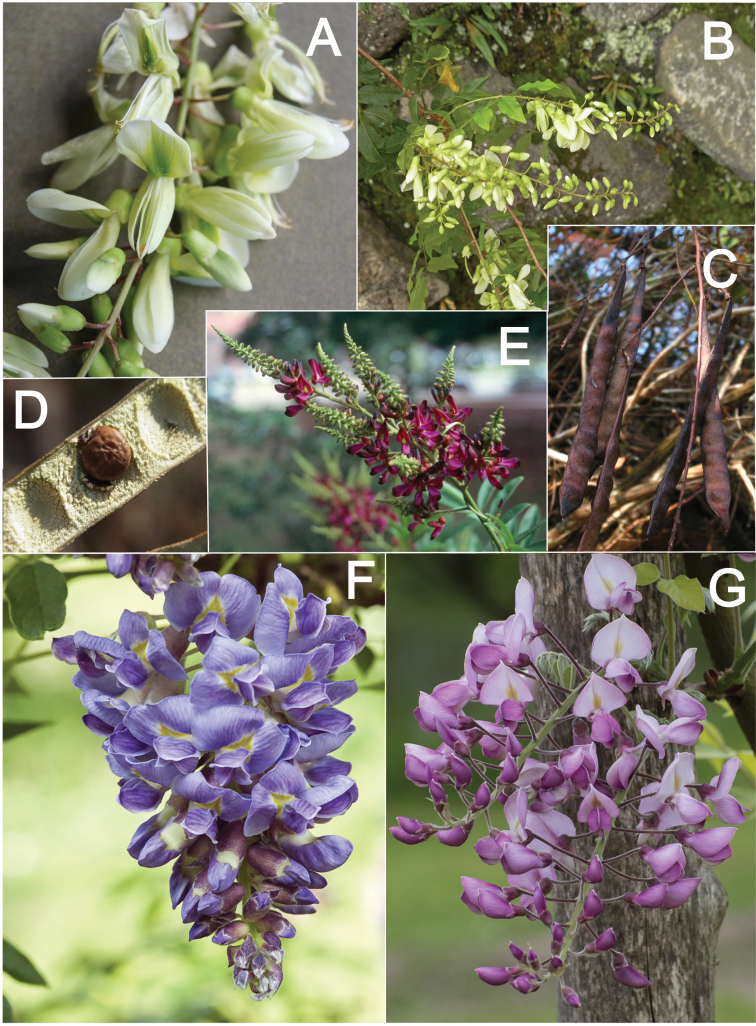
*Wisteriopsis* and *Wisteria*. **A***Wisteriopsisjaponica*, Cultivated, J.C.Raulston Arboretum, North Carolina 980008-17 **B***Wisteriopsisjaponica* Japan, Honshu near Kyoto *G.Lewis*, unvouchered **C, D***Wisteriopsisjaponica*, Cultivated, J.C.Raulston Arboretum, North Carolina 980008-17 **E***Wisteriopsisreticulata* Cultivated, *J.Compton s.n..* unvouchered **F***Wisteriafrutescens* Cultivated, *B.Schrire* unvouchered **G***Wisteriabrachybotrys* cultivated, *B.Schrire* unvouchered.

The genus *Wisteria* forms a distinct group of three species occurring in China, Japan and Korea and one, *Wisteriafrutescens*, in the eastern USA. The latter is the most distinct on account of its later summer (vs. spring) flowering; standard petals reflexing near the middle vs. at the base in the Asian species; callosities of the ridge (vs. papillate) type, broad wing petals which arch above the keel with the tips adherent to each other enclosing the keel and covering the staminal column prior to anthesis vs. adherent to the keel and not as above, and the straight, non-septate, externally smooth pods containing reniform seeds vs. subseptate, velutinous, gently torulose pods containing lenticular seeds (Table 4, Plate 3F).

The analyses using plastid *matK* and nuclear ITS sequence data discussed above under *Afgekia* (Hu et al. 2002, Li et al. 2014), are the only DNA based studies to have sampled all four species of the genus *Wisteria*. Hu et al. (2002) included nine species of *Callerya* and *Afgekiafilipes* while Li et al. (2014) included 15 species of the *Callerya* group. The other genera within the *Callerya* group, however, i.e. *Endosamara*, *Sarcodum* and *Wisteria/Millettiajaponica* were excluded in these analyses.

### *Callerya* group taxonomy 3: New genera within the *Callerya* group as delimited in this study

Our research has confirmed the uniqueness of other taxa within the *Callerya* group (Tables 1, 2). Schot noted the affinities between *Endosamararacemosa* and *Calleryakityana* in her revision of *Callerya* (Schot 1994: 25). Our results confirm that *Calleryakityana* with its sigmoid wing petals, among other unique autapomorphies, belongs in our new monospecific genus *Sigmoidala* (Fig. 3, Plate 1C–D). Our results have also revealed that two recently described Thai species, *Calleryachlorantha* and *C.tenasserimensis* (Sirichimorn et al. 2016) unequivocally share a suite of synapomorphies (Table 4) that segregate them from *Calleryas.str.* and they belong together in our new genus *Kanburia* (Plate 1E–F).

Dunn also recognised the distinctiveness of the three Australasian species *M.australis*, *M.megasperma* and *M.pilipes* which comprised his Sect. Austromillettia (Dunn 1912a: 140). Our results confirm Dunn’s recognition and that these all belong in our new genus *Austrocallerya* (Fig. 6 and distinguishing characters, Table 4).

Based on a sampling of the morphologically most distinctive and apparently isolated taxon *Calleryastrobilifera* Schot (which has not been sampled before in previous analyses), this species is placed here in the new genus *Serawaia*.

**Figure 4.**
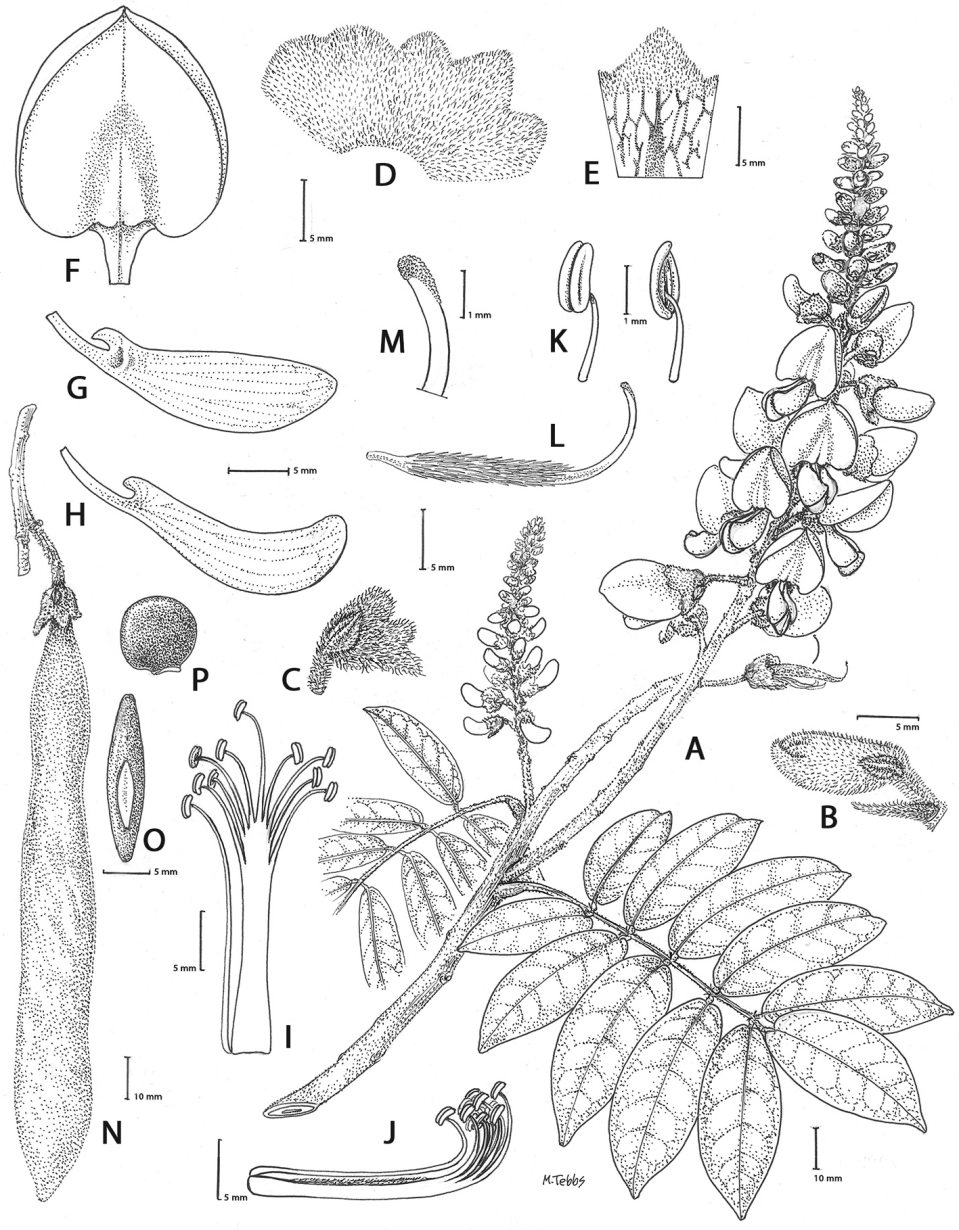
*Nanhaiaspeciosa* (Champ. ex Benth.) J.Compton & Schrire. **A** Habit **B** flower bud with bract and bracteole **C** calyx exterior and bracteole **D** detail of calyx exterior **E** detail of calyx interior **F** standard petal **G** wing petal **H** keel petal **I** staminal column **J** staminal column lateral view **K** stamen ventral and dorsal view **L** ovary and style **M** style and stigma **N** pod **O** seed lateral view **P** seed ventral view (all from *Shiu Ling Hu* 6091). Drawn by Margaret Tebbs.

Dunn (1912a: 135, 139) in his earlier revision of *Millettia*, placed *Millettiajaponica, Millettiachampionii, M.reticulata, Millettiaeurybotrya, Millettiaspeciosa* and *M.fordii* in his Sect. Eurybotreae Dunn. Schot (1994) transferred all these species (except *M.japonica*) into *Callerya* and they all form a strongly supported clade in our analyses underpinned by shared morphological synapomorphies (Fig. 1, Table 4). Two of these species *Calleryafordii* and *C.speciosa* share gibbosities and glabrous standards with the other species in Dunn’s Sect. Eurybotryeae but they differ in their densely pubescent ovaries and larger flowers. These two species are recognised here in the new genus *Nanhaia* (Fig. 4).

**Figure 5.**
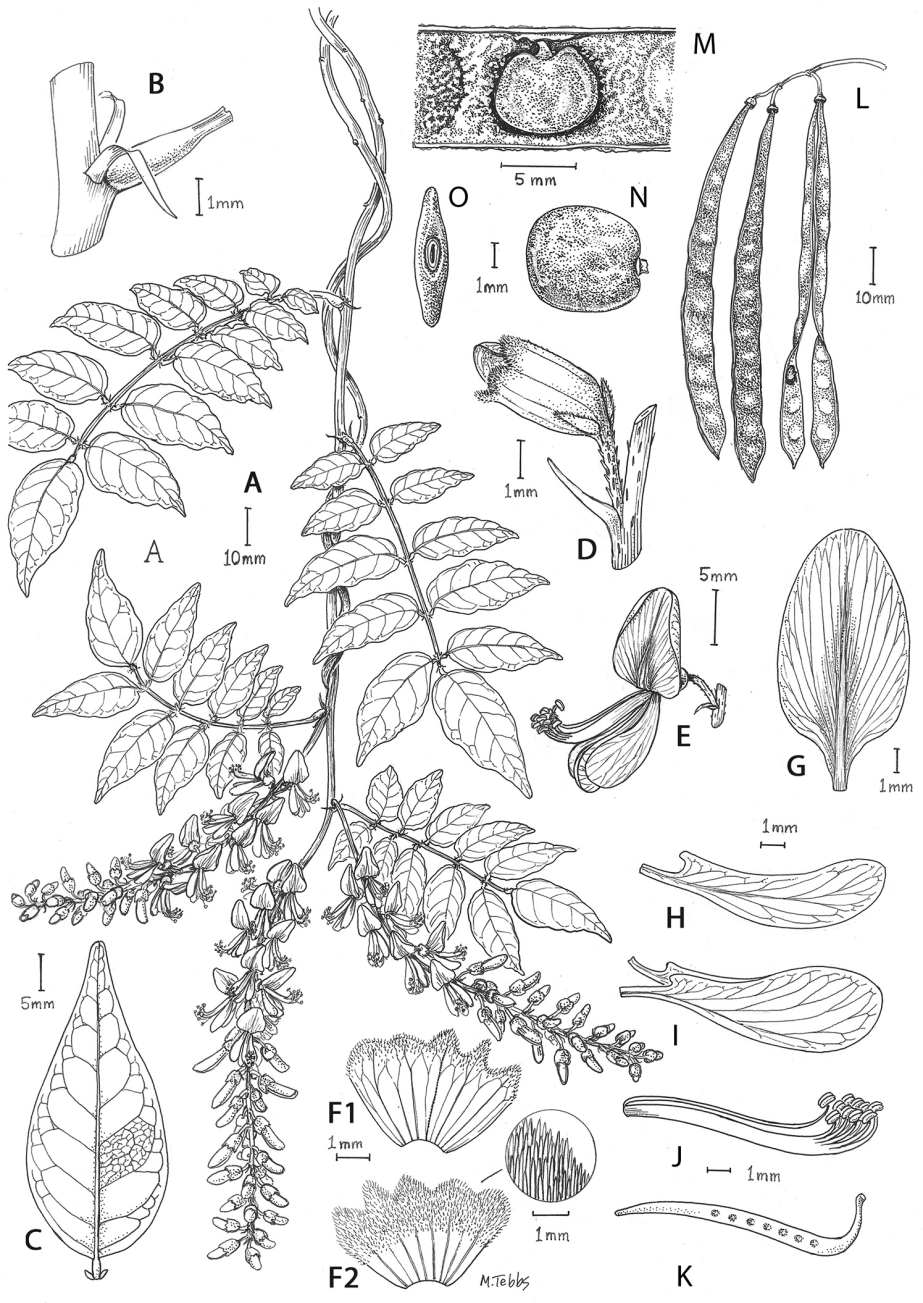
*Wisteriopsisjaponica* (Siebold & Zucc.) J.Compton & Schrire. **A** Habit **B** stipels **C** lower surface of leaflet **D** flower bud with bract and bracteoles **E** flower **F1** calyx outer surface **F2** calyx inner surface and detail of hairs **G** standard petal inner surface **H** wing petal **I** keel petal **J** staminal column **K** ovary and style **L** pods **M** pod interior and seed **N** ventral view of seed **O** lateral view of seed **A–C, E–K** from *Maximowicz s.n..* 1863 **D** from *Oldham* 386, **L–O** from *Togasi* MSM1, 1950. Drawn by Margaret Tebbs.

Geesink recognised the distinctive nature of *Millettiajaponica* [*Wisteriajaponica*] when he included it in a separate couplet in his key to the genera of Millettieae (Geesink 1984: 72). *Millettiajaponica* is the only species that has not formally been treated as belonging to the *Callerya* group although it has been included in various molecular analyses as either *Millettiajaponica* or *Wisteriajaponica* (Doyle et al. 1997, 2000; Kajita et al. 2001; Hu and Chang 2003). This is now the type species of our new genus *Wisteriopsis* which comprises the remaining species from Dunn’s Sect. Eurybotreae (Fig. 5, Plate 3A–E).

*Wisteriajaponica* Siebold & Zucc. has been recognised as distinct since the early days of European interest in Japanese botany (Fig. 5, Plate 3A–D). It was known to the physician Englebert Kaempfer who made a note of the species in the late 17^th^ century (Compton and Lack 2012). It was first validly described by Philipp von Siebold and Gerhard Zuccarini in their illustrated work *Flora Japonica* (Siebold and Zuccarini 1839: 88) based on material Siebold had collected near Nagasaki prior to 1829 (Compton and Thijsse 2013).

The American botanist Asa Gray recombined *Wisteriajaponica* into *Millettia* (Gray 1858: 386) with no additional descriptive information other than “found on Kiu-siu belongs to a more southern Asiatic type” and “this is truly a *Millettia*”. Dunn, however, in his revision of *Millettia* stated that there were several characters that allied the species with *Wisteria*, notably the deciduous, pinnate leaves and presence of what he considered to be large paniculate inflorescences of spreading to pendulous axillary racemes (Dunn 1912a: 153). Dunn also noted that there were other characters that separated the species from *Wisteria*, the occurrence of truly paniculate inflorescences, persistent bracts, and he asserted that the stamens of *M.japonica* were monadelphous (Dunn 1912a: 153). This taxon has also been accepted as belonging in *Millettia* in floristic works (Ohwi 1984) and in the horticultural literature (Valder 1995) and was recently maintained in *Millettia* in a genetic marker study (Kim et al. 2013).

Geesink (1984) placed *Millettiajaponica* close to his south-east Asian monotypic genus *Endosamararacemosa* in a couplet in his key to Tribe Millettieae based on their sharing paniculate inflorescences and what he perceived to have been an absence of callosities on the standard petal (see Table 4). Geesink also recognised that these two differed from each other by the uniquely compartmented and winged fruits in *Endosamara* and the presence of persistent bracteoles in *M.japonica*. He also linked *M.japonica* to his descriptions of *Wisteria*, *Callerya* and *Sarcodum* (Geesink 1984: 72, 93, 122). He considered *M.japonica* to be closest to, or included within, *Callerya* and specifically noted “the wings free from the keel in *Millettiajaponica*” (Geesink 1984: 83), a character that he also noted for *Wisteria* (Geesink 1984: 121). In addition he recognised similarities between *M.japonica* and *Sarcodum* (Geesink 1984: 117) stating:

*Sarcodum resembles Millettiajaponica (which I consider to belong to Callerya) in its habit, flower characters, and in the fleshy exocarp, but in M.japonica the pod is flat and not so convex around the seeds and this species has a “true” panicle*.

Schot (1994) in her revision of the genus *Callerya*, excluded Millettia (Wisteria) japonica on the basis that she believed the species had slender axillary racemes rather than panicles with thickened axes and stated that she considered the species to be closest to *Calleryareticulata* (Schot 1994: 5). Moreover, Schot quoted Dunn (1912a: 153) in assuming that the stamens were monadelphous. This was clearly an oversight in both cases as both *Callerya* (*sensu*Schot 1994) and *Millettiajaponica* have diadelphous stamens. Schot opined that the species “lacks the facies of a ‘true’ *Callerya*” but without further comment. Our results and observations have revealed that *Calleryareticulata* is indeed closely related to *Wisteriopsisjaponica* and also belongs in *Wisteriopsis* (Plate 3E). In addition, our studies support the inclusion of *Calleryachampionii, C.eurybotrya* and *C.kiangsiensis* within *Wisteriopsis*.

Recent molecular phylogenies that included *Wisteriopsisjaponica* (usually as *Millettiajaponica*) in their analyses all used data from the chloroplast gene *rbcL* (Doyle et al. 1997, 2000; Kajita et al. 2001; Hu and Chang 2003). Doyle et al. (1997) found that a single species of *Wisteria* (*Wisteria* sp.) and *Millettiajaponica* were sister to each other. Doyle et al. (2000) provided data in a larger dataset combined with morphology and found that two samples of *Wisteria*, one *Afgekia* and *Millettiajaponica* formed an unresolved clade separate from the rest of *Millettia*. Results from a parsimony analysis by Kajita et al. (2001) which included two species of *Wisteria* (*W.sinensis* and *W.* sp.), *Afgekiasericea* and *Millettiajaponica* found that in their strict consensus tree, the two *Wisteria* samples, *Millettiajaponica* and *Afgekiasericea* were unresolved in a separate (but unnamed) IRLC. Hu and Chang (2003) placed *Millettiajaponica* sister to *Endosamararacemosa* but these were unresolved with respect to two sister samples of *Wisteria*, *W.sinensis* and *W.* sp.

*Wisteriopsisjaponica* (sampled as *Wisteriajaponica*) has also been found to possess a unique terminal N-Acetylgalactosamine leguminous lectin which has been recognised to be useful as a probe for human lung squamous cell carcinoma (Soga et al. 2013). The molecular weight of the lectin in *Wisteriopsisjaponica* which does not bind to galactose, is different to the molecular weights of the lectins of both *Wisteriafloribunda* and *W.brachybotrys* and has a different sugar-binding specificity to the lectins of *W.floribunda*, *W.brachybotrys* and *W.sinensis* which all bind to galactose (Soga et al. 2013).

### Integrating our results: the *Callerya* group to Tribe Wisterieae

In the most recent family-wide phylogenies of Leguminosae (Wink 2013; LPWG 2013, 2017), the IRLC is strongly supported, with the *Callerya* group and *Glycyrrhiza* placed in equivocal positions relative to the Temperate Tribe block). In the Maximum Likelihood (ML) tree of the most comprehensive phylogeny to date (LPWG 2017), *Glycyrrhiza* is sister to *Schefflerodendron* (outgroup used to root our analyses) and these are sister to a clade comprising the *Callerya* group + the Temperate Tribe block. In their Bayesian Inference (BI) analysis (LPWG 2017), the *Callerya* group and *Glycyrrhiza* are unresolved in a polytomy along with the Temperate Tribe block. *Adinobotrys* was not included in these analyses. In our combined analyses (Fig. 1, Suppl. material 1: Figs S1–S6, Table 3), *Adinobotrys* is placed without support as part of a grade sister to *Glycyrrhiza* + the rest of the *Callerya* group and then *Glycyrrhiza*, again without support, is sister to the remainder of the *Callerya* group. In the plastid analyses (Table 3) the positions of *Adinobotrys* and *Glycyrrhiza* are switched about in the grade and in the BI analysis, *Adinobotrys* + *Glycyrrhiza* are sister to the rest of the *Callerya* group, all without support. In the ITS analyses, again without support, *Glycyrrhiza* is sister to the entire IRLC with *Adinobotrys* placed sister to the remainder of the IRLC excluding the rest of the *Callerya* group (Table 3). The equivocal positions of *Adinobotrys*, the residual *Callerya* group and *Glycyrrhiza*, in all recent phylogenies, points to them being relatively isolated elements compared to the Temperate Tribe block and an understanding of the relationships between them needs further research.

As discussed earlier, Jansen et al. (2008) noted that within the IRLC, the *rps*12 intron was uniquely present in the *Callerya* group but absent in all other IRLC taxa sampled, including *Glycyrrhiza*. Genera in the *Callerya* group, that they confirmed as having the *rps*12 intron, included species of *Adinobotrys, Endosamara, Wisteriopsis, Afgekia, Padbruggea, Austrocallerya* and *Wisteria*. Although *Adinobotrys* is supported as belonging within the *Callerya* group by Jansen et al. (2008) – and that superficially *A.atropurpureus* and *A.vastus* are very similar to other tropical genera within the *Callerya* group – we have decided to recircumscribe our emended Tribe Wisterieae to include the crown node of clades A to E (Fig. 1, Suppl. material 1: Figs S1–S6, Tables 3, 4). This node is fully supported in both the combined and plastid analyses and in the ITS BI analysis, although only weakly so in the ITS ML analysis (Fig. 1, Table 3) and is diagnosed by the significant morphological synapomorphy of the lianescent habit. *Adinobotrys* may well belong within Tribe Wisterieae based on the evidence of Jansen et al. (2008), but on the basis of the consistent lack of support for its inclusion in our analyses, it is excluded here. Hu et al. (2000) also faced this quandary in that while the Phytochrome gene data (Lavin et al. 1998) and pollen data (Zhu 1994) supported linking *Calleryaatropurpurea* with the *Callerya* group, their ITS and *trnK*/*matK* evidence suggested that *Calleryaatropurpurea* was a genetic outlier with respect to *Callerya*. Nevertheless, *Adinobotrys* is treated in full in our taxonomic treatment since it is clearly a disparate element within *Calleryas.l.* (*sensu*Schot 1994) and thus, with BS (100%) support, requires to be reinstated as a separate genus. The tree habit in *Adinobotrys* compared to lianas and scandent shrubs found without exception in Tribe *Wisterieae* serves to segregate this genus morphologically. Tribe Wisterieae together with *Adinobotrys* and *Glycyrrhiza* and the Temperate Tribe block thus represent the four main clades of the IRLC.

Tribe Wisterieae comprises a grade of three major clades (Fig. 1, Suppl. material 1: Figs S1–S6): the first branching Clade A (*Sarcodum* to *Sigmoidala*), sister to Clades B to E (*Nanhaia* to *Wisteria*) and then, Clade B (*Nanhaia* – *Wisteriopsis*) sister to Clades C–E (*Callerya* – *Wisteria*). In Clade A, the small genus *Sarcodum* and the monotypic *Endosamara* and *Sigmoidala* are all morphologically very distinct from each other. *Sarcodum* has botuliform, somewhat fleshy, pods with leaves comprising the smallest and usually most numerous leaflets in the tribe, *Endosamara* has unique winged and lomented seeds and *Sigmoidala* has distinctive ‘S’ shaped wing petals. *Sigmoidala* (as *Callerya*) *kityana* was treated as part of *Calleryas.l.* by Schot (1994) and is described here as a new genus. All three genera are fully supported at the generic level in the combined, plastid and ITS analyses as are all three together within Clade A except for the ITS ML and BI analyses where, with no support, the long-branched *Endosamara* appears attracted to other long branched genera within Clade C (Fig. 1, Table 3). The three genera together with *Wisteriopsis* (in Clade B) share glabrous ovaries as a unique character in the tribe; *Sarcodum* and *Endosamara* both share glabrous standards whereas the back of the standard in *Sigmoidala* is rufous pubescent and *Sarcodum* is the only one of the three with erect leafy racemes as opposed to the terminal panicles of the other two genera (Table 4). The genus *Sarcodum* has not been included in previous molecular phylogenies and is shown here to be fully supported as sister in the combined analyses to *Endosamara* and *Sigmoidala*.

Clade B (Fig. 1) comprises two new genera, *Nanhaia* and *Wisteriopsis* that both produce prominent gibbosities near the point of attachment of the leaf pulvinus to the stem (a character only shared otherwise by *Serawaia* in Clade C). Another synapomorphy shared by *Nanhaia* and *Wisteriopsis* is the presence of an annulus of hairs surrounding the calyx rim. Both genera also share glabrous standards with *Endosamara* and *Sarcodum*. *Nanhaia* and *Wisteriopsis* comprise species included within *Calleryas.l.* by Schot (1994), but a number of additional species in *Wisteriopsis* came to light as unsuspected taxa associated previously with *Millettia*, *Wisteria* and Chinese *Calleryas.l.* Both *Nanhaia* and *Wisteriopsis* are fully or very strongly supported as genera in all analyses except where *Nanhaia* is represented as a single accession (*Nanhaiaspeciosa*) in the ITS analyses (Fig. 1, Table 3) and both are fully to strongly supported together as Clade B. Moderate support values for the sister group relationship between Clade B and Clades C–E reflect the unstable position of Clade B relative to Clade C in the ITS analyses (Table 3).

Clade C (Fig. 1) comprises the genera *Calleryas.str.* (Clade C1), sister to a moderately to strongly supported alliance (in the combined analyses) of Clades C2 + C3 + C4 (Table 3), comprising *Whitfordiodendron, Serawaia, Kanburia* and *Afgekia* This grouping is poorly supported in the ITS analyses, however, and it breaks apart somewhat in the plastid analyses. Clade C as a whole is moderately (BS) to strongly (BPP) supported in the combined analyses but tends to break up and group with other genera in the plastid and ITS results (Table 3). The genera *Afgekia*, *Kanburia* and *Whitfordiodendron* are each fully supported, as is *Calleryas.str.* at the node above *C.bonatiana*. *Calleryas.str.*, including *C.bonatiana*, is well supported in the combined and plastid BI analyses (Table 3) but only poorly so in the ML analyses. The ITS results either split *Calleryas.str.*, placing *C.bonatiana* in a polytomy with the long-branched *Endosamara* (from Clade A2) in the ML analysis, or in an unsupported position sister to Clade B in the BI analysis. *Calleryabonatiana* shares the morphological synapomorphies of *Calleryas.str.*, e.g. wings shorter than the keel, and none of the synapomorphies of other Wisterieae genera so it is treated here as part of *Calleryas.str.* The genus *Whitfordiodendron* is reinstated at generic level from *Calleryas.l.* (Schot, 1994) and a new genus *Kanburia* is described from only recently collected material (Sirichamorn et al. 2016). Sister group relationships with *Kanburia* (Clade C3) are generally not supported, although in the ITS analyses the strongest (although still poor) support is with *Serawaia* (Table 3). In the combined BI analysis, however, there is strong support (0.95 BPP) for *Afgekia* being sister to *Kanburia*. The genus *Serawaia* is the only new genus described here that is based on a single accession since the DNA of all other material sampled was too degraded to be useful. It is a morphologically unique taxon in the Tribe Wisterieae with large cone-like strobilate inflorescences of bright yellow flowers with the persistent 15–18 × 8–12 mm bracts becoming indurate and coriaceous in fruit. No other taxon has this combination of characters and while it is placed sister to *Whitfordiodendron* with no support in the combined analyses and sister to *Kanburia* with poor support in the ITS analyses (Fig. 1, Table 3), it remains strongly supported within Clade C (BS 87%, BPP 0.98) in the combined analyses. At the same time *Serawaia* cannot be placed morphologically with any other genus in Clade C. Morphologically, the presence of gibbosities below the stipules and wing petals free of the keel are unique in Clade C but are characteristic of all or some taxa in Clade B. No support is found, however, for links between *Serawaia* and clade B in these analyses.

*Afgekia* comprises two very distinct species separated from the other genera in the clade by a long branch and a large number of synapomorphies, most notable of which are the racemes vs. panicles present in the rest of Clade C, long sericeous floral bracts and, uniquely within the tribe, the two pairs of callosities on the standard petals (Table 4). The seeds of *Afgekia* have long strap-shaped hila compared to the short elliptic hila in the other genera of Clade C. *Callerya* is distinguished by having wing petals much shorter than the keel, *Whitfordiodendron* by its densely sericeous keels (glabrous elsewhere in the tribe although pubescent to tomentose in *Afgekia*) and *Afgekia* and *Kanburia* both lack bracteoles (present in *Callerya*, *Serawaia* and *Whitfordiodendron*). *Afgekia*, *Callerya*, *Kanburia*, *Serawaia* and *Whitfordiodendron* all share densely pubescent or sericeous ovaries and backs to the standard petals. The pods of *Whitfordiodendron* are inflated, ovoid, rugose to ridged or ruminate (Fig. 2P), with 1– 2(–3) seeds becoming fused together when more than one in the pod. The pods in *Afgekia* are inflated with densely velutinous surfaces (vs. in *Callerya*, *Serawaia* and *Kanburia* pods are flattened to inflated, smooth, with (1–)2–6 seeds remaining separate in the pod (Fig. 2L).

The genera *Padbruggea* and *Austrocallerya* form a strongly supported Clade D (Table 3) in the combined, plastid BI and ITS analyses although support is weak in the plastid ML analysis. *Padbruggea* and *Austrocallerya* are also strongly supported as genera in all analyses. *Padbruggea* is reinstated in part from *Calleryas.l.* (Schot, 1994) and by the transfer of *P.filipes* from *Afgekia*. *Austrocallerya* is described as a new genus comprising the Australasian species in *Calleryas.l.* (Schot, 1994). The two genera share seeds with long, strap-shaped hila and more open, laxly flowered panicles as morphological synapomorphies and are segregated from each other based on standard callosity shape, fruit characters and geographic distribution. Clade D is sister to Clade E (comprising *Wisteria*) with strong support in all results (Table 3) except in the plastid ML analysis.

Four species of *Wisteria*, three in temperate east Asia (Clade E1) and one in North America (the only non Asian-Australasian species in the Wisterieae, Clade E2) comprise Clade E with full support in the combined, plastid and ITS analyses (Table 3). Clade E is distinguished from Clade D by its deciduous vs. evergreen habit, racemose vs. paniculate inflorescences, seeds up to 12 mm in size in compressed pods vs. seeds larger than 12 mm in inflated pods and seeds with short elliptic hila vs. long strap-shaped hila (Table 4). The deciduous habit is only found otherwise in *Wisteriopsisjaponica*.

Aberrant results in the ITS analyses compared to those derived from plastid data are thought likely to be the outcome of fewer representative taxa, probable long branch attraction in the placement of *Endosamara*, *Kanburia*, *Serawaia* and *Afgekia* and alignment problems with ITS making it difficult to ascertain true homology. The 77.1% pairwise percentage of identity across the ITS alignments vs. 92.5% for the overall plastid alignments may also be indicative of ITS being less informative than the plastid data in these analyses. It is also apparent that the two single accessions of *Calleryabonatiana* and *Serawaiastrobilifera* (both limited by a lack of research material) are the most labile in the phylogeny, thereby reducing the support values of their associated clades.

### Taxonomic treatment of Tribe Wisterieae

Thirteen genera within a much expanded Tribe Wisterieae are described here, encompassing five clades recovered in our phylogenetic analyses (Fig. 1). In addition, the genus *Adinobotrys* is fully described owing, in part, to its long association with the *Callerya* group but also because of its equivocal sister group relationships to tribe Wisterieae in these analyses. The following clades and included genera are listed below (Fig. 1):

Clade A) *Sarcodum, Endosamara, Sigmoidala*

Clade B) *Nanhaia, Wisteriopsis*

Clade C) *Callerya, Whitfordiodendron, Kanburia, Afgekia, Serawaia*

Clade D) *Padbruggea, Austrocallerya*

Clade E) *Wisteria*

### Emended diagnosis of Tribe Wisterieae

#### Tribe Wisterieae

Taxon classificationPlantaeFabalesFabaceae

Zhu, Cathaya 6: 115–124 (1994)

≡ Subtribe Wisteriinae Endl. Gen. Pl.: 1296 (1840) [as Subtribe Wisterieae].

##### Type.

*Wisteria* Nutt., Gen. Amer. Pl. 2: 115 (1818) nom. cons. ≡ *Glycinefrutescens* L., Sp. Pl. 1(2): 753 (1753).

##### Note.

The Tribe Wisterieae is distinguished by comprising woody lianas or sprawling scandent shrubs. All species have bracts that in the most part enclose immature buds at the apex of inflorescences and all bear either true panicles or true racemes as opposed to pseudopanicles and pseudoracemes. The tribe is further distinguished from Tribe Millettieae by all genera lacking one 25 kb long copy of the inverted repeat in the chloroplast genome.

### Morphological key to the genera in Tribe Wisterieae together with *Adinobotrys*

**Table:** 

1	Inflorescences of strobilate, axillary or terminal racemes or panicles with few side axes each terminated by a strobilate bud, floral bracts 8–12 mm wide, persistent, imbricate, becoming indurate, coriaceous in fruit; flower bright to golden yellow	*** Serawaia ***
–	Inflorescences never strobilate, floral bracts only as wide in *Wisteria* and *Padbruggea* where they are caducous; flowers of many colours except pure yellow	**2**
2	Inflorescences comprising terminal leafless panicles or a combination of racemes aggregated terminally in leafy panicles	**3**
–	Inflorescences comprising racemes only	**12**
3	Bracteoles absent	**4**
–	Bracteoles present	**6**
4	Seeds enclosed within lomented endocarp and dispersing as individual samaroid units at maturity; back of standard glabrous	*** Endosamara ***
–	Seeds not enclosed within papery endocarp or dispersing as individual units; back of standard pubescent	**5**
5	Flowers 1.6–2 cm long; wing petals sigmoid, longer than keel; reflexed after anthesis; back of standard densely pubescent with ferrugineous hairs	*** Sigmoidala ***
–	Flowers 1–1.5 cm long; wings ± equalling the keel, straight and not reflexed after anthesis; back of standard sericeous with golden-brown hairs	*** Kanburia ***
6	Trees; back of standard glabrous	*** Adinobotrys ***
–	Lianas; back of standard glabrous, puberulent or sericeous	**7**
7	Back of standard glabrous; prominent gibbosities present on stem below stipules	**8**
–	Back of standard pubescent; gibbosities absent on stem below stipules	**9**
8	Ovary glabrous; flowers 0.7–1.6 cm long; stamens visible between wings and keel at anthesis	*** Wisteriopsis ***
–	Ovary puberulent or sericeous; flowers (1.6 –)1.7–3.2 cm long; stamens enclosed within wings and keel at anthesis	*** Nanhaia ***
9	Back of standard densely sericeous; pedicels 0.5–6(– 8) mm long; seed hilum rounded to elliptic, 1–5 mm long	**10**
–	Back of standard sparsely pubescent; pedicels (3 –)8–25 mm long; seed hilum strap-shaped, 10–40 mm long	**11**
10	Wings shorter than keel; keel glabrous	*** Callerya ***
–	Wings ± equalling keel in length; keel densely sericeous especially along lower margin	*** Whitfordiodendron ***
11	Callosities on standards arched on either side of midline; pods cylindrical, torulose; surfaces finely striated or smooth; seeds slightly longer than wide or ± equal	*** Austrocallerya ***
–	Callosities on standard forming straight ridges parallel to the midline, or papillate; pods oblong-ovate; surfaces coarsely ridged; seeds ± twice as long as wide	*** Padbruggea ***
12	Leaves with (9 –)17–45 leaflets, these oblong-elliptic, apex obtuse, 6–12 mm wide; standard with boss callosities; pods with 4–10 seeds, botuliform, septate with fleshy exocarp, seeds 3–6 mm thick; seed hilum elliptic, 2–2.5 mm long	*** Sarcodum ***
–	Leaves with 9–19 leaflets, these ovate to lanceolate, apex acute, (12 –)14–40 mm wide; standard with papillate or ridge callosities; pods subseptate with coriaceous exocarp	**13**
13	Plants evergreen; racemes ascending to erect; stipules 10–25 mm long; standard with two pairs of callosities, a basal papillate pair and above that a corniculate pair; pods ellipsoid with 2 seeds, seeds 8–13 mm thick, seed hilum strap-shaped, 15–30 mm long	*** Afgekia ***
–	Plants deciduous; racemes descending to pendent; stipules 2–6 mm long; standard with a single pair of papillate or ridge callosities; pods linear with 2–5 seeds, seeds 2–6 mm thick, seed hilum elliptic 1–4 mm long	*** Wisteria ***

#### 1. Adinobotrys

Taxon classificationPlantaeFabalesFabaceae

Dunn, Bull. Misc. Inform. 1911: 194 (1911), emend. nov. J.Compton & Schrire

≡ MillettiaSect.Nothomillettia Miq., Fl. Ned. Ind., Eerste Bijv. 2: 301 (1861) ≡ Millettiasubgen.Nothomillettia (Miq.) Kurz, J. Asiat. Soc. Bengal, Pt. 2, Nat. Hist. 45(2): 273 (1876).

##### Diagnosis.

*Adinobotrys* comprises two species of evergreen trees (vs. lianes in Tribe Wisterieae). The bracteoles are persistent (caducous in *Calleryas.str.*), the calyx is oblique in both species, the standard is glabrous (sericeous in *Calleryas.str.*) and the wing petals are ± equal to the keel in length (vs. much shorter than the keel in *Calleryas.str.*).

##### Type species.

*Adinobotrysatropurpureus* (Wall.) Dunn ≡ *Pongamiaatropurpurea* Wall.

##### Genus description.

Large spreading evergreen trees to 20 m or more in height. *Stems* green when young, terete, finely brown pubescent becoming brown and glabrous with age. *Leaves* with 5–9 (– 11) leaflets, evergreen, coriaceous and nitent when mature, imparipinnate, rachis 11–33 cm long. *Stipules* 2–4 mm long, deltoid, persistent. *Stipels* absent. *Leaflets* 5–21 × 2–11 cm, ovate, elliptic or obovate, glabrous above and below, upper surface nited, apex acute to acuminate, margins entire, base obtuse or cordate. *Inflorescence* a robust many-flowered erect terminal panicle 10–40 cm long, peduncle sparsely hairy to tomentose. *Flowers* 14–20 mm long, emerging from February to May (in *A.atropurpureus*) and May to November (in *A.vastus*). *Floral bracts* 2–4 mm long, persistent (caducous in *A.vastus*), ovate. *Bracteoles* 1–2 mm long, at base of calyx tube, persistent, ovate. *Pedicels* 2–6 mm long, densely pubescent. *Calyx* narrowly campanulate, oblique, green, tube 4–6 × 6 mm, puberulent externally, five lobed, lobes unequal 0.5 mm long, acute or obtuse. *Standard* 11–20 × 13–20 mm broadly ovate, inner surface pink, dark reddish-purple, rarely white, nectar guide yellow, back of standard glabrous, apex acute, callosities of boss type. *Wing petals* 12–19 × 5–8 mm, glabrous, ± equal or longer than keel in length, each broadly semi-pandurate with basal claws 3–5 mm long. *Keel petals* 12–18 × 9 mm, glabrous, apex acute to rounded. *Stamens* diadelphous, nine fused together, the vexillary one free, all curved upwards at apex. *Ovary* sparsely to densely hairy, style glabrous, 5–6 mm long curved upwards at apex, *stigma* punctate. *Pods* 7–25 × 3–6 cm, inflated or flat (*A.vastus*), irregularly ovate to oblong or narrowly elliptic, dehiscent, surface glabrous, finely rugose, subseptate. *Seeds* 1–4, irregularly ovoid to oblong or flattened orbicular, sometimes laterally compressed inside the pod, 15–38 × 20–35 × 3–26 mm, hilum 2–3 × 2 mm, ovate-elliptic or circular.

##### Etymology.

adino - botrys = congested - bunch (Gk) referring to the congested inflorescence.

#### Key to species of *Adinobotrys*

**Table:** 

1	Floral bracts 1.5–2 mm long; flowers 17–20 mm long; pod inflated, elliptic to obovate; seeds 1–2, ovoid, 30–38 × 33–35 × 20–26 mm	*** A. atropurpureus ***
–	Floral bracts 3–4 mm long; flowers 14–15 mm long; pod not inflated, flattened, narrowly elliptic to narrowly obovate; seeds 2–4, flattened lenticular, 15–20 × 25–30 × 3–5 mm	*** A. vastus ***

##### Adinobotrys atropurpureus

Taxon classificationPlantaeFabalesFabaceae

(Wall.) Dunn, Bull. Misc. Inform. 1911(4): 194 (1911)

≡ Pongamiaatropurpurea Wall. Pl. As. Rar. 1(4): 70 t. 78 (1830). Type: Myanmar. “Martaban [Mottama] ad Amherst [Kyaikkami] 15 July 1827”, *Wallich* Cat. No. *5910*, K000881026 (K, holo.!); (BO, iso.); (CAL, iso. x 2); BM000997335 (BM, iso!), P02141756 (P, iso!) ≡ Millettiaatropurpurea (Wall.) Benth. Pl. Jungh. [Miquel] 2: 249 (1852) ≡ Phaseoloidesatropurpurea (Wall.) Kuntze, Revis. Gen. Pl. 1: 201 (1891) ≡ Whitfordiodendronatropurpureum (Wall.) Dunn, Bull. Misc. Inform. Kew 1912(8): 364 (1912) ≡ Calleryaatropurpurea (Wall.) Schot, Blumea 39(1–2): 15 (1994).
= Millettiapaniculata Miq., Fl. Ned. Ind., Eerste Bijv. 2: 301 (1861). Type: Indonesia, Sumatra “Sumatra orient. in prov. Palembang prope Kebur Lahat (T.)” 3675 H.B. Leguminosae, Masiboengan, Hortus Botanicus 023149 Utrecht, [Johannes Elias] Teijsmann s.n.., U0003669 (U, holo.!).
= Padbruggeapubescens Craib, Bull. Misc. Inform. Kew 1927(2): 61 (1927) Type: Thailand, Prov. Nakawn Panom [Nakhon Phanom], Ta Uten, elev. 1200 m, 15 February 1924, tree, fls pink. Ki Mo., A.F.G.Kerr 8457, K000881016 (K, holo.!); (ABD, iso.); BM000997332 (BM, iso.!); E00275433 (E, iso.!) ≡ Whitfordiodendronpubescens (Craib) Burkill, Bull. Misc. Inform. Kew 1935(5): 319 (1935) ≡ Calleryaatropurpurea(Wall.)Schotvar.pubescens (Craib) P.K.Lôc, Bot. Zhurn. (Moscow & Leningrad) 81(10): 98 (1996).

###### Illustrations.

Lôc and Vidal in Fl. Cambodge, Laos & Vietnam 30: 34, t. 8 [9–11] (2001). https://singapore.biodiversity.online (in Home Page enter *Calleryaatropurpurea*)

###### Distribution.

Cambodia; India; Indonesia (Java, Sumatra); Laos, Malaysia (Peninsula); Myanmar; Thailand and Vietnam.

###### Habitat..

component of evergreen forests from sea level to 1200 m.

##### Adinobotrys vastus

Taxon classificationPlantaeFabalesFabaceae

(Kosterm.) J.Compton & Schrire comb. nov.

urn:lsid:ipni.org:names:77198982-1

≡ Millettiavasta Kosterm., Reinwardtia 5: 349 (1960). Type: Indonesia, Kalimantan [Borneo], Belajan River near Muara Lempong, June 1956, [André Joseph Guillaume Henri] *Kostermans 12516A*, BO-1249898 (BO, holo.!); BM000997327 (BM, iso.!); L0018805 (L, iso.!); K000880991 (K, iso.!); P03081895 (P, iso.!) ≡ Calleryavasta (Kosterm.) Schot, Blumea 39(1–2): 36 (1994).

###### Distribution.

Borneo: Brunei; Indonesia (Kalimantan); Malaysia (Sabah, Sarawak).

###### Habitat.

Component tree in woods and forests from sea level to 250 m.

### Clade A – *Sarcodum, Endosamara and Sigmoidala*

(Fig. 1; Suppl. material 1: Figs S1–S6)

#### 2. Sarcodum

Taxon classificationPlantaeFabalesFabaceae

Lour., Fl. Cochinch. 2: 462 (1790)

##### Diagnosis.

The three species of *Sarcodum* are most closely allied to *Endosamara* and *Sigmoidala* but the genus is easily distinguished from the other two by the presence of bracteoles subtending the calyces (absent in *Endosamara* and *Sigmoidala*) and the smaller leaflets (0.3–2(–2.5) cm wide in *Sarcodum* vs. (2–)2.5–7 cm wide in *Endosamara* and *Sigmoidala*). *Sarcodum*, moreover, has leafy racemose inflorescences as opposed to the robust, erect panicles found in *Endosamara* and *Sigmoidala*. The back of the standard in *Sarcodum* and *Endosamara* is glabrous while that of *Sigmoidala* is densely pubescent. *Sarcodum* has fleshy botuliform pods that become woody on drying with oblate seeds borne in septate chambers while the seeds of *Sigmoidala* are flattened, ellipsoid and those of *Endosamara* are oblong, surrounded by a papery endocarp. The most widespread species *S.scandens* has leaves with between 17 and 45 narrowly elliptic leaflets – the most numerous in the tribe – each terminating in a short mucro.

##### Type species.

*Sarcodumscandens* Lour.

##### Genus description.

Three species of scandent twining vines scrambling over shrubs reaching 5–10 m. *Stems* grey-green when young, terete, densely pubescent, mature stems dark green becoming rusty brown, glabrous. *Leaves* with 9–45 leaflets, evergreen, often spotted with tannin deposits, sericeous when mature, imparipinnate, rachis 6–19 cm long. *Stipules* 3–12 mm long, linear-lanceolate, persistent. *Stipels* 3–6 mm long, linear, persistent. *Leaflets* 0.8–4.5 × 0.3–2.5 cm, elliptic, grey-green sericeous above and densely white sericeous below, apex rounded, mucronate, mucro c. 1.5–2 mm (retuse in *S.bicolor*), margins entire, base rounded. *Inflorescence* of erect leafy axillary and terminal racemes 3–12 cm long, peduncle densely silvery sericeous. *Flowers* 6–19 mm long, emerging from November – April. *Floral bracts* 6–20 mm long, caducous, densely pubescent, narrowly deltoid or ovate-deltoid apex acute or long acuminate. *Bracteoles* at base of calyx 2–7 mm long. *Pedicels* 4–12 mm long, densely pubescent. *Calyx* 3 × 5 mm, broadly campanulate, green or pink, sericeous externally, five lobed, upper 2 lobes ± connate, lower 3 lobes 1–3 mm long, acute. *Standard* 10–13 × 6–8 mm, ovate, inner surface glabrous, pink or pinkish-lilac, nectar guide broad, dark yellow, back of standard glabrous, apex acute. Callosities of boss type. *Wing petals* 8–13 × 3 mm, glabrous, much shorter than or subequal to the keel, each narrowly semi-pandurate, slightly curved upwards at the apex; free from the keel, basal claws 1–4 mm long. *Keel petals* 13 × 4 mm, glabrous, united into a falcate, navicular cup, apex acute and somewhat reflexed. *Stamens* diadelphous, nine fused together, the vexillary one free, all curved upwards at apex. *Ovary* glabrous, style glabrous, 3–4 mm long, curved upwards at apex, *stigma* punctate. *Pods* 3.5–5 × 0.7–1.2 cm, green, botuliform, dehiscent, gently torulose, surface glabrous, black and hard when dry, internally septate. *Seeds* 4–10, ellipsoid or oblong, 5–7.5 × 3.5–5 × 2.5–4.5 mm, oblong, rounded at each end, hilum central, broadly elliptic 2–2.5 × 1 mm.

##### Distribution.

China; Indonesia; Laos; Philippines; Solomon Islands; Vietnam.

##### Etymology.

sarcos = Gk fleshy.

##### Habitat.

All three species are climbing and scrambling vines growing in low thicket from sea level to 300 m.

#### Key to species of *Sarcodum*

**Table:** 

1	Leaves with 9–15 leaflets, leaflet apices rounded or retuse; floral bracts ovate-deltoid 10 × 4 mm	*** S. bicolor ***
–	Leaves with 17–45 leaflets; leaflet apices mucronate; floral bracst narrowly deltoid	**2**
2	Floral bracts 6 × 0.5 mm	*** S. solomonensis ***
–	Floral bracts 16–30 × 3 mm	*** S. scandens ***

##### Sarcodum scandens

Taxon classificationPlantaeFabalesFabaceae

Lour., Fl. Cochinch. 2: 462 (1790)

= ClianthusbinnendyckianusKurz, J. Asiat. Soc. Bengal Pt. 2 Nat. Hist. 40(1): 51 (1871). Type: “Moluccos [Maluku], Ceram [Seram], Cult. in Hort. Bogor ab Binnendyck”, S.Binnendijk s.n.., (BO, holo., not seen); K000117839 (K, iso.!).

###### Type.

Vietnam. “In sylvis Cochinchinae, G 151 Sarkinum = Cay muong deei = Sarcodum p. 462 2-delph” *J. de Loureiro* or local collector, BM001209557 (BM, holo.!) ≡ *Clianthusscandens* (Lour.) Merr., J. Bot. 66: 265 (1928)

###### Illustrations.

Lôc and Vidal in Fl. Cambodge, Laos & Vietnam 30: 7, t. 1 (2001); Clark in Kew Bull. 63(1): 156 (2008); Sun and Pedley FOC Illustrations 10: 175 fig. 202 [1–9] (2010). Plate 2D, Plate 2F–G.

###### Distribution.

China (Hainan); Indonesia (Seram, Sulawesi); Laos; Philippines; Vietnam.

##### Sarcodum bicolor

Taxon classificationPlantaeFabalesFabaceae

Adema, Blumea 44: 407 (1999)

###### Type.

Indonesia, Sumba, Nusa Tengarra [Lesser Sunda Islands], “1925, Soemba”, [L.] *Iboet* 385, L0064653 (L, holo.!); A00104485 (A, iso.!); K000117840 (K, iso.!); (SING, iso.)

###### Illustration.

Clark in Kew Bull. 63(1): 159 (2008).

###### Distribution.

Indonesia (Sumba Island).

##### Sarcodum solomonensis

Taxon classificationPlantaeFabalesFabaceae

R.Clark, Kew Bull. 63(1): 155, t. 1 (2008)

###### Type.

Solomon Islands, Gizo Island ridge top 250 ft. asl. 28 April 1970, *R.Mauriasi & collectors* BSIP 18096, K000556150 (K, holo.!); L0418332 (L, iso.!).

###### Distribution.

Solomon Islands (Gizo).

##### 3. Endosamara

Taxon classificationPlantaeFabalesFabaceae

R.Geesink, Leiden Bot. Ser. 8: 93 (1984)

≡ MillettiaSect.Bracteatae Dunn, J. Linn. Soc., Bot. 41: 135 (1912a)

###### Diagnosis.

The monospecific *Endosamararacemosa* was recognised at generic level by Geesink (1984: 93) principally by its unique fruits with their seeds forming segregating loments encased in endocarp each with a flat wing, a unique feature within the tribe. *Endosamara* has stipules 6–12 mm long (vs. 3–7 mm in *Sigmoidala* and 1–6 mm in *Calleryas.str.*), lacks floral bracteoles, has glabrous ovaries and glabrous standard petals (vs. back of standard pubescent in *Sigmoidala*).

###### Type species.

*Endosamararacemosa* (Roxb.) Geesink ≡ *Robiniaracemosa* Roxb.

###### Genus description..

robust, twining woody vine. *Stems* green when young, terete, pubescent, mature stems pale brown, glabrous. *Leaves* with 7–13 leaflets, evergreen, coriaceous when mature, imparipinnate, rachis 10–24 cm long. *Stipules* 6–12 mm long, narrowly lanceolate, persistent, becoming woody and spinose on old branches. *Stipels* 3–6 mm long, linear, persistent. *Leaflets* 5–13 × 2–7 cm, oblong-obovate, elliptic, glabrous above, tomentose below becoming glabrous, apex acute, margins entire, base obtuse to cuneate. *Inflorescence* a robust many-flowered terminal panicle 20–50 cm long, peduncle densely silvery-brown hairy. *Flowers* 12–16 mm long, emerging from March – June. *Floral bracts* 6–15 mm long, caducous, densely pubescent, linear-lanceolate. *Bracteoles* absent. *Pedicels* 3–6 mm long, densely pubescent. *Calyx* 3 × 6 mm campanulate, green, densely puberulent externally, five lobed, lobes ± equal, 1–3 mm long, broadly acute, obtuse or subtruncate, becoming more fleshy and rounded at maturity. *Standard* 10–15 × 12–15 mm, suborbicular or broadly ovate, inner surface pale to dark pink, pinkish purple, rarely white, nectar guide greenish yellow, back of standard glabrous, apex acute or emarginate. Callosities of boss type. *Wing petals* 12–13 × 3–5 mm, glabrous, slightly longer than keel, each narrowly semi-pandurate, slightly curved upwards at the apex with basal claws 3 mm long. *Keel petals* 10–12 × 4–6 mm, glabrous, united into a falcate, navicular cup, apex obtuse. *Stamens* diadelphous, nine fused together, the vexillary one free, all curved upwards at apex. *Ovary* glabrous, style glabrous, 4–5 mm long, curved upwards at apex, *stigma* punctate. *Pods* 8–25 × 1–2 cm, green, flattened, linear, dehiscent, exocarp raised above the seeds, surface glabrous, black when dry, internally septate. *Seeds* 4–5, 10–12 × 6–8 × 5 mm, oblong, short beaked at one end, each seed separated inside the pod, entirely covered in a thin chartaceous layer of endocarp one side of which extends into a papery samaroid wing forming a compartmented unit that becomes free on maturity, wings 3–5 × 1 cm, hilum eccentric at beaked end of seed, broadly elliptic 2–3 × 2 mm.

##### Endosamara racemosa

Taxon classificationPlantaeFabalesFabaceae

(Roxb.) R.Geesink, Leiden Bot. Ser. 8: 93 (1984)

≡ Robiniaracemosa Roxb., Fl. Ind. ed. 2 vol. 3: 329 (1832)

###### Type.

India, Andra Pradesh, Circar Mts. “Diadelphia decandria. Galuda tiga of the Gentoos”, *Roxburgh s.n..*, E00301096 (E, lecto.!, designated here); *Roxburgh s.n..* E00301095 (E, isolecto.!) ≡ *Tephrosiaracemosa* (Roxb.) Wight & Arn. Prodr. Fl. Pen. Ind. Or. 1: 210 (1834) ≡ *Millettiaracemosa* (Roxb.) Benth. Pl. Jungh. [Miquel] 2: 249 (1853).

#### Key to varieties of *Endosamararacemosa*

**Table:** 

1	Flowers with petals pink or purple	** var. racemosa **
–	Flowers with petals white or pale pink	** var. pallida **

##### Endosamara racemosa var. racemosa

Taxon classificationPlantaeFabalesFabaceae

≡ Wisteriaracemosa (Roxb.) Dalzell, Bombay Flora: 61 (1861)
≡ Phaseoloides [Phaseolodes] *racemosum* (Roxb.) Kuntze, Revis. Gen. Pl. 1: 201 (1891).
= MillettialeiogynaKurz, J. Asiat. Soc. Bengal, Pt. 2, Nat. Hist. 42(2): 67 (1873). Type: Burma, [Myanmar] “Kurz, Martaban [Mottama], in an upper mixed forest at Nakawa Choung, Toukyeghat east of Tounghoo. Fl. April” not found. Type: Upper Burma [Myanmar] Southern Shan State, Toungyi [Taunggyi], comm. Dr [George] King June 1896, 1894, Abdul Khalil s.n.. (K, neo.!, designated here) ≡ Phaseoloides [Phaseolodes] *leiogynum* (Kurz) Kuntze, Revis. Gen. Pl. 1: 201 (1891).

###### Illustration.

Lôc and Vidal in Fl. Cambodge, Laos & Vietnam 30: 16, t. 3 (2001); Plate 1A, B.

###### Distribution.

India; Laos; Myanmar; Malaysia (Peninsula); Philippines; Thailand; Vietnam.

###### Etymology.

The generic name combines endo (endocarp) and samara (the remarkable samaroid winged seeds).

###### Habitat.

In dry woods, thickets and forest margins from sea level to 850 m. climbing over rocks, on banks and among scrub and trees.

##### Endosamara racemosa var. pallida

Taxon classificationPlantaeFabalesFabaceae

(Dalzell) J.Compton & Schrire comb. nov.

urn:lsid:ipni.org:names:77199032-1

≡ Wisteriapallida Dalzell, Bombay Flora: 61 (1861). Type: [Icon] India, “In the Dangs, Wassoorna forest, Bombay, very rare, Dr [Alexander] Gibson” (lecto.!, designated here)
≡ Millettiapallida (Dalzell) Dalzell, J. Linn. Soc., Bot. 13: 187 (1873)

###### Nomenclatural note.

This plate by an unknown artist at the Bombay Botanic Garden at Dapuri, commissioned by Nicol Alexander Dalzell, has the annotation “comm. N. Dalzell 1/72 [January 1872], *Wisteriapallida* Dalz. corrected to *Millettiapallida* Dalz. mss.” in Dalzell’s hand. It is numbered 18 among Dalzell’s artworks in J. D. Hooker’s collections at K and represents the white or pale creamy-yellowish flowered form of this species in western India. It is recombined by us here as var.pallida. Another illustration representing this taxon is of a plant that was cultivated at the Madras Agri-Horticultural Garden. The plant was collected from the Rammanmally [Sandur] Hills, Karnataka by the garden’s superintendent Colonel Francis Archibald Reid and later painted by the artist P. Mooregasan Moodeliar in July 1853. RBGE CAH 27 (Cleghorn Collection, see Noltie 2016: 43)

###### Illustration.

As *Millettiaracemosa*http://florakarnataka.ces.iisc.ac.in/hjcb2/herbsheet.php?id=2056&cat=1

##### 4. Sigmoidala

Taxon classificationPlantaeFabalesFabaceae

J.Compton & Schrire gen. nov.

urn:lsid:ipni.org:names:77198973-1

###### Diagnosis.

The monospecific *Sigmoidalakityana* has several affinities with *Endosamararacemosa* including the absence of bracteoles and glabrous ovaries, characters which also separate it from *Calleryas.str.* which has bracteoles and sericeous ovaries. *Sigmoidala* also shares with *Endosamara* the pubescent floral bracts and broadly campanulate, slightly oblique, subtruncate calyx as noted by Schot (1994: 25) but it was placed in *Callerya* on account of the fruits that lacked the lomented endocarp. The stipules in *Sigmoidala* are shorter, 3–7 mm long (vs. 9–12 mm in *Endosamara*); pedicels shorter, 3–4 mm long (vs. 4–12 mm in *Endosamara* and *Sarcodum*), floral bracts linear, 6–8 mm long (vs. linear-lanceolate, 8–12 mm long in *Endosamara*, 6–20 mm in *Sarcodum*); the back of the standard densely, appressed rufous pubescent (vs. glabrous in *Endosamara* and *Sarcodum*) and the wing petals of *Sigmoidala* are unique within Tribe Wisterieae being a sigmoid shape, reflexed at the midpoint and extending outwards towards the apex (see Fig. 3I). The pods of *Sigmoidala* are flattened, linear to obovate, 7–11 × 1–2 cm (vs. septate, flattened, linear, 10–25 × 1–2 cm in *Endosamara* and botuliform in *Sarcodum*).

###### Type species.

*Sigmoidalakityana* (Craib) J.Compton & Schrire ≡ *Millettiakityana* Craib.

###### Genus description..

robust, twining woody vine. *Stems* very dark green when young, terete. *Leaves* evergreen, coriaceous and nitid when mature, imparipinnate with 7–9 (– 11) leaflets, rachis 12–30 cm long. *Stipules* 3–7 mm long, narrowly deltoid, persistent. *Stipels* 3–6 mm long, linear, persistent. *Leaflets* 7–18 × 2–5 cm, elliptic to narrowly obovate, glabrous above and below, apex cuspidate, margins entire, base cordate. *Inflorescence* a robust many-flowered terminal panicle 20–50 cm long, peduncle sparsely hairy. *Flowers* 16–20 mm long, emerging from August – November. *Floral bracts* 6–8 mm long, caducous, linear. *Bracteoles* absent. *Pedicels* 3–4 mm long, glabrous. *Calyx* 4 × 6 mm, campanulate, green, densely puberulent externally, five lobed, lobes ± equal 1–6 mm long, rounded, obtuse or subtruncate becoming subentire after anthesis. *Standard* 10–12 × 12–13 mm, suborbicular, inner surface white with a pink flush, nectar guide broad, deep golden-yellow, back of standard densely appressed, ferrugineous or rufous pubescent, apex acute or emarginate. Callosities of boss type. *Wing petals* glabrous, longer than keel in length but notably sigmoid towards apex and thereby shortened, each narrowly semi-pandurate 10–14 × 3 mm with basal claws 1–3 mm long. *Keel petals* 10–12 × 4–6 mm, glabrous, united into a long navicular cup, apex acute. *Stamens* diadelphous, nine fused together, the vexillary one free, all curved upwards at apex. *Ovary* glabrous, style glabrous, 3 mm long, curved upwards at apex, *stigma* punctate. *Pods* 7–11 × 1–2 cm, flattened, linear to narrowly obovate, dehiscent, exocarp surface glabrous, speckled with small pustules, subseptate. *Seeds* 1–5(– 8), ellipsoid or orbicular 12–14 × 12–13 × 13 mm, hilum 1.6–2 × 2 mm, elliptic. Fig. 3C–D.

##### Sigmoidala kityana

Taxon classificationPlantaeFabalesFabaceae

(Craib) J.Compton & Schrire comb. nov.

urn:lsid:ipni.org:names:77198984-1

≡ Millettiakityana Craib, Bull. Misc. Inform. Kew 1927(2): 58 (1927). Type: Thailand, “coll. AFG Kerr, locality Chiengmai [Chiang Mai], altitude 300 m. Aug. 23 1914, large woody climber, flowers pink, by village”, Kerr 3347, K000881009 (K, lecto.! designated here); ABDUH: 2/213 (ABD, isolecto.); K000881010 (K, isolecto.!); BM000997330 (BM, isolecto.!); TCD0015789 (TCD, isolecto.!)
≡ Calleryakityana (Craib) Schot, Blumea 39(1–2): 24. (1994)

###### Note.

In the key to the species of *Callerya*, Schot placed this species within the segregating couplet “stipellae persistent” as opposed to “stipellae caducous” and noted that the bracteoles were absent and that the wing petals were longer than the keel (Schot 1994: 9). In our study we have found that the persistent or caducous nature of the stipels is not particularly significant and, moreover, is frequently difficult to verify. Schot also recorded in her species description that the stipules were 3–4 mm long even though Craib had stated that they were 6–8 mm long (Craib 1927: 58). Our observations have confirmed that the stipules rarely exceed 7 mm in length. This very distinct monospecific genus occurs only within a narrow region of northern and north-eastern Thailand (Fig. 3, Plate 1C, D).

###### Illustration.

(as *Millettiakityana*) http://crassa.cocolog-nifty.com/blog/2015/04/millettia-kitya.html

###### Distribution.

Northern Thailand: Chiang Mai, Nan, Lamphun, Sukhothai; North-east Thailand: Loei.

###### Etymology.

The generic name refers to the remarkable sigmoid wing petals.

###### Habitat.

Climbing among dry forest trees in partial sunlight to 400 m. elevation.

### Clade B – *Nanhaia* and *Wisteriopsis*

(Fig. 1; Suppl. material 1: Figs S1–S6)

#### 5. Nanhaia

Taxon classificationPlantaeFabalesFabaceae

J.Compton & Schrire gen. nov.

urn:lsid:ipni.org:names:77198974-1

≡ MillettiaSect.Corynecarpae Z.Wei, Acta Phytotax. Sin. 23(4): 281 (1985)

##### Diagnosis.

*Nanhaia*, with two species, is readily distinguished from *Wisteriopsis* by the densely pubescent or sericeous ovaries (glabrous in *Wisteriopsis*) and the larger flowers frequently 15–35 mm long (vs *Wisteriopsis* 7–15 mm long). In *Nanhaia* the stipules arise immediately above the swollen, hardened gibbosities (Fig. 4).

##### Type species.

*Nanhaiaspeciosa* (Champ. ex Benth.) J.Compton & Schrire ≡ *Millettiaspeciosa* Champ. ex Benth.

##### Genus description.

Procumbent or scandent twining vines, 1–5 m high, scrambling among rocks and scrub. *Stems* green or brown, terete, pubescent. *Leaves* with 5–17 leaflets, evergreen, glabrous or with a few scattered hairs below, imparipinnate, rachis 3–30 cm long. *Stipules* 2–4 mm long, linear or deltoid, caducous in *N.fordii* (persistent *N.speciosa*). *Stipels* 1–3 mm long, linear, persistent. *Leaflets* 3–9 × 1–4 cm, ovate-elliptic or narrowly elliptic, glabrescent or sparsely hairy, apex acuminate or cuspidate, margins entire, base rounded to subcordate. *Inflorescence* erect or pendant sometimes leafy panicles 4–20 cm long, frequently comprising several leafy lateral racemes, peduncle yellow tomentose or densely brown pubescent. *Flowers* 16–32 mm long, emerging from June to September. *Floral bracts* 3–7 mm long, linear or narrowly deltoid, persistent (caducous in *N.fordii*). *Bracteoles* at base of calyx 1–5 mm long, narrowly ovate or elliptic, persistent. *Pedicels* 4–11 mm long, glabrous or pubescent. *Calyx* 4–6 × 5–9 mm campanulate, oblique, pubescent externally, (densely pubescent internally on *N.fordii*) five lobed, teeth unequal, 1–3 mm long, acute. *Standard* 12–18 × 11–18 mm, suborbicular, white, cream or pink, nectar guide pale or dark green, back of standard glabrous, apex acute or obtuse. Callosities of boss type. *Wing petals* 12–17 × 4–6 mm, glabrous, subequal to the keel, each narrowly semi-pandurate, slightly curved upwards at the apex; free from the keel, apex obtuse, basal claws 2–5 mm long. *Keel petals* 12–16 × 4–6 mm, glabrous, united into a falcate, navicular cup, apex obtuse, basal claw 4–9 mm long. *Stamens* diadelphous, nine fused together, the vexillary one free, all curved upwards at apex. *Ovary* densely sericeous, especially along thickened margins, style ciliate (*N.speciosa*) or glabrous (*N.fordii*), 2–3 mm long curved upwards at apex, *stigma* punctate. *Pods* 10–20 × 1–2 cm, flat, linear, dehiscent, surface pubescent to densely brown tomentose, brown and hard when dry, subseptate. *Seeds* 2–10, ovoid or ellipsoid, 10–12 × 5–12 × 1–7 mm, hilum terminal or central, elliptic, 2–3 × 1 mm. Fig. 4.

##### Distribution.

China (Fujian, Guangdong, Guangxi, Guizhou, Hainan, Hunan, Yunnan); Vietnam (north).

##### Etymology.

Nanhai is the Chinese name for the South China Sea which links southern China with Vietnam.

#### Key to species of *Nanhaia*

**Table:** 

2	Flowers 15–19 mm long; bracts 3–4 mm wide, narrowly deltoid, caducous; leaves 5–7 foliolate	*** N. fordii ***
–	Flowers 20–32 mm long; bracts 4–7 mm wide, deltoid-lanceolate, persistent; leaves 7–17 foliolate	*** N. speciosa ***

##### Nanhaia speciosa

Taxon classificationPlantaeFabalesFabaceae

(Champ. ex Benth.) J.Compton & Schrire comb. nov.

urn:lsid:ipni.org:names:77198985-1

≡ Millettiaspeciosa Champ. ex Benth., Hooker’s J. Bot. Kew Gard. Misc. 4: 73 (1852). Type: China, “Hong Kong, Millett 505”, K000881029 (K, lecto.!, designated by Schot (1994) but see note below)
≡ Phaseoloides [Phaseolodes] speciosa (Champ. ex. Benth.) Kuntze, Revis. Gen. Pl. 1: 201 (1891)
≡ Calleryaspeciosa (Champ. ex Benth.) Schot, Blumea 39(1–2): 32 (1994)

###### Nomenclatural note.

Schot selected a specimen at K, *Champion* 505 as lectotype for the name *Millettiaspeciosa* Champ. ex Benth. (Schot 1994: 32). This was, however, clearly an error because another specimen, *Millett* 505, was cited in the protologue and determined as type by Dunn’s annotation on the sheet at K prior to the publication of his monograph on *Millettia* in 1912. Dunn had incorrectly cited the specimen as *Champion* 505 and not *Millett* 505 which may explain Schot’s error (Dunn 1912a: 155). Bentham’s paper on the plants of Hong Kong was based on the collections of Major John George Champion (1815–1854). There are two specimens at K collected by Champion in the Hooker Herbarium: K000881027 and K000881028. Both are annotated “Champion 261, Hong Kong”, but these specimens were not specifically cited by Bentham in the protologue which merely stated “on Victoria Peak” [on Hong Kong island] and are therefore regarded as uncited specimens that comprise part of the original material associated with the name (Art. 9.4(a)). The Millett collection K000881029 is annotated “505 *Millettiagrandiflora* sp. n. fls white and yellow Hong Kong” and is dated 1854 in Bentham’s Herbarium. This collection was cited in the protologue “it is in the Hookerian Herbarium from Millett’s collection” and is therefore a syntype in the absence of any cited holotype (Art. 9.6). Under Art. 9.12 a syntype takes precendence over any uncited specimens when selecting a lectotype (Turland et al. 2018). *Millett* 505 is therefore the obligate lectotype (N. Turland pers. comm.).

###### Illustrations.

Lôc and Vidal in Fl. Cambodge, Laos & Vietnam 30: 37 (2001); Wei and Pedley Fl. China 10: 182, t. 215 [1–7] (2010). http://www.fpcn.net/a/tengmanzhiwu/20131008/Callerya_speciosa.html (as *Calleryaspeciosa*). Fig. 4.

###### Distribution.

China (Fujian, Guangdong, Guangxi, Guizhou, Hainan, Hunan, Yunnan); Vietnam (north).

###### Habitat.

In open forest, edges of ravines and thickets from 100–300 m.

##### Nanhaia fordii

Taxon classificationPlantaeFabalesFabaceae

(Dunn) J.Compton & Schrire comb. nov.

urn:lsid:ipni.org:names:77198986-1

≡ Millettiafordii Dunn, J. Linn. Soc., Bot. 41: 156 (1912a). Type: China, Guandong, “Comm. Ford, coll. August 1887, Kwangtung Prov. Lienchow River”, [Charles] Ford 62, K000881044 (K, holo.!); P02141771 (P, iso.!); A00065311 (A, iso!)
≡ Calleryafordii (Dunn) Schot, Blumea 39(1–2): 23 (1994)

###### Illustrations.

Lôc and Vidal in Fl. Cambodge, Laos & Vietnam 30: 36 (2001); Wei and Pedley Fl. China 10: 183, t. 215 [8–12] (2010). http://www.plantphoto.cn (in Home Page enter *Calleryafordii*)

###### Distribution.

China (Guandong, Guangxi); Vietnam (north).

###### Habitat.

Trailing among rocks and thickets in open sites from sea level to 200 m.

##### 6. Wisteriopsis

Taxon classificationPlantaeFabalesFabaceae

J.Compton & Schrire gen. nov.

urn:lsid:ipni.org:names:77198975-1

≡ MillettiaSect.Eurybotryae Dunn, J. Linn. Soc., Bot. 41: 135 (1912a)

###### Diagnosis.

The five species of *Wisteriopsis* (Fig. 5) possess a fine ring of hairs lining the inner rim of the calyx cup, prominent gibbosities below the stipules and persistent bracts at the base of pedicels (vs. bracts caducous in *Calleryas.str.*). *Wisteriopsis* has glabrous standards (vs. sericeous in *Calleryas.str.*) and wings equalling or slightly shorter than the keel in length (vs. wings much shorter than the keel in *Callerya*). The unique character of this genus is the staminal column being visible between wings and keel at anthesis, becoming free from the keel after pollinator tripping. See under *Nanhaia* for comparisons with *Wisteriopsis*.

###### Type species.

*Wisteriopsisjaponica* (Siebold & Zucc.) J.Compton & Schrire ≡ *Wisteriajaponica* Siebold & Zucc.

###### Genus description.

Robust twining woody vines attaining 4–18 m in height climbing over shrubs or sprawling over rocks. *Stems* greyish brown or brown, terete, young branches finely grey or brown tomentose becoming glabrous (or densely ferrugineous tomentose in *W.eurybotrya*). *Leaves* deciduous (in *W.japonica*) or evergreen, chartaceous or coriaceous, imparipinnate, rachis 9–20 cm long. *Stipules* 2–4 mm long, persistent or caducous, emerging from a subulate or mounded gibbosity. *Stipels* 1–5 mm long, persistent or caducous, petiolules 3–4 mm. *Leaflets* (5 –) 7–15, in opposite pairs, ovate-lanceolate, elliptic, ovate or ovate-oblong (linear-lanceolate in W.reticulatavar.stenophylla) 15–40 × 5–20 mm, upper surface glabrous with reticulate venation (smooth and shiny in *W.championii)*, lower surface paler and glabrous or with hairs along veins, apex obtuse, acute, acuminate or cuspidate, margins entire, base cordate or cuneate. *Inflorescence* 8–40 cm long, laterally paniculate and sometimes racemose in leaf axils, acropetal, erect or pendulous, sparsely hairy or glabrous. *Flowers* 7–16 mm long emerging from April to August. *Floral bracts* persistent, (caducous in *W.eurybotrya*), linear to subulate, 1–6 mm long, subtending base of pedicel. *Bracteoles* subtending and adnate to base of calyx, linear, acuminate 1–2 mm long, persistent. *Pedicels* elongating at flower maturity, 2–8 mm long, glabrous or pubescent. *Calyx* tubular, cupuliform or campanulate, 1.5–4 × 2–6 mm, glabrous or pubescent externally, with an annulus of fine hairs at the mouth presenting a ciliate margin, five lobed, lobes more or less equal, upper lobes obtuse or deltoid, lower lobes bluntly acute. *Standard* 6–12 × 4–10 mm, white sometimes flushed pale pink, greenish white, pink or purple, ovate or suborbicular, slightly deflexed backwards near the base, back of standard glabrous, inside with yellow or green nectar guide, callosities of boss type. *Wing petals* 5–13 × 2–4 mm, equal in length to the keel petals, each semi-pandurate with a short pair of auricles near the base and a prominent basal claw 1–3 mm long. Wing petals free of the keel at time of anthesis. *Keel petals* glabrous, united into a semi-pandurate cup, base with conjoined parallel claws, apex obtuse (acute in *W.eurybotrya*). *Stamens* diadelphous, nine fused together, the vexillary one free, all curved upwards at apex, staminal column visible between wings and keel at anthesis, becoming free from keel after pollinator tripping (tardily so in *W.reticulata*). *Ovary* glabrous, style 2–3 mm long, curved upwards at apex, *stigma* punctate. *Pods* 65–125 × 8–30 mm, linear, ovate or narrowly elliptic, compressed, (inflated in *W.eurybotrya*), pale green becoming very dark brown, cartilaginous and tardily dehiscent, exocarp glabrous externally (margins thickened in *W.eurybotrya*), surface finely corrugated, endocarp chartaceous, pale cream, detaching tardily from the exocarp, the seeds in hollow cavities, pods dehiscing explosively, the valves opening straight or twisting to release the seeds, subseptate. *Seeds* (1 –)6–8, lenticular, suborbicular to oblate-spheroidal, smooth, brown, 5–28 × 4–20 × 1–5 mm, hilum 1–2 × 1 mm, elliptic. Fig. 5.

###### Distribution.

China (Anhui, Fujian, Guizhou, Guangdong, Guangxi, Hainan, Hubei, Hunan, Jiangsu, Jiangxi, Zhejiang); Japan (Honshu, Kyushu, Shikoku); Laos; South Korea (North Gyeongsang, South Gyeongsang, North Jeolla, South Jeolla); Thailand; Vietnam.

###### Etymology.

Wisteria – opsis = like (Gk), resembling the genus *Wisteria*.

#### Key to species of *Wisteriopsis*

**Table:** 

1	Inflorescences of true terminal panicles without leaves subtending flowering branches	**2**
–	Inflorescences largely of racemes aggregated terminally on branches, such that the entire flowering region appears as a leafy panicle with only the occasional leaf absent at the base of a flowering branch	**4**
2	Inflorescence 20–30 cm long; apex of wing and keel petals obtuse; pods narrow 5–15 mm wide, not constricted between seeds; seeds 1–6, lenticular, 7–10 × 6–9 mm	**3**
–	Inflorescence 30–40 cm long; apex of wing and keel petals acute; pods broad 25–35 mm wide, noticeably constricted between seeds and with thickened dorsal and ventral sutures; seeds 3–6, ellipsoid or suborbicular 15–35 × 20–25 mm	*** W. eurybotrya ***
3	Leaflets oblong-ovate, apex acuminate; flowers white, nectar guides green	*** W. championii ***
–	Leaflets lanceolate or linear, apex acute; flowers red, pink or purple, nectar guides yellow	*** W. reticulata ***
4	Leaves deciduous, leaflets 9–15 narrowly ovate-lanceolate; flowers 6–12 mm long	*** W. japonica ***
–	Leaves evergreen, leaflets 5–7 broadly ovate; flowers 12–15 mm long	*** W. kiangsiensis ***

##### Wisteriopsis japonica

Taxon classificationPlantaeFabalesFabaceae

(Siebold & Zucc.) J.Compton & Schrire comb. nov.

urn:lsid:ipni.org:names:77198989-1

###### Type.

Japan, “*Wisteriajaponica* Fl. Jap. t. 43. Hb. de Siebold 1829”, *Siebold* s.n.. L0059625 (L, lecto.!, designated by Compton and Thijsse (2013)); Japan, “Herb. Lugd. Batav. Coll. *Dr von Siebold s.n..* donnée par Mr. Blume, *Wisteriajaponica* Zucc.” P02141817 (P, isolecto.!)

#### Key to the varieties of *Wisteriopsisjaponica*

**Table:** 

1	Flowers with all petals cream coloured, pale yellow or greenish-white	** var. japonica **
–	Flowers with standard white, wings and keel pink	** var. alborosea **

##### Wisteriopsis japonica var. japonica

Taxon classificationPlantaeFabalesFabaceae

≡ Wisteria japonica Siebold & Zucc., Fl. Jap.: 88. (1839)

≡ Millettiajaponica (Siebold & Zucc.) A.Gray, Mem. Amer. Acad. Arts, n.s., 6: 386. (1858)
≡ Phaseoloides [Phaseolodes] *japonicum* (Siebold & Zucc.) Kuntze, Revis. Gen. Pl. 1: 201. (1891)
≡ Kraunhiajaponica (Siebold & Zucc.) Taub., Engler & Prantl, Nat. Pflanzen- fam. 3(3): 271. (1894).

###### Illustration.

Compton in Curtis’s Bot. Mag. 32 (3–4): 379, t. 2 (2015); Fig. 5, Plate 3A–D.

###### Distribution.

Japan in south-western Honshu, Shikoku and Kyushu islands; South Korea in North and South Gyeongsang and North and South Jeolla provinces.

###### Habitat.

In woods and forests from sea level to 1200 m, climbing among scrub and trees.

##### Wisteriopsis japonica var. alborosea

Taxon classificationPlantaeFabalesFabaceae

(Sakata) J.Compton & Schrire comb. nov.

urn:lsid:ipni.org:names:77198990-1

≡ Millettiajaponica(Siebold & Zucc.)A.Grayf.alborosea Sakata, J. Jap. Bot. 33(1): 30 (1958). Type: Japan, Bansei, Kaseda-shi 18 Aug. 1957, T.Sakata s.n.. (KAG, holo.)
≡ WisteriajaponicaSiebold & Zucc.f.alborosea (Sakata) Yonek., J. Jap. Bot. 80(6): 325 (2005)

###### Note.

The holotype has not been located at KAG where Toshio Sakata’s material had been incorporated (Koji Yonekura pers. comm.). No material was available on which to neotypify this taxon.

###### Distribution.

Japan, Kyushu Island.

##### Wisteriopsis kiangsiensis

Taxon classificationPlantaeFabalesFabaceae

(Z.Wei) J.Compton & Schrire comb. nov.

urn:lsid:ipni.org:names:77198991-1

≡ Millettiakiangsiensi***s*** Z.Wei, Acta Phytotax. Sin. 23(4): 283 (1985). Type: China, Jiangxi Prov., Wuning, *Y.G.Xiong* 4143 (LBG, holo.); Paratype: China, Anhui, *Qin Renchang* [*Ren-Chang Ching*] 2881, elev. 250 m. 30 June 1925, “climber 7 m high”, PE00417692 (PE!, K!)
≡ Calleryakiangsiensis (Z.Wei) Z.Wei & Pedley, Flora of China 10: 184, t. 219 [8–15] (2010)
= Millettiakiangsiensisf.purpurea Z.H.Cheng, J. Zhejiang Forest. Coll. 4: 70 (1987). (ZJFC, holo.). China, Zhejiang, Tonglu. This may represent a purple flowered variant of the species but we have not seen any material in order to verify this.

###### Note.

This species described from Jiangxi [originally as Kiangsi] Province in south-eastern China, is closely allied to *W.japonica*. Characters that distinguish *W.kiangsiensis* from *W.japonica* are the evergreen leaves in *W.kiangsiensis* with 7–9 broadly ovate leaflets (vs. deciduous leaves with (7 –) 9–15, narrowly ovate-lanceolate leaflets in *W.japonica*). The flowers are white, frequently flushed with pale rose pink and 12–15 mm long in *W.kiangsiensis*, (vs. flowers yellowish-white, 6–12 mm long in *W.japonica*). Both species possess a conspicuous annulus of fine hairs at the mouth of the calyx and narrow lanceolate stipules that arise directly from a deltoid gibbosity positioned on the branch immediately subtending the leaf rachis.

###### Illustrations.

Wei and Pedley, Flora of China 10: 184, t. 219 [8–15] (2010). http://www.plantphoto.cn (In Home Page enter *Calleryakiangsiensis*).

###### Distribution.

China (Anhui, Fujian, Hubei, Hunan, Jiangsu, Jiangxi, Zhejiang).

###### Habitat.

open sites clambering over scrub among woods and forests up to 500 m.

##### Wisteriopsis reticulata

Taxon classificationPlantaeFabalesFabaceae

(Benth.) J.Compton & Schrire comb. nov.

urn:lsid:ipni.org:names:77198992-1

###### Type.

China, “woodland hedges, flowers purple, vexillum exauriculatum ecallosum. stam. vexillum liberum, ovarium glabrum, north China comm. Fortune 1845”, *R.Fortune* A95 s.d., K000881030 (K, lecto.! designated here); K000881031 (K, isolecto.!); “Glycine sp. purple, woods and hedges, north of China, August 1844”, *R.Fortune* A95, P02141772 (P, isolecto.!); P02141773 (P, isolecto.!); P02141774 (P, isolecto.!); M0233437 (M, isolecto.!)

#### Key to varieties of *Wisteriopsisreticulata*

**Table:** 

1	Leaflets ovate-elliptic or oblong, 15–40 mm wide	** var. reticulata **
–	Leaflets linear or narrowly lanceolate, 5–12 mm wide	** var. stenophylla **

##### Wisteriopsis reticulata var. reticulata

Taxon classificationPlantaeFabalesFabaceae

≡ Millettiareticulata Benth. Pl. Jungh. [Miquel] 2: 249 (1852)
≡ Phaseoloides [Phaseolodes] reticulata (Benth.) Kuntze, Revis. Gen. Pl. 1: 201. (1891)
≡ Calleryareticulata (Benth.) Schot, Blumea 39(1–2): 30 (1994).
= Millettiacognata Hance, J. Bot. 18: 260 (1880). Type: China, Hunan “in collibus demissis ad fl. Siang [Xiang] reg. septent. prov. Hunan aest. 1878 Herb. Hance 20708”, *T.L.Bullock*s.n.., BM001217087 (BM, holo.!); A00065365 (A, iso.!)
≡ Phaseoloides [Phaseolodes] cognata ( Hance) Kuntze, Revis. Gen. Pl. 1: 201. (1891).
= Millettiapurpurea Yatabe, Bot. Mag. (Tokyo) 6: 379 (1892). Type: Japan [Icon] Bot. Mag. (Tokyo) 6: t. 12 (1892), (lecto.!, designated here).

###### Illustrations.

Lôc and Vidal in Fl. Cambodge, Laos & Vietnam 30: 32 (2001); Wei and Pedley Fl. China 10: 183, t. 216 [7–14] (2010). https://singapore.biodiversity.online (*Calleryareticulata*) https://www.flickr.com/photos/reulim/34692959202; https://florafaunaweb.nparks.gov.sg/special-pages/plant-detail.aspx?id=3329 (Plate 3E).

###### Distribution.

China (Anhui, Fujian, Guangdong, Guangxi, Guizhou, Hainan, Hubei, Hunan, Jiangsu, Jiangxi, southern Shaanxi, Sichuan, Taiwan, Yunnan and Zhejiang); Vietnam north.

###### Habitat.

On open slopes covering wooded thickets from sea level to 1000 m.

##### Wisteriopsis reticulata var. stenophylla

Taxon classificationPlantaeFabalesFabaceae

(Merr. & Chun) J.Compton & Schrire comb. nov.

urn:lsid:ipni.org:names:77198993-1

≡ Millettiareticulatavar.stenophylla Merr. & Chun, Sunyatsenia 5: 83 (1940). Type: China, Hainan, Yaichow [Yazhou], “29 May 1933, Longzhou, Licai, alt. 800 ft. vine on rocks twining on shrubs, fls purplish-red fragrant”, *F.C.How* 70826, IBSC000909 (IBSC, holo.); US02324730 (US, iso.!); P02754288 (P, iso.!); (SYS, iso.); NAS00391016 (NAS, iso.!); IBK00073921 (IBK, iso.!). Paratype: China, Hainan, Po-ting, [Baoting]. *F.C.How* 73744 “elev. 1200 ft. along streams, twining, 26 September 1935” A00063957 (A!)
≡ Calleryareticulatavar.stenophylla (Merr. & Chun) X.Y.Zhu, Legumes of China (ILDIS) 455 (2007)

###### Illustration.

Wei and Pedley Fl. China 10: 184, t. 216 [15] (2010).

###### Distribution.

China, Hainan Island.

###### Habitat.

Open sites in tropical forest.

##### Wisteriopsis championii

Taxon classificationPlantaeFabalesFabaceae

(Benth.) J.Compton & Schrire comb. nov.

urn:lsid:ipni.org:names:77198994-1

≡ Millettiachampionii Benth., Hooker’s Journ. Bot. Kew Gard. Misc. 4: 74 (1852). Type: China, Guangdong, Hong Kong, [John George] Champion 263, lower specimen K000881035 (K, lecto.! designated here); China, Guangdong, Hong Kong, Champion 263, upper specimen K000881036 (K, isolecto.!)
≡ Phaseoloides [Phaseolodes] championii (Benth.) Kuntze, Revis. Gen. Pl. 1: 201. (1891)
≡ Millettiareticulatavar.championii (Benth.) H.Sun, Fl. Yunnanica 10: 404 (2006)
≡ Calleryachampionii (Benth.) X.Y.Zhu, Legumes of China (ILDIS) 450 (2007)

###### Illustration.

Wei and Pedley Fl. China 10: 184, t. 219 [1–7] (2010). http://www.hkwildlife.net/Forum/viewthread.php?tid=4986&page=1 (as *Millettiachampionii*).

###### Distribution.

China (Fujian, Guandong, Guangxi, Hong Kong and Jiangxi).

###### Habitat.

In thickets beside rocky valleys climbing among rocks and scrub from 200 to 800 m.

##### Wisteriopsis eurybotrya

Taxon classificationPlantaeFabalesFabaceae

(Drake) J.Compton & Schrire comb. nov.

urn:lsid:ipni.org:names:77198995-1

≡ Millettiaeurybotrya Drake, J. Bot. (Morot) 1891: 187 (1891). Type: Vietnam, “Tonkin, Thu Phap [Van Hoa, Hanoi], liane, corolle rose, 1887”, [Gaspard Joseph Benedict] *Balansa* 2304, P02141769 (P, lecto.!, designated by Schot (1994): P02141770 (P, isolecto.!); K000881012 (K, isolecto.!). Paratypes: *B.Balansa* 2303, “Tu-Phap dans les bois, August 1887” P02753447 (P!); *B.Balansa* 2303 P00852397 (P, isopara!); *B.Balansa* 2300, “Tu-Phap dans les bois, September 1887” P02753453 (P!); *B.Balansa* 2300 P027533454 (P!); P02753446; *B.Balansa* 2301, “Tonkin, Tho-bo dans les forets, November 1887” (P!)
≡ Calleryaeurybotrya (Drake) Schot, Blumea 39(1–2): 22 (1994)

###### Illustrations.

Lôc and Vidal in Fl. Cambodge, Laos & Vietnam 30: 30, t. 7 (2001); Wei and Pedley Fl. China 10: 182, t. 216 [1–6] (2010). http://www.plantphoto.cn (as *Calleryaeurybotrya*).

###### Distribution.

China (Guangdong, Guizhou); Thailand; Vietnam.

###### Habitat.

In thickets, river margins and on the edge of evergreen forests from sea level to 400 m.

### Clade C – *Callerya, Serawaia, Whitfordiodendron, Kanburia* and *Afgekia*

Fig. 1; Supp. material 1: Figs S1–S6

#### 7. Callerya

Taxon classificationPlantaeFabalesFabaceae

Endl., Gen. Pl. Suppl. 3: 104 (1843), emend nov. J.Compton and Schrire

≡ MillettiaSect.Curvistylae Z.Wei, Acta Phytotax. Sin. 23(4): 284 (1985)
= MillettiaSer.Dielsianae Z.Wei, Acta Phytotax. Sin. 23(4): 284 (1985) Type species: Millettiadielsiana Harms ex Diels, Bot. Jahrb. Syst. 29(3–4): 412 (1900).
= MillettiaSer.Oospermae Z.Wei, Acta Phytotax. Sin. 23(4): 284 (1985) Type species: Millettiaoosperma Dunn, J. Linn. Soc. Bot. 41: 157 (1912a).

##### Diagnosis.

*Calleryas.str.* is here recognised as comprising *C.nitida*, *C.bonatiana*, *C.cochinchinensis*, *C.cinerea* and *C.dielsiana*. The Flora of China account (Wei and Pedley 2010), included a further eight species: *C.tsui*, *C.dorwardii*, *C.sphaerosperma*, *C.congestiflora*, *C.longipedunculata*, *C.gentiliana*, *C.oosperma* and *C.sericosema* which we have not been able to ascertain the status of due to a paucity of study material. Their descriptions in Wei and Pedley (2010), however, indicate that they should be placed in *Calleryas.str.* The flowers in *Calleryas.str.* have wing petals shorter than the keel petals (vs. equal or longer in *Kanburia* and *Whitfordiodendron*). The standard is also proportionately larger than in *Kanburia* and *Whitfordiodendron*. The keel is glabrous in *Calleryas.str.* (vs. sericeous in *Whitfordiodendron*).

##### Type species.

*Calleryanitida* (Benth.) R.Geesink ≡ *Millettianitida* Benth.

##### Genus description.

Short scandent vines scrambling over rocks or shrubs to 0.5–1 m tall, or tall scrambling climbers to 20 m tall. *Stems* grey, yellowish or brown, terete, pubescent or glabrescent. *Leaves* with 3–13 leaflets, evergreen, glabrous or strigose, (villose in *C.bonatiana*) imparipinnate, rachis 3–16 (– 40) cm long. *Stipules* 1–4 mm long, deltoid, caducous (persistent on *C.nitida*). *Stipels* 2–7 mm long, linear, persistent (absent in *C.bonatiana*). *Leaflets* 3–15 (– 22) x 2–6 (– 10) cm, terminal leaflet distinctly larger than laterals, basal pair usually smallest; lateral leaflets, ovate or obovate or lanceolate, ovate-elliptic or narrowly elliptic, glabrous or pubescent (densely villose below in *C.bonatiana*), apex obtuse or acute or acuminate, margins entire, base rounded, cuneate or subcordate. *Inflorescence* a terminal panicle 6–20 (– 40) cm long, (racemes axillary 8–12 cm long in a leafy panicle in *C.bonatiana*), peduncle yellow or brown puberulous or tomentose. *Flowers* 11–25 mm long, emerging from March – November. *Floral bracts* 1–6 mm long, narrowly ovate, deltoid or linear, caducous. *Bracteoles* at top of pedicel 1–6 mm long, (reflexed in *C.nitida*) narrowly ovate, deltoid or linear, caducous or persistent. *Pedicels* 2–10 mm long, tomentose or puberulent. *Calyx* 3–12 × 4–10 mm broadly campanulate, oblique, sparsely pubescent or densely sericeous externally, five lobed, teeth unequal 1–6 mm long, obtuse, subtruncate or acute. *Standard* 12–25 × 8–17 mm, elliptic or ovate, white, pink, lilac, red, mauve, violet, green or purple, nectar guide yellow or green, back of standard densely white, yellow or brown sericeous, apex acute, retuse or obtuse (nectar guide fringed with hairs on inner face on *C.bonatiana*), callosities of ridge or boss type. *Wing petals* 5–15 × 2–5 mm, glabrous, shorter than the keel, each narrowly obovate, straight at apex; free from the keel, apex obtuse, basal claws 2–5 mm long. *Keel petals* 8–16 × 3–6 mm, glabrous, united into a falcate, navicular cup, apex obtuse. *Stamens* diadelphous, nine fused together, the vexillary one free, all curved upwards at apex. *Ovary* densely sericeous, tomentose or velutinous, style 6–9 mm long, ciliate at base (*C.cochinchinensis*) or glabrous, curved upwards at apex, *stigma* punctate. *Pods* 4–15 × 1.5–4 cm, flat or inflated, linear, linear-oblong, rhomboid-oblong rarely globose, straight or torulose, dehiscent, surface grey, brown or yellow tomentose, subseptate. *Seeds* (1 –) 2–5, ovoid, orbicular, oblately-spheroid or ellipsoid, 8–30 × 6–35 × 2–20 mm, hilum central, elliptic or oval 2–5 × 0.5–1 mm.

##### Distribution.

Bangladesh; Bhutan; China (Anhui, Fujian, Guandong, Guangxi, Guizhou, Hainan, Hubei, Hunan, Jiangxi, Sichuan, Shaanxi, Yunnan, Zhejiang); India; Laos; Myanmar; Nepal; Thailand; Vietnam.

##### Etymology.

The genus *Callerya* is named after Joseph Gaetan Pierre-Maxime-Marie Callery (1810–1862) scholar, missionary and sinologist.

#### Key to species of *Callerya* recognized in this treatment

**Table:** 

1	Leaves 11–13 foliolate; standard bright green, nectary guide surrounded by sericeous hairs on inner face; wing petals lilac-purple; style glabrous	*** C. bonatiana ***
–	Leaves 3–5 foliolate; standard glabrous within	**2**
2	Leaves 3(– 5) foliolate; standard white sometimes flushed green, style ciliate or sericeous at base; seeds 1–2	*** C. cochinchinensis ***
–	Leaves 5-foliolate; standard pink, lilac, red or purple, style glabrous; seeds (1 –)3–5	**3**
3	Stipels subulate, 1–2 mm long; bracteoles reflexed; pods linear-oblong, not inflated	*** C. nitida ***
–	Stipels linear 3–4 mm long; bracteoles straight; pods inflated	**4**
4	Floral bracts lanceolate 3–5 mm long; seeds 3–5 per pod, 8 × 6 mm, tawny brown, oblong or suborbicular	*** C. dielsiana ***
–	Floral bracts narrowly ovate to linear 2–6 mm long; seeds (1 –)2–4 per pod, 18 × 14 mm, dark violet, ellipsoid	*** C. cinerea ***

##### Callerya nitida

Taxon classificationPlantaeFabalesFabaceae

(Benth.) R.Geesink, Leiden Bot. Ser. 8: 83 (1984)

###### Type.

“Hong Kong, China, received by W. J. Hooker 1841”, Mr Millett [Charles] s.n.., K000881042 (K, lecto.! designated here); K000881039 (K, isolecto.!); K000881043 (K, isolecto.!)

#### Key to varieties of *Calleryanitida*

**Table:** 

1	Flowers 22–24 mm long; leaflets 50–90 (– 110) × 30–40 mm., surfaces glabrous or sparsely pubescent below, apex acute	** var. nitida **
–	Flowers 16–18 mm long; leaflets 35–55 × 20–30 mm	**2**
2	Leaflets narrowly lanceolate, both surfaces glabrous, apex acuminate	** var. minor **
–	Leaflets ovate, densely reddish-brown hirsute below, rough but glabrous above, apex cuspidate	** var. hirsutissima **

##### Callerya nitida var. nitida

Taxon classificationPlantaeFabalesFabaceae

≡ Millettianitida Benth., London J. Bot. 1: 484 (1842)
≡ Phaseoloides [Phaseolodes] nitida (Benth.) Kuntze, Revis. Gen Pl. 1: 201 (1891)
= Millettiakueichouensis Hu, Acta Phytotax. Sin. 3: 356 (1954). Type: China, Guizhou, Fengxiangxi, 10 June 1928, *P.Q.* [Pu Chiu] Tsoong 749, PE00022411 (PE, holo.!).

###### Illustrations.

Schot, Blumea 39(1–2): 27, t. 3 (1994); Wei and Pedley Fl. China 10: 182, t. 221 [1–8] (2010). http://lalajacky.blogspot.com/2008/11/blog-post_5886.html (as *Millettianitida*).

###### Distribution.

China (Fujian, Guangdong, Guangxi, Guizhou, Hainan, Hunan, Jiangxi, Sichuan, Taiwan, Yunnan, Zhejiang).

###### Habitat.

In thickets and lowland forest margins from sea level to 1500 m.

##### Callerya nitida var. minor

Taxon classificationPlantaeFabalesFabaceae

(Z.Wei) X.Y.Zhu, Legumes China 454 (2007)

≡ Millettianitidavar.minor Z.Wei, Acta Phytotax. Sin. 23: 288 (1985). Type: China, Sichuan, Mt. Emei elev. 900 m., Lushan county, Bailongdong, Houshan, 4 September 1957, *G.H.*[Guang Hui] Yang 57095, PE01432522 (PE, holo.!)

###### Illustration.

Wei and Pedley Fl. China 10: 182, t. 221 [9] (2010).

###### Distribution.

China (Fujian, Guangdong, Guangxi, Hunan, Jiangxi).

###### Habitat.

in thickets and along forest margins and open places 500 to 1000 m.

##### Callerya nitida var. hirsutissima

Taxon classificationPlantaeFabalesFabaceae

(Z.Wei) X.Y.Zhu, Legumes China 454 (2007)

≡ Millettianitidavar.hirsutissima Z.Wei, Acta Phytotax. Sin. 23: 288 (1985). Type: China, Hunan, Zixing, *B.H.* [Pao Han] *Liang* 85983 (IBSC, holo.)

###### Illustration.

Wei and Pedley Fl. China 10: 182, t. 221 [10] (2010).

###### Distribution.

China (Fujian, Guangdong, Guangxi, Guizhou, Hunan, Jiangxi, Sichuan, Yunnan, Zhejiang).

###### Habitat.

in thickets and along forest margins 800 to 1500 m.

##### Callerya dielsiana

Taxon classificationPlantaeFabalesFabaceae

(Harms ex Diels) P.K.Lôc ex Z.Wei & Pedley, Fl. China 10: 187, t. 222 [1–7] 2010

≡ Millettiadielsiana Harms ex Diels, Bot. Jahrb. Syst. 29(3–4): 412 (1901). Type: China “Setchuen [Sichuan] ab incolis collectae” fl. Nanchuan, s.d. 1899, [Carl] Bock & [Augustus] *von Rosthorn* 1626, V-2014532 (O, lecto.! designated here); A00339036 (A, isolecto.!) Paratype: China, Sichuan, Nanchuan s.d., Bock & Rosthorn 1638, V-2014531 (O!)

###### Distribution.

China (Anhui, Fujian, Gansu, Guangdong, Guangxi, Guizhou, Hainan, Hubei, Hunan, Jiangxi, Shaanxi, Sichuan, Yunnan, Zhejiang).

###### Habitat.

In open places, mixed woods and forest margins from 300 to 2500 m.

##### Callerya bonatiana

Taxon classificationPlantaeFabalesFabaceae

(Pamp.) P.K.Lôc, Bot. Zhurn. (Moscow & Leningrad) 81(10): 99 (1996)

≡ Millettiabonatiana Pamp., Nuovo Giorn. Bot. Ital. 17: 24 (1910). Type: China, Yunnan “pagoda de Ke-long, 23 Mai 1904, plante grimpante” [François] *Ducloux* 380, FI-1018361 (FI, lecto.! designated here); FI-1018360 (FI, isolecto.!); Paratypes: China, Yunnan, “Juin 1904, fleurs vertes, grimpes au sommet des arbres”, [Edouard-Ernest] *Maire* 156, FI-1018363 (FI!); P03583784 (P, isopara.!); China, Yunnan, [E.E] *Maire* 196, “Octobre 1904, fruits de la grande legumineuse a fleurs vertes” FI-1018362 (FI!); P02141757 (P, isopara.!); P03583784 (P, isopara.!)

###### Illustrations.

Wei and Pedley Fl. China 10: 183, t. 210 [6–13] (2010) http://www.plantphoto.cn (in Home Page enter *Calleryabonatiana*).

###### Distribution.

China (Guangxi, Yunnan); Vietnam.

###### Habitat.

tall climber among trees and over shrubs from 200–1000 m.

##### Callerya cochinchinensis

Taxon classificationPlantaeFabalesFabaceae

(Gagnep.) Schot, Blumea 39(1–2): 19 (1994)

≡ Millettiacochinchinensis Gagnep., Notul. Syst. (Paris) 2: 353 (1913). Type: Vietnam, Dong Nai, “Cochinchine, vers Pho-qua, dans la Prov. de Bien-hoa, Mars 1877”, [Jean-Baptiste Louis] Pierres.n.., P02141765 (P, holo.!); P02141766 (P, iso.!); P02141767 (P, iso.!); BM000997331 (BM, iso.!); K000881015 (K, iso.!)

###### Illustration.

Lôc and Vidal in Fl. Cambodge, Laos & Vietnam 30: 38, t. 8 (2001).

###### Distribution.

Vietnam (south).

###### Habitat.

In light scrub and open places along riverbanks, forest margins and ravines from 300 to 1000 m.

##### Callerya cinerea

Taxon classificationPlantaeFabalesFabaceae

(Benth.) Schot, Blumea 39(1–2): 17, t. 2 (1994)

≡ Millettiacinerea Benth. Pl. Jungh. [Miquel] 2: 249 (1852). Type: Bangladesh, “Pongamiacinerea, Sillet TD”, Wallich Cat. 5888A, K000881025 (K, holo.!); Paratypes: Wallich Cat. 5888B, “Pongamiacinerea, Chittagong HB” K000881022 (K!); K000881024 (K, para.!); BM000997333 (BM!); BM000997334 (BM!)
≡ Phaseoloides [Phaseolodes] cinerea (Benth.) Kuntze, Revis. Gen Pl. 1: 201 (1891)
= Millettiabracteosa Gagnep., Not. Syst. 2(11): 352 (1913). Type: China, Yunnan, “bois vers Tchen-fong-shan [Feng shan], August 1894”, [Jean, Marie] Delavay s.n.., P02141760 (P, lecto.! designated here); P02141761 (P, isolecto.!); P02141762 (P, isolecto.!).
= Millettiaheterocarpa Chun ex T.C.Chen, Acta Phytotax. Sin. 3: 364 (1955). Type: China, Guangdong, Heitan Jiao, 8 August 1930, *Nian Qi Chen* 41503, IBSC000908 (IBSC, holo.) ≡ Millettiadielsianavar.heterocarpa (Chun ex T.C.Chen) Z.Wei, Acta Phytotax. Sin. 23(4): 289 (1985) ≡ Calleryadielsianavar.heterocarpa (Chun ex T.C.Chen) X.Y.Zhu ex Z.Wei & Pedley, Fl. China 10: 187 (2010).
= Millettiadielsianavar.solida T.C.Chen ex Z.Wei, Acta Phytotax. Sin 23(4): 289 (1985). Type: China, Hunan Qianyang, *Z.T.Li* 3130 (IBSC, holo.) ≡ Calleryadielsianavar.solida (T.C.Chen ex Z.Wei) X.Y.Zhu ex Z.Wei & Pedley, Fl. China 10: 187 (2010).

###### Illustrations.

Lôc and Vidal in Fl. Cambodge, Laos & Vietnam 30: 43 (2001); Lewis in Curtis’s Bot. Mag. n.s. 29 (2): 141, Pl. 732 (2012); Wei and Pedley Fl. China 10: 184, t. 217 [1–2] (2010). http://www.plantthis.com.au/plant-information.asp?gardener=27142&tabview=photos&plantSpot=

###### Distribution.

Bangladesh; Bhutan; China (Jiangxi, Fujian, Guangdong, Guangxi, Guizhou, Hunan, Sichuan, Xizang, Yunnan); India; Myanmar; Nepal; Thailand.

###### Habitat.

In broad-leaved forest margins, ravines, streamsides and thickets from 150 to 1200 m.

##### 8. Serawaia

Taxon classificationPlantaeFabalesFabaceae

J.Compton & Schrire gen. nov.

urn:lsid:ipni.org:names:77198976-1

###### Diagnosis.

This monospecific genus has several autapomorphies compared with other genera within the tribe. It is the only species that has large and very persistent imbricate floral bracts along the inflorescence enclosing the uniquely golden-yellow flowers. *Serawaia* is the only genus in Clade D that has prominent gibbosities below the stipules. Its nearest affinities lie with *Callerya*, *Kanburia* and *Whitfordiodendron* which all have sericeous backs to their standard petals. The back of the standards of *Serawaia* are, however, pubescent but the hairs are not as long as those in *Afgekia*, the other member of Clade D. The wing petals, which are free from the keel, almost equal the length of the keel as in *Kanburia*, *Whitfordiodendron* and *Afgekia* which distinguishes these four genera readily from *Callerya* whose wings are shorter. The ovary in *Serawaia* is only sparsely hairy whereas in all four other genera within Clade D the ovaries are densely sericeous (see figs 4 and 5 in Schot (1994: 33, 34).

###### Type species.

*Serawaiastrobilifera* (Schot) J.Compton & Schrire ≡ *Calleryastrobilifera* Schot.

###### Genus description.

Scandent twining vines scrambling up trees and along river banks to 8 m high. *Stems* very pale grey or white, terete, glabrous. *Leaves* with 5–7 leaflets, evergreen, glabrous, imparipinnate, rachis 7–20 cm long. *Stipules* 5–8 mm long, linear, persistent, arising from above prominent gibbosities. *Stipels* 3–4 mm long, linear, persistent. *Leaflets* 4–14 × 2–7 cm, broadly or narrowly elliptic, glabrous on both surfaces, apex acuminate or cuspidate, margins entire, base rounded to subcordate. *Inflorescence* erect, sometimes leafy few-branched panicles 12–20 cm long, peduncle pale grey, glabrous. *Flowers* 15–21 mm long, emerging from May to August. *Floral bracts* 15–18 × 8–12 mm, persistent, with longitudinal parallel venation, overlapping flower buds in a strobilate inflorescence. *Bracteoles* 6–7 mm long, at top of pedicel, linear, persistent. *Pedicels* 4–6 mm long, glabrous. *Calyx* 3–6 × 4–6 mm campanulate, oblique, pubescent externally, five lobed, teeth distinctly unequal 2–6 mm long, acute, ciliate. *Standard* 15–18 × 11–17 mm, suborbicular, bright lemon or golden yellow, nectar guide yellow, back of standard sparsely pubescent, apex retuse, callosities of boss type. *Wing petals* 12–14 × 4–5 mm, glabrous, subequal to the keel, each semi-pandurate, slightly curved upwards at the apex; completely free from the keel, apex obtuse, basal claw 2–3 mm long. *Keel petals* 11–13 × 4–5 mm, glabrous, united into a falcate, navicular cup, apex obtuse, basal claw 3–4 mm long. *Stamens* diadelphous, nine fused together, the vexillary one free, all curved upwards at apex. *Ovary* sparsely pubescent, style glabrous, 2–3 mm long curved upwards at apex, *stigma* punctate. *Pods* 19–30 × 2–2.5 cm, flat, linear, or obovate, dehiscent, surface shortly hirsute, smooth, brown and hard when dry, subseptate. *Seeds* 2–3, flattened-orbicular, 17 × 17 × 10 mm, hilum central, elliptic 2–3 × 1 mm.

###### Etymology.

named after the Serawai river in west Kalimantan, a tributary of the Kapuas river, where the species was first discovered.

##### Serawaia strobilifera

Taxon classificationPlantaeFabalesFabaceae

(Schot) J.Compton & Schrire comb. nov.

urn:lsid:ipni.org:names:77198996-1

≡ Calleryastrobilifera Schot, Blumea 39(1–2): 32, t. 4 & 5 (1994). Type: Indonesia, Kalimantan, “Borneo west, Serawai distr., Lebang Hara”, 24 November 1924, *Hans Winckler* 350, L0018804 (L, holo.!); E00301097 (E, iso.!)

###### Illustrations.

Schot in Blumea 39(1–2): 33 fig. 4; 34 fig. 5 (1994) (as *Calleryastrobilifera*).

###### Distribution.

Borneo. Indonesia: Kalimantan, (central and east); Malaysia (Sabah).

###### Habitat.

In open sites climbing among trees and scrub on exposed ridges and riverbanks from sea level to 350 m.

##### 9. Whitfordiodendron

Taxon classificationPlantaeFabalesFabaceae

Elmer, Leafl. Philipp. Bot. 2: 689, 743 (1910), emend nov. J.Compton & Schrire

###### Diagnosis.

The four species of *Whitfordiodendron* share several characters with the new genus *Kanburia* but bracteoles are present on the calyx and persistent in *Whitfordiodendron* (vs. absent in *Kanburia*). The keel petals are densely sericeous in *Whitfordiodendron* (vs. glabrous in *Kanburia* and *Callerya*). The pods in *Whitfordiodendron* are inflated and ovoid with a velutinous or pubescent surface (vs. linear, compressed, glabrescent in *Kanburia*). The ovoid seeds in *Whitfordiodendron* may become fused together when there are more than one per pod (vs. lenticular, separate in pod in *Kanburia*). The wing petals are equal in length with the keel petals in *Whitfordiodendron* (vs. shorter in *Callerya*).

###### Type species.

*Whitfordiodendronscandens* Elmer.

###### Genus description.

Scrambling climbers 10–20 (– 40) m tall. *Stems* grey or brown, terete, glabrous or finely grey puberulent. *Leaves* with 3–13 leaflets, evergreen, nitid above, glabrous or sparsely pubescent, imparipinnate, rachis 9–25 cm long. *Stipules* 1–4 mm long, narrowly deltoid, caducous (persistent *W.erianthum*). *Stipels* absent. *Leaflets* large, 4–15 (– 25) x 2–9 (– 12) cm, ovate, narrowly elliptic or obovate, apex acuminate to cuspidate, margins entire, base rounded or obtuse or acute. *Inflorescence* a terminal panicle 5–20 cm long, peduncle sericeous (cauliflorous and glabrescent 20–60 cm long in *W.nieuwenhuisii*). *Flowers* 8–23 mm long, emerging from February – November (May to January *W.nieuwenhuisii*). *Floral bracts* 2–7 mm long, ovate, obovate or elliptic, caducous. *Bracteoles* at base of or on the calyx 2–7 mm long, obovate, acute or acuminate, persistent. *Pedicels* 0.5–2 mm long, pubescent or sericeous. *Calyx* 2–9 × 3–5 mm campanulate, oblique, ferrugineous, golden or silvery pubescent or sericeous externally, five lobed, teeth unequal (0.5 –) 2–4 mm long, acuminate, pubescent on teeth. *Standard* 8–18 × 9–16 mm, suborbicular or elliptic, inner surface greyish pink, white flushed purple, red, maroon or claret, nectar guide yellow or green, back of standard densely red or golden-brown sericeous, apex acute or obtuse. Callosities ridge or boss type. *Wing petals* 8–18 × 2–5 mm, sparsely pubescent or ciliate along lower margin (sericeous at apex in *W.erianthum*), equal in length to the keel, broadly obovate, free from the keel, apex obtuse, basal claws 2–4 mm long. *Keel petals* 8–10 × 3–5 mm, sericeous externally especially along lower margin, obovate, claw 2–4 mm long, apex obtuse. *Stamens* diadelphous, nine fused together, the vexillary one free, all curved upwards at apex. *Ovary* sericeous, style 2–4 mm long, ciliate, curved upwards at apex, *stigma* punctate. *Pods* 4–10 × 2–5 cm, inflated, ovate or obovate, with two thickened margins either side of suture on both sides of pod, dehiscent, surface rugose or ruminate or sparsely pubescent or pale brown velutinous, subseptate. *Seeds* 1–3, broadly ellipsoid or ovoid, 12–45 × 14–35 × 8–30 mm (often fused together when more than one), hilum central, broadly elliptic 3–5 × 1–2 mm.

###### Distribution.

Brunei; Indonesia (Sumatra, Borneo: west Kalimantan); Malaysia (Peninsula, Borneo: Sabah, Sarawak); Philippines.

###### Etymology.

*Whitfordiodendron* for Whitford and dendron = tree (Gk). The genus commemorates Harry Nichols Whitford (1872–1941) world authority on the economics of rubber and on the native forests of the Philippines.

#### Key to species of *Whitfordiodendron*

**Table:** 

1	Panicles emerging directly from the main trunk (cauliflorous)	*** W. nieuwenhuisii ***
–	Panicles terminal on branches	**2**
2	Flowers 20–23 mm long; calyx 8–9 mm long; stipules persistent	*** W. erianthum ***
–	Flowers 11–15 mm long; calyx 3–4 mm long; stipules caducous	**3**
3	Flowers 11–13 mm long; stipules 3–4 mm long	*** W. sumatranum ***
–	Flowers 13–15 mm long; stipules 1–2 mm	*** W. scandens ***

##### Whitfordiodendron scandens

Taxon classificationPlantaeFabalesFabaceae

Elmer, Leafl. Philipp. Bot. 2: 689–691, 743 (1910)

###### Type.

Philippines, Sibuyan Island, Capiz Province [Romblon], Magellanes [Magdiwang] Mt. Giting-Giting [Guiting-Guiting] April 1910, [Adolph Daniel Edward] *Elmer 12259*, K000880985 (K, lecto.! designated here; holotype PNH destroyed see note below); A00063379 (A, isolecto.!); BM000997328 (BM, isolecto.!); BO-1246846 (BO, isolecto.!); E00683153 (E, isolecto.!); E00683253 (E, isolecto!); GH00052081 (GH, isolecto!); MO-022334 (MO, isolecto.!); U0226578 (U, isolecto.!); US00003630 (US, isolecto.!); P03347973 (P, isolecto.!) ≡ *Adinobotrysscandens* (Elmer) Dunn, Bull. Misc. Inform. Kew 1912(8): 365 (1912) ≡ *Calleryascandens* (Elmer) Schot, Blumea 39(1–2): 31 (1994).

###### Note.

The holotype deposited by Elmer in PNH was destroyed by fire during World War II (T. Circle pers. comm.).

###### Illustrations.

http://www.phytoimages.siu.edu/imgs/pelserpb/r/Fabaceae_Callerya_scandens_43453.html

###### Distribution.

Philippine Islands (Mindanao, Palawan, Panay, Sibuyan).

###### Habitat.

Climbing among lowland forest margins and in thickets from sea level to 200 m.

##### Whitfordiodendron nieuwenhuisii

Taxon classificationPlantaeFabalesFabaceae

(J.J.Sm.) Dunn, Bull. Misc. Inform. Kew 1912(8): 364 (1912b)

≡ Millettianieuwenhuisii J.J.Sm., Bull. Dépt. Agric. Indes Néerl. 3: 17 (1906). Type: Indonesia, Kalimantan, [Borneo], Bloe-oe [Bluu river], [cult.] Buitenzorg, Java, 1897–1898, [Anton Willem] Nieuwenhuis 1294 (BO, holo.)
≡ Adinobotrysnieuwenhuisii (J.J.Sm.) Dunn, Bull. Misc. Inform. Kew 1911(4): 196 (1911) ≡ Calleryanieuwenhuisii (J.J.Sm.) Schot, Blumea 39(1–2): 26 (1994)
= Adinobotrysmyrianthus Dunn, Bull. Misc. Inform. Kew 1911(4): 196 (1911). Type: “Sarawak, 1865–1868”, [Odoardo] Beccari 875, K000881001 (K, lecto.! designated here); S08-15160 (S, isolecto.); P03659572 (P, isolecto.!); M-0233444 (M, isolecto.!) ≡ Whitfordiodendronmyrianthum (Dunn) Dunn, Bull. Misc. Inform. Kew 1912(8): 364 (1912).
= Millettiacuspidata Ridl., Bull. Misc. Inform. Kew 1929(8): 254 (1929). Type: “Sarawak, Matang woods, flowers pinkish”, January 1915, [Henry Nicholas] Ridleys.n.., K000880995 (K, holo.!).

###### Illustration.

http://www.asianplant.net/Fabaceae/Callerya_nieuwenhuisii.htm

###### Distribution.

Brunei; Indonesia (Borneo: Kalimantan); Malaysia (Borneo: Sarawak, Sabah).

###### Habitat.

Climbing near rivers or on steep slopes in evergreen forest from sea level to 1300 m.

##### Whitfordiodendron erianthum

Taxon classificationPlantaeFabalesFabaceae

(Benth.) Dunn, Bull. Misc. Inform. Kew 1912(8): 364 (1912b)

≡ Millettiaeriantha Benth. Pl. Jungh. [Miquel] 2: 250 (1852). Type: “Malacca, Griffith” Herb. East India Company, 1836, received RBG Kew 1861–1862, Herb. [William] Griffith, K000881008 (K, lecto.! designated by Schot (1994)); K000881006 (K, isolecto.!); K000881007 (K, isolecto.!); BM000997329 (BM, isolecto.!); P02753485 (P, isolecto.!)
≡ Phaseoloides [Phaseolodes] eriantha (Benth.) Kuntze, Revis. Gen Pl. 1: 201 (1891)
≡ Adinobotryserianthus (Benth.) Dunn, Bull. Misc. Inform. Kew 1911(4): 196 (1911)
≡ Padbruggeaeriantha (Benth.) Craib, Fl. Siam. 1: 397 (1928)
≡ Calleryaeriantha (Benth.) Schot, Blumea 39(1–2): 21 (1994)

###### Illustration.

https://singapore.biodiversity.online (in Home Page enter *Calleryaeriantha*).

###### Distribution.

Brunei; Indonesia (Borneo: Kalimantan and Sumatra); Malaysia (Peninsula, Borneo: Sabah, Sarawak).

###### Habitat.

In rain forest and along wooded cliffs from sea level to 600 m.

##### Whitfordiodendron sumatranum

Taxon classificationPlantaeFabalesFabaceae

Merr., Pap. Michigan Acad. Sci. 19: 159 (1934).

≡ Calleryasumatrana (Benth.) Schot, Blumea 39(1–2): 35 (1994)

###### Type.

Indonesia, Sumatra, east coast, Boenoet [Bunut], Asahan, 4 January 1925, [Harry Stanley] *Yates* 1261, MICH1104344 (MICH, lecto.! designated here); K000496724 (K, isolecto.!); A00063378 (A, isolecto.!), BO-1246847 (BO, isolecto.!); P03347972 (P, isolecto.!); US00344738 (US, isolecto.!); US00997123 (US, isolecto.!)

###### Distribution.

Indonesia (Sumatra).

###### Habitat.

In lowland forest from sea level to 100 m.

##### 10. Kanburia

Taxon classificationPlantaeFabalesFabaceae

J.Compton, Mattapha, Sirich. & Schrire gen. nov.

urn:lsid:ipni.org:names:77198977-1

###### Diagnosis.

The two species of *Kanburia* share some characters with *Whitfordiodendron*, notably sericeous standards with narrow ridge callosities. In *Kanburia* bracteoles are absent (vs. present in *Whitfordiodendron*), keel petals are glabrous (vs. densely sericeous), pods linear, compressed, 1–1.8 cm wide (vs. inflated, ovoid, 2–2.5 cm wide). *Kanburia* also shares some characters with *Calleryas.str.* but *Kanburia* lacks bracteoles, the flowers are much smaller, 1–1.4 cm long (vs. 1.6–2.8 cm), the wings equal the keel in length (vs. much shorter than keel) and the style is shorter, 1–3 mm long (vs. 6–9 mm long). Molecular evidence for the segregation of this genus is compelling (see figs 3 and 4 in Sirichamorn et al. (2016: 45, 46, 48).

###### Type species.

*Kanburiachlorantha* (Mattapha & Sirich.) J.Compton, Mattapha, Sirich. & Schrire ≡ *Calleryachlorantha* Mattapha & Sirich.

###### Genus description.

Robust, twining woody vines. *Stems* pubescent when young, terete. *Leaves* evergreen, chartaceous and glabrescent (pubescent in *K.tenasserimensis*) when young, sparsely pubescent or glabrous when mature, imparipinnate with 5 leaflets, rachis 2.5–6 cm long. *Stipules* 1–4.5 mm long, acicular or ovate, caducous. *Stipels*1–2 mm long, linear, persistent. *Leaflets* 5–15 × 2–11 cm, elliptic to ovate, sparsely pubescent or glabrescent above and below especially along veins, apex acute, margins entire, base cordate or cuneate. *Inflorescence* a lax many-flowered, erect or pendulous terminal panicle 20–30 cm long, peduncle thinly pubescent. *Flowers* 10–15 mm long, emerging from June to August (August – October in *K.tenasserimensis*). *Floral bracts* 1–5 mm long, caducous, elliptic to ovate. *Bracteoles* absent. *Pedicels* 2–6 mm long, sericeous. *Calyx* 4–6 mm long, campanulate, brownish green (*K.chlorantha*) or purplish brown (*K.tenasserimensis*), externally densely sericeous, five lobed, lower teeth 1–1.5 mm long, deltoid. *Standard* 8–10 × 8–10 mm, broadly obovate to orbicular, inner surface pale green (*K.chlorantha*) dark purple or maroon (*K.tenasserimensis)*, nectar guide dark green (*K.chlorantha*) or pale yellow, back of standard sericeous, apex acute or emarginate. Callosities of ridge type. *Wing petals* 7–8 × 3 mm, glabrous or with a few scattered hairs, semi-pandurate with basal claws 1–2.5 mm long. *Keel petals* 6–7 × 3–3.5 mm, glabrous, united into a long, navicular cup, apex obtuse. *Stamens* diadelphous, nine fused together, the vexillary one free, all curved upwards at apex. *Ovary* sericeous, style 1–3 mm long, curved upwards at apex, *stigma* punctate. *Pods* 5–13 × 1–1.8 cm, flattened, linear, dehiscent, exocarp surface glabrescent, subseptate. *Seeds* 1–6, 10–12 × 9–11 × 3–5 mm, lenticulate, smooth, dark brown, hilum 1–2 × 0.5–1 mm elliptic.

###### Distribution.

Thailand: Kanchanaburi, Suphan Buri, Tak and Ratchaburi [Changwats]. To be expected along the Tenasserim range between Thailand and Myanmar.

###### Etymology.

The generic name refers to Kanburi, the old Siamese name for Kanchanaburi Province in western Thailand where the type species *K.chlorantha* was discovered.

#### Key to species of *Kanburia*

**Table:** 

1	Floral bracts 3–5 × 1–1.8 mm ± equal to flower buds; flowers whiteish to green, nectar guide on standard green	*** K. chlorantha ***
–	Floral bracts 1–1.5 × 0.5 mm, shorter than flower buds; flowers purplish brown, nectar guide on standard yellow	*** K. tenasserimensis ***

##### Kanburia chlorantha

Taxon classificationPlantaeFabalesFabaceae

(Mattapha & Sirich.) J.Compton, Mattapha, Sirich. & Schrire comb. nov.

urn:lsid:ipni.org:names:77198997-1

≡ Calleryachlorantha Mattapha & Sirich., Phytotaxa 263(1): 44. Type: Thailand, Kanchanaburi, Sai Yok district, 20 June 2014, *Phutthai & Sirichamorn* 2014-1 (BKF, holo.!); (BK, iso.!); (K, iso.!); (KKU, iso.!); (L, iso.!); (QBG, iso.!)

###### Illustration

**s.** Sirichamorn et al. in Phytotaxa 263(1): 45 fig. 2 [A–C]; 46 fig. 3 (2016). Plate 1E.

###### Distribution.

Thailand (Kanchanaburi, Tak).

###### Habitat.

In open sites in lowland thickets and degraded bamboo forest 100 to 200 m.

##### Kanburia tenasserimensis

Taxon classificationPlantaeFabalesFabaceae

(Mattapha & Sirich.) J.Compton, Mattapha, Sirich. & Schrire comb. nov.

urn:lsid:ipni.org:names:77198998-1

≡ Calleryatenasserimensis Mattapha & Sirich., Phytotaxa 263(1): 47. Type: Thailand, Ratchaburi, Suan Phueng district, Khoa Chon (Khao Chan) waterfall, 12 September 2015, *Sirichamorn* 2015-13 (BKF, holo.!); (BK, iso.!); (K, iso.!); (KKU, iso.!); (L, iso.!)

###### Illustrations.

Sirichamorn et al. in Phytotaxa 263(1): 45 fig. 2 [D–F]; 48 fig. 4 (2016). Plate 1F.

###### Distribution.

Thailand (Ratchaburi, Suphan Buri).

###### Habitat.

In open sites in dry deciduous and bamboo forest at 200 to 400 m.

##### 11. Afgekia

Taxon classificationPlantaeFabalesFabaceae

Craib, Bull. Misc. Inform. Kew 1927(9): 376 (1927), emend. nov. J.Compton & Schrire

###### Diagnosis.

Both species of *Afgekia* have two pairs of callosities on the standard petal (vs. one pair in all other genera). Stipules are the longest in the tribe 10–25 mm long (vs. 3–12 mm in *Sarcodum*; 5–10 mm in *Endosamara*). Floral bracts are also the longest 30–45 mm (vs. 6–20 mm in *Sarcodum*). Bracteoles are absent (vs. present in *Padbruggea*). The oblong pods are 6–9 cm long, smooth with a velutinous indumentum (vs. 10–25 mm long, obovate or oblong, coarsely ridged and tomentose in *Padbruggea*). Seeds are flattened ellipsoid or flattened orbicular 8–13 mm thick (vs. ovoid or oblong 15–30 mm thick in *Padbruggea*).

###### Type species.

*Afgekiasericea* Craib.

###### Genus description.

Scrambling climbers to 10–20 m. *Stems* green becoming brown, terete, densely sericeous. *Leaves* with 9–17 leaflets, evergreen, finely sericeous above and densely silvery sericeous below, imparipinnate, rachis 8–25 cm long. *Stipules* 10–25 mm long, linear-lanceolate, persistent. *Stipels* 3–5 mm long, acicular. *Leaflets* 3–8 × 2–3 cm, ovate to elliptic, apex softly mucronate, margins finely ciliate, base cuneate or obtuse. *Inflorescence* an erect leafy raceme 30–70 cm long, peduncle silvery sericeous. *Flowers* 23–25 mm long, emerging from June – November. *Floral bracts* 15–35 mm long, lanceolate, apex attenuate, densely pubescent, deep pink (purple in *A.mahidoliae*), caducous. *Bracteoles* absent. *Pedicels* 7–20 mm long, sericeous. *Calyx* 5–7 × 5 mm campanulate, green, ivory, pinkish or purple and sericeous externally, five lobed, teeth pubescent and long acuminate, upper 2 teeth 4–8 mm long, lower 3 teeth with central tooth longest 15–17 mm, laterals 7–9 mm long. *Standard* 15–28 × 20–25 mm, ovate-elliptic, cream suffused with pale or dark pink or purple, sometimes streaked at base, nectar guide pale or dark yellow or greenish, back of standard densely sericeous, apex acute. Callosities in two series, a small papillate pair near the base beneath a much larger corniculate pair either side of the midline. *Wing petals* 20–25 × 5–7 mm, deep pink or purple, slightly falcate, glabrous except for a ciliate fringe below the apex, more or less equal in length to the keel, obovate, apex acute conjoined over the keel, basal claws 3 mm long, with either one (*A.mahidoliae*) or two auricles (*A.sericea*). *Keel petals* 23–26 × 7–15 mm, white, sericeous externally, broadly navicular, claws 3–8 mm long, apex rounded. *Stamens* diadelphous, nine fused together, the vexillary one free but lying adnate to the others, all curved upwards at apex, glabrous (basally tufted in *A.sericea*). *Ovary* sericeous, style 1–3 mm long, glabrous, (tufted apically in *A.mahidoliae*) curved upwards at apex, *stigma* punctate. *Pods* 6–15 × 3–4 cm, inflated, elliptic or oblong, dehiscent, surface smooth to slightly wrinkled, velutinous, subseptate. *Seeds* 2–3, flattened-ellipsoid or orbicular, 15–25 × 10–14 × 8–13 mm, hilum strap-shaped 15–30 mm long. Plate 2 A–C, E)

###### Etymology.

*Afgekia* commemorates Arther Francis George Kerr (1877–1942), Irish physician and pioneering botanist in Thailand.

#### Key to species of *Afgekia*

**Table:** 

1	Leaves with 9–11 leaflets; standard petals purple; wing petals blue purple with 1 claw at base; style sericeous	*** A. mahidoliae ***
–	Leaves with 13–19 leaflets; standard petals cream and pink; wing petals red-purple with 2 claws at base; style glabrous	*** A. sericea ***

##### Afgekia sericea

Taxon classificationPlantaeFabalesFabaceae

Craib, Bull. Misc. Inform. Kew 1927(9): 377 (1927)

###### Type.

Thailand, “Korat [Nakhon Ratchasima], August 1924, pink flowers, climber, Tua pep chang”, *Anuwat* 4 [also called Phya Anuwat Wanaraks], K000881060 (K, holo.!)

###### Illustrations.

Lôc and Vidal in Fl. Cambodge, Laos & Vietnam 30: 14, t. 2 [10–11] (2001); Sirichamorn, MSc Thesis Pl. 4.2 [A1-A3] (2006). https://florafaunaweb.nparks.gov.sg/special-pages/plant-detail.aspx?id=1301 (Plate 2B, C).

###### Distribution.

Laos; Thailand (Saraburi, Buri Ram, Chaiyaphum, Nakhon Ratchasima, Prachin Buri); Vietnam.

###### Habitat.

Open sites in dry evergreen forest scrambling over shrubs at 200 to 520 m.

##### Afgekia mahidoliae

Taxon classificationPlantaeFabalesFabaceae

B.L.Burtt & Chermsir., Notes Roy. Bot. Gard. Edinburgh 31: 131 (1971)

###### Type.

Thailand, Bankhen, Bangkok cultivated plant from Kanchanaburi, 9 September 1968, *C.Chermsirivathana* 997 (BK, holo.); E00275431 (E, iso.!); E00275432 (E, iso.!)

###### Illustrations.

Lôc and Vidal in Fl. Cambodge, Laos & Vietnam 30: 13 (2001); Sirichamorn, MSc Thesis Pl. 4.2 [B1-B3] (2006). https://florafaunaweb.nparks.gov.sg/Special-Pages/plant-detail.aspx?id=3478 (Plate 2A, E).

###### Distribution.

Thailand (Kanchanaburi).

###### Habitat.

Open sites in tropical forest scrambling among shrubs at 200 to 400 m.

### Clade D – *Padbruggea and Austrocallerya*

(Fig. 1; Suppl. material 1: Figs S1–S6)

#### 12. Padbruggea

Taxon classificationPlantaeFabalesFabaceae

Miq., Fl. Nederl. Ind. 1(1): 150 (1855), emend. nov. J.Compton & Schrire

##### Diagnosis.

*Padbruggea* has robust panicles with the peduncle and lateral axes densely brown velutinous (vs. robust panicles with peduncle and lateral axes finely grey-pubescent in *Austrocallerya*). *Padbruggea* has inflated, 4.5–11 cm wide, oblong or obovoid coarsely ridged fruits (vs. inflated, 3–5.2 cm wide, fusiform, finely ridged or striate and torulose pods in *Austrocallerya*). *Austrocallerya* has arching type callosities on the standard petals vs. large papillate callosities in *Padbruggeafilipes* or ridge type callosities in *P.dasyphylla* and *P.maingayi*. *Padbruggea* is distributed from southern China, IndoChina, Indonesia, Malaysia, Thailand to Myanmar whereas *Austrocallerya* occurs in Australia, New Guinea, and some of the adjacent Pacific islands as far south as New Caledonia and Norfolk Island.

##### Type species.

*Padbruggeadasyphylla* Miq.

##### Genus description.

Scrambling climbers reaching 15–25 m. *Stems* dark green becoming brown, terete, densely brown pubescent when young, glabrescent. *Leaves* with 9–19 leaflets, evergreen, pubescent above and below when young, glabrescent or sparsely pubescent at maturity, imparipinnate, rachis 10–30 cm long. *Stipules* 1–8 mm long, ovate or lanceolate, caducous, pubescent or sericeous externally, glabrous internally. *Stipels* 1–3 mm long, filiform, glabrous or setaceous (absent in *P.filipes*). *Leaflets* 5–12 × 2–3 cm, oblong, ovate or elliptic, apex acute or acuminate, margins glabrous or ciliate, base rounded or obtuse. *Inflorescence* an erect terminal, sometimes leafy or cauliflorous panicle 7–35 cm long, peduncle silvery or brown tomentose. *Flowers* 13–25 mm long, emerging from April – June (July – August *P.filipes*). *Floral bracts* 5–25 mm long, linear-lanceolate, ovate or cupuliform, apex acute to acuminate, densely pubescent externally and internally, margin ciliate, green, (pink or purple in *P.filipes*), caducous. *Bracteoles* 3–6 mm long, narrowly lanceolate, caducous (linear 1 mm long *P.filipes*). *Pedicels* 4–7 mm long, densely pubescent (15–25 mm long, sericeous in *P.filipes*). *Calyx* 4–5 × 5 mm campanulate, green or purple, sericeous externally, glabrous internally, five lobed, teeth acute 1–6 mm long, margins ciliate. *Standard* 14–25 × 14–22 mm, orbicular, inner surface lilac or pinkish, nectar guide yellow, back of standard pubescent, apex emarginate, callosities of ridge type (papillate in *P.filipes*). *Wing petals* 13–20 × 8–11 mm, violet or pinkish, slightly falcate, glabrous, more or less equal in length to the keel, elliptic, apex rounded, basal claw 4–5 mm long. *Keel petals* 10–15 × 3–10 mm, white, densely hairy along lower margin externally (glabrous in *P.filipes*); navicular, claw 3–10 mm long, apex acute or rounded. *Stamens* diadelphous, nine fused together, the vexillary one free, all curved upwards at apex, glabrous. *Ovary* densely pubescent or sericeous, style 3–4 mm long, glabrous, tufted at base, curved upwards at apex, *stigma* punctate. *Pods* 10–25 × 5–11 cm, inflated, obovoid, compressed-cuboid or oblong, dehiscent, surface coarsely ridged to rugose, velutinous, subseptate. *Seeds* 1–2, elliptic-ovoid or prolate-spheroid, 50–80 × 40–45 × 30–45 mm, hilum strap-shaped 16–36 mm long.

#### Key to species of *Padbruggea*

**Table:** 

1	Floral bracts 20 × 10 mm; pedicels 15–25 mm long; callosities papillate; pods obovoid	*** P. filipes ***
–	Floral bracts 4–8 × 1–4 mm ; pedicels 3–15 mm long; callosities ridged; pods oblong	**2**
2	Pedicels 5–15 mm long; floral bracts 8 × 4 mm, linear-lanceolate; bracteoles 1–3 mm long; leaves 9–11 foliolate; leaflets elliptic-oblong, apex acute, sparsely pubescent or glabrescent, margins not revolute	*** P. dasyphylla ***
–	Pedicels 3–4 mm long; floral bracts 4 × 1 mm, linear; bracteoles 3–6 mm long; leaves 13–17 foliolate; leaflets elliptic, apex obtuse, densely brown villose on both surfaces, margins revolute	*** P. maingayi ***

##### Padbruggea dasyphylla

Taxon classificationPlantaeFabalesFabaceae

Miq., Fl. Ned. Ind. 1(1): 150 (1855)

= Millettiaoocarpa Prain ex King, J. Asiat. Soc. Bengal Pt. 2, Nat. Hist. 66(2): 92 (1897). Type: Malaysia, Perak, Batu Togoh 250 ft. “climber, June 1888”, [Leonard] Wray 2141, K000881018 (K, lecto.! designated here); Paratypes: Malaysia, Perak, [Revd. Benedetto] *Scortechini* 429, BM001217299 (BM!); K000881017 “Derris n. sp. Sect Aganope” Herb. Mus. Perak (K!); (CAL, x 2).

###### Type.

Indonesia, Java, “A ki kialys, leguminos, Sallak, Java. *Derristomentella* Bl.” [Blume scripsit] Herb. Lugd. Batav. 908.114-1723, April 1825, *Blume s.n..*, L1978535 (L, lecto.! designated here); Herb. Lugd. Batav. 908.114-1724, L1978536 (L, isolecto.!) ≡ *Millettiadasyphylla* (Miq.) Boerl., Handl. Fl. Ned. Ind. 1(2): 349 (1890) ≡ *Calleryadasyphylla* (Miq.) Schot, Blumea 39(1–2): 20 (1994)

###### Note.

Schot (1994: 20) selected a specimen at K (and a “type” at L), collected by Thomas Horsfield in Java, as the type of *Padbruggeadasyphylla*, however, the thirteen small leaflets per leaf, obtuse apices, revolute margins and dense, brown pubescence suggests this is *Padbruggeamaingayi* (see below) not *P.dasyphylla*. Moreover, Miquel’s protologue only specified as type “Derris?tomentella Blume in Herb. L. B.” with the additional information “Banjoemas [Banyumas], op den Salak, in de bosschen bij Tapos, 1000ft”. A search in the Herbarium at L (G. Thijsse pers. comm.) has uncovered two Blume specimens from Java annotated “*Derristomentella* Bl.”. Carl Ludwig Blume was director of the Dutch East Indies Botanic Garden at Buitenzorg [Bogor] from 1823–1826. He collected these specimens on nearby Mount Salak and around the village of Tapos in April 1825. One of these two specimens is used to lectotypify the name here. The annotation “A ki kialys” may refer to a local name for the plant.

###### Illustration.

Lectotype sheet of *Padbruggeadasyphylla* at (L); L1978535.

###### Distribution.

Indonesia (Borneo: Kalimantan, Java, Sumatra); Malaysia (Peninsula, Sarawak); Thailand.

###### Habitat.

Clambering over shrubs and up trees in open sites in evergreen forest at 50 to 1500 m.

###### Etymology.

*Padbruggea* commemorates Dr Robbert Padbrugge (1687–1691) Governor of Ambon for the Dutch East India Company.

##### Padbruggea maingayi

Taxon classificationPlantaeFabalesFabaceae

(Baker) Dunn, Bull. Misc. Inform. Kew 1911(4): 198 (1911)

≡ Millettiamaingayi Baker, Fl. Brit. India 2: 110 (1879). Type: Singapore, 1867–1868, [Alexander Carroll] Maingay 2757, K000881019 (K, lecto.! designated here; see note below)
≡ Phaseoloidesmaingayi (Baker) Kuntze, Revis. Gen. Pl. 1: 201 (1891)

###### Nomenclatural note.

There is a sheet at Kew, K000881019, with two different collections by Maingay. One has two mature pods, a leaflet and a few scraps of stem, the other has several leaflets and bits of stem. There are two labels attached at the bottom of the sheet; one states “Herbarium A.C.Maingay 2757, Singapore, 1867–1868, apparently a climber, no duplicates of this interesting sp.”. The other has “Herbarium of the late A.C.Maingay 605, Malaya, distributed at the Royal Gardens, Kew, 1871”. There is, however, no indication as to which collection represents *Maingay 2757* and which might be *Maingay 605*. Baker in his protologue mentions “Singapore, Maingay” and described the 15 or more leaflets, rounded at both ends and the oblong, velvety pod traversed with deep longitudinal grooves. Since Baker described both fruit and leaves we have inferred that the left-hand fruiting specimen is Maingay 2757 and thereby have selected it to lectotypify the name.

Dunn (1911: 197) in his key to the species *Padbruggeadasyphylla* and *P.maingayi* stated that *P.dasyphylla* had leaflets with revolute margins and was densely tomentose below whereas *P.maingayi* did not have leaflets with revolute margins and was nearly glabrous below. Our examination of type material of both species has found that the reverse is the case as indicated in our key to the species.

###### Illustration.

http://powo.science.kew.org/taxon/urn:lsid:ipni.org:names:21897-1 (5^th^ image).

###### Distribution.

Indonesia (Java); Malaysia (Peninsula); Singapore.

##### Padbruggea filipes

Taxon classificationPlantaeFabalesFabaceae

(Dunn) Craib, Fl. Siam. 1: 397 (1928)

###### Type.

“China, Yunnan, Szemao [Simao], east mountain forests 6700 ft [1520 m], long climber fls pale purple” presented by Dr A. Henry in 1900, *Henry* 11,610, K000881062 (K, lecto.! designated here); US00003999 (US, isolecto.!); (CAL, isolecto.); MO-022362 (MO, isolecto.!)

#### Key to varieties of *Padbruggeafilipes*

**Table:** 

1	Leaflets chartaceous, silvery pubescent only when young, glabrescent	** var. filipes **
–	Leaflets coriaceous, brown tomentose, persistent	**var.** *t* ***omentosa***

##### Padbruggea filipes var. filipes

Taxon classificationPlantaeFabalesFabaceae

≡ Adinobotrysfilipes Dunn, Bull. Misc. Inform. Kew 1911(4): 195 (1911)
≡ Whitfordiodendronfilipes (Dunn) Dunn, Bull. Misc. Inform. Kew 1912: 364 (1912b)
≡ Afgekiafilipes (Dunn) R.Geesink, Leiden Bot. Ser. 8: 77 (1984)

###### Illustrations.

Lôc and Vidal in Fl. Cambodge, Laos & Vietnam 30: 10, t. 2 [1–9] (2001); Sirichamorn, MSc Thesis Pl. 4.2 [C1–C3] (2006). https://baike.baidu.com (in Home Page enter *Whitfordiodendronfilipes*) (Plate 2H, I).

###### Distribution.

China (Guangxi, Yunnan); Laos; Myanmar; Thailand; Vietnam.

###### Habitat.

In open sites climbing over scrub in thickets on dry forested hillsides at 700 to 1700 m.

##### Padbruggea filipes var. tomentosa

Taxon classificationPlantaeFabalesFabaceae

(Z.Wei) J.Compton, Sirich. & Schrire comb. nov.

urn:lsid:ipni.org:names:77198999-1

≡ Whitfordiodendronfilipesvar.tomentosum Z.Wei, Acta Phytotax. Sin. 27(1): 75 (1989). Type: China, Yunnan, Yan-shan hsien, Bar-garh, alt. 1100 m. in bushes 1 Nov. 1939, C.W.Wang 84801, PE00320036 (PE, holo.!); (IBSC, iso.); (KUN, iso.)
≡ Afgekiafilipesvar.tomentosa (Z.Wei) Y.F.Deng & H.N.Qin, Ann. Bot. Fenn. 42(2): 133 (2005)

###### Distribution.

China (Guangxi, Yunnan).

##### 13. Austrocallerya

Taxon classificationPlantaeFabalesFabaceae

J.Compton & Schrire gen. nov.

urn:lsid:ipni.org:names:77198978-1

≡ MillettiaSect.Austromillettia Dunn, J. Linn. Soc., Bot. 41: 135 (1912a)

###### Note.

Dunn (1912a) recognised the distinctiveness of the Australasian species when he placed all three in his MillettiaSect.Austromillettia Dunn. He noted the single flowers as opposed to flowers in pairs (sometimes more than two branching from the same place on the inflorescence axis in other *Millettia* spp.), and the terete woody nature of the pods (Dunn 1912a: 135, 138, 140).

###### Diagnosis.

**Figure 6.**
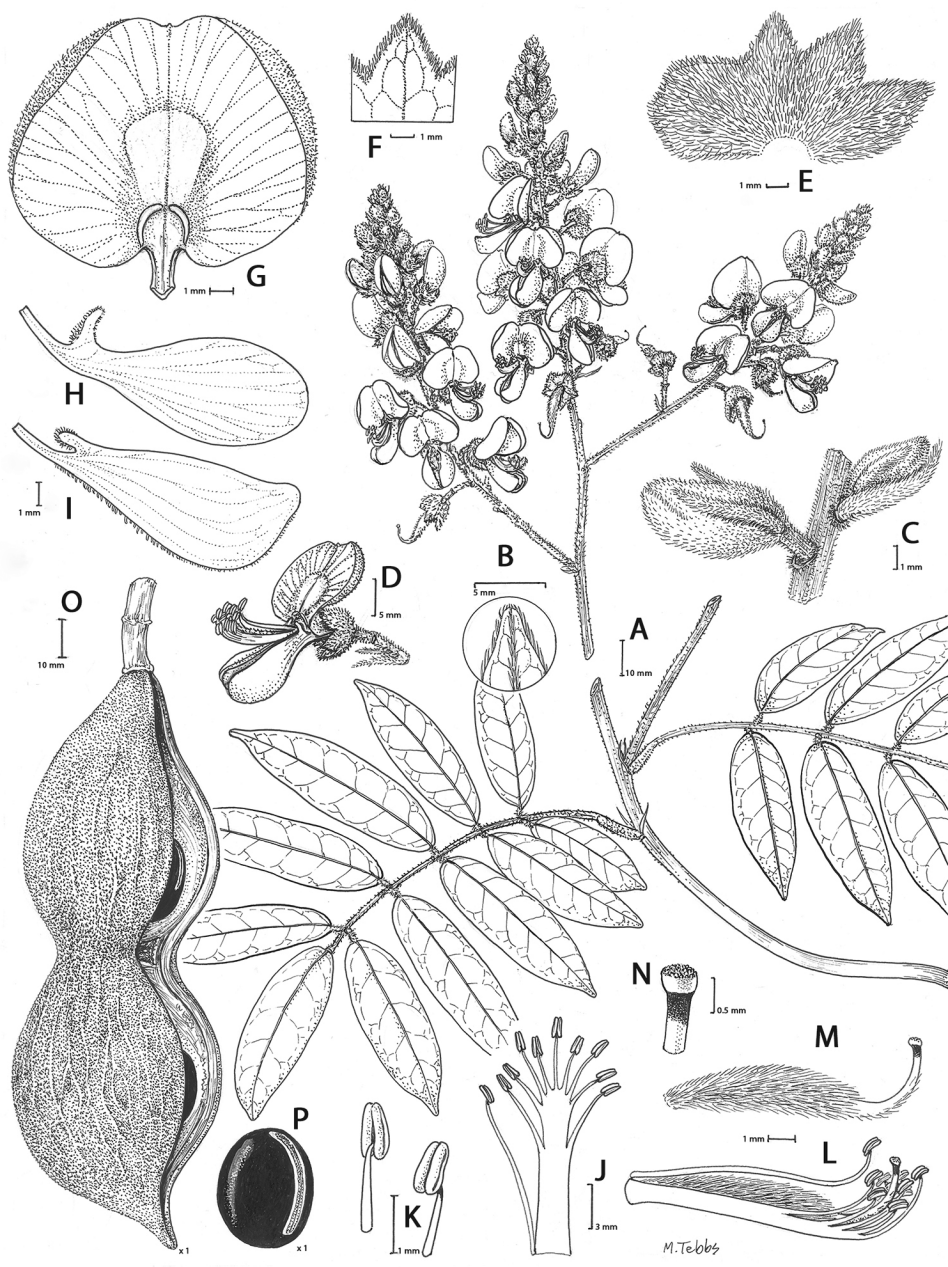
*Austrocalleryaaustralis* (Endl.) J.Compton & Schrire. **A** Habit **B** leaf and detail of leaflet apex **C** flower buds with bract and 2 bracteoles **D** flower **E** calyx external surface **F** calyx detail of inner surface **G** standard petal inner surface **H** wing petal **I** keel petal **J** staminal column ventral view **K** stamens dorsal and ventral views **L** staminal column lateral view **M** ovary lateral view **N** stigma **O** pod **P** seed angled lateral view (all from *Martin* 1392). Drawn by Margaret Tebbs.

*Austrocallerya* comprises three Australasian species with glabrous or finely pubescent young leaves and stems (vs. these densely brown tomentose in *Padbruggea*, see Table 4 and Fig. 6). The robust paniculate inflorescences are more erect than those in *Padbruggea* and the flowers have very broad standard petals with a recessed dividing midline. Either side of the midline is an arch callosity which forms a short crescent arching over the staminal sheath (vs. papillate or ridge callosities in *Padbruggea*). The pods are fusiform (vs. obovoid or compressed-cuboid in *Padbruggea*), torulose and with either longitudinal striations and furrows (*A.megasperma*), or with irregular fine striations (*A.australis*) or smooth (*A.pilipes*), the surface in all cases being densely velutinous or pubescent. The pods of *Austrocallerya* can be distinguished from those of *Padbruggea*, which are also densely velutinous, by their outline. *Padbruggea* pods are either obovoid (in *P.filipes*) or oblong with a prominent dorsal midline flanked by two large flanges meeting at the apex (*P.dasyphylla*). The pods in *Austrocallerya* are 30–52 mm wide (vs. 40–110 mm wide in *Padbruggea*). The 2–10 seeds in *Austrocallerya* are oblong, ellipsoid or globose, frequently with one side compressed within the pod (vs. 1–2 elliptic-ovoid or prolate-spheroid seeds which may also be laterally compressed in *Padbruggea*). In *Austrocallerya* the strap-shaped hila are 16–30 × 2–4 mm, (vs 16–40 × 5–10 mm in *Padbruggea*). Fig. 6.

###### Type species.

*Austrocalleryaaustralis* (Endl.) J.Compton & Schrire ≡ *Pterocarpusaustralis* Endl.

###### Genus description.

Three species of robust, twining woody vines scrambling from 2–20 m in height. *Stems* grey, tan or reddish brown, terete, mature stems and branches exhibiting a flaky peeling bark, young growth sericeous-pubescent becoming glabrous. *Stipules* 1.5–6 mm long, deltoid or filiform, sericeous, caducous (persistent in *A.megasperma*). *Stipels* 1–4 mm long, filiform, sericeous, persistent or caducous (absent in *A.pilipes*). *Leaves* evergreen, coriaceous and nitid when mature, imparipinnate with 5–19 leaflets, rachis 7–25 cm long, pubescent becoming glabrous. *Leaflets* 3–14 × 1–7 cm, elliptic, narrowly oblong or obovate, upper surface glabrous, lower surface with scattered hairs especially along veins, apex obtuse, retuse, emarginate, acute, acuminate or caudate, margins entire, base truncate, obtuse or cuneate. *Inflorescence* a robust many-flowered terminal panicle 6–40 cm long, sericeous or pubescent. *Flowers* 11–16 mm long, emerging from September to December (in *A.australis* from December to April but in New Guinea from June to October). *Floral bracts* 2–7 mm long, (8–15 mm in *A.pilipes*), white, green or pink, sericeous, cupuliform or linear or ovate to narrowly ovate, caducous. *Bracteoles* 1–7 mm, at top of pedicel, linear, sericeous, acuminate, caducous. *Pedicels* 3–21 mm long, pinkish, sericeous or pubescent. *Calyx* 3–7 × 4–10 mm, campanulate, yellowish or purple, glabrous internally, sparsely pubescent or sericeous externally, five lobed, upper teeth acute, 4–5 mm long, lower teeth 3–5 mm long, acuminate. *Standard* 12–22 × 11–18 mm, orbicular or broadly elliptic, whitish, reddish-purple, mauve, lilac, pink or purple, deflexed backwards near the base, apex with a short mucro, lamina veined, nectar guide yellow, greenish yellow or lime green, radiating up the centre of the lamina from the base, back of standard pubescent, callosities arched over the staminal sheath and divided centrally by a linear sinus, each half forming an arch. *Wing petals* 11–14 × 5–6 mm, purple or maroon, equalling keel in length, glabrous, each semi-pandurate with basal claws 2–3 mm long. *Keel petals*11–14 × 4–6 mm, dark reddish, purple or maroon (white with purple apices in *A.megasperma*), glabrous or upper margin ciliate at base (*A.australis*), petals united into a semi-pandurate cup, apex obtuse. Wings and keel petals spreading after anthesis. *Stamens* diadelphous, nine fused together, the vexillary one free, all curved upwards at apex, glabrous. *Ovary* sericeous, style 3–5 mm long, curved upwards at apex, *stigma* punctate. *Pods* 7–23 × 3–5.2 cm, fusiform, inflated, torulose, tardily dehiscent, exocarp finely ridged, longitudinally striate (smooth in *A.pilipes*), surface velutinous, endocarp chartaceous, the seeds in hollow cavities, subseptate. *Seeds* (1 –) 2–6, ellipsoid, broadly ovoid to squarish, smooth, brown or orange-brown 12–43 × 12–42 × 12–41 mm, sometimes compressed laterally inside the pod, hilum 16–30 mm x 2–4 mm, strap-shaped. Fig. 6.

###### Distribution.

Australia (New South Wales, Queensland); Papua New Guinea (Bougainville Island, New Britain Island); New Caledonia; Cook Islands.

###### Habitat.

In rainforest or in dry forest from sea level to 1600 m, climbing up trees and over shrubs.

###### Etymology.

The generic name reflects the southern hemisphere distribution of the genus, austro - “australis” = south (Latin) and “callerya” a reference to their former generic placement and affinity.

#### Key to species of *Austrocallerya*

**Table:** 

1	Floral bracts 2–7 × 0.5–2 mm, linear or narrowly ovate not enclosing flower buds prior to anthesis; bracteoles 1–3 × 0.5–1 mm; pod surface with longitudinal ridges	**2**
–	Floral bracts 8–15 × 8–12 mm, cupuliform, enclosing flower buds prior to anthesis; bracteoles 5–7 × 1–2 mm; pod surface glabrous or finely tesselated	*** A. pilipes ***
2	Floral bracts 6–7 × 2 mm; pod surface deeply ridged or grooved; seeds dark brown	*** A. megasperma ***
–	Floral bracts 2–4 × 0.5–1 mm; pod surface finely and shallowly ribbed; seeds orange-brown	*** A. australis ***

##### Austrocallerya australis

Taxon classificationPlantaeFabalesFabaceae

(Endl.) J.Compton & Schrire comb. nov.

urn:lsid:ipni.org:names:77199026-1

≡ Pterocarpusaustralis Endl., Prodr. Fl. Norf.: 94 (1833). Type: Australia, Norfolk Island, “In Insula Norfolk, Ferd. Bauer”, 1804–1805, F.Bauer s.n.., W0046224 (W, lecto.! designated here); W0046223 (W, isolecto.!); W0046225 (W, isolecto.!); “Norfolk Island, Bauer Hb. Brown” K000880984 (K, isolecto.!)
≡ Millettiaaustralis (Endl.) Benth. Pl. Jungh. [Miquel] 2: 250 (1852)
≡ Wisteriaaustralis (Endl.) F.Muell. Second Systematic Census of Australian Pl.: 68 (1885)
≡ Kraunhiaaustralis (Endl.) Greene, Pittonia 2(10): 175 (1891)
≡ Calleryaaustralis (Endl.) Schot, Blumea 39(1): 16 (1994)
= Millettiamaideniana F.M.Bailey, Queensland Bot. Bull. Agric. 5: 12 (1892). Type: Australia, New South Wales, “Port Macquarie, Bean tree, 16 ins diameter, 25–30 ft high, Nov. 1891,”, J.H.Maiden s.n., BRI-AQ22886 (BRI, holo.!) ≡ Wisteriamaideniana (F.M.Bailey) C.Moore, Handbook Fl. New South Wales: 517 (1893).
= Calleryaneocaledonica I.C.Nielsen & Veillon, Adansonia ser. 3, 27(1): 82 (2005). Type: Nouvelle-Calédonie, Dumbéa, Nakutakoin, western slope of Pic Jacob, c. 150 m, 15 January 1992, *J.M.Veillon* 7466, P00625937 (P, holo.!); P00629538 (P, iso.!); P00625939 (P, iso.!); (AAU, iso.), NOU006199 (NOU, iso.).

###### Illustration.

Harden in Flora of New South Wales 2: 615 (2000). http://www.floragreatlakes.info/html/rfspecies/callerya.html (*Calleryaaustralis*).

###### Distribution.

Australia (Norfolk Island, New South Wales, Queensland); Bougainville Island; Cook Islands; New Britain Island; New Guinea; New Caledonia.

###### Habitat.

In rainforest climbing in thickets on forested hillsides at 300 to 1600 m.

##### Austrocallerya megasperma

Taxon classificationPlantaeFabalesFabaceae

(F.Muell.) J.Compton & Schrire comb. nov.

urn:lsid:ipni.org:names:77199027-1

≡ Wisteriamegasperma F.Muell., Fragmenta Phytographiae Australiae 1: 10 (1858). Type Australia, Queensland, “Ad rivulos sylvaticos fluvii Pine River prope sinum Moreton Bay”, F.von Mueller & W.Hill s.n.., MEL2144485 (MEL, lecto.!, designated by Compton (2016)); MEL2144484 (MEL, isolecto.!)
≡ Millettiamegasperma (F.Muell.) Benth., Fl. Austral. 2: 211 (1864)
≡ Kraunhiamegasperma (F.Muell.) Greene, Pittonia 2(10): 175. (1891)
≡ Phaseoloides [Phaseolodes] megasperma (F.Muell.) Kuntze, Revis. Gen Pl. 1: 201 (1891)
≡ Calleryamegasperma (F.Muell.) Schot, Blumea 39(1): 25 (1994)

###### Illustrations.

Harden in Flora of New South Wales 2: 615 (2000) http://www.floragreatlakes.info/html/rfspecies/callerya.html (*Calleryamegasperma*).

###### Distribution.

Australia (New South Wales, Queensland).

###### Habitat.

In dry forest climbing up trees from sea level to 300 m.

###### Note.

1) Schot (1994: 25) chose a specimen at K collected at Richmond River, NSW by Charles Moore, K000880988 as the type, but this was not cited in the protologue nor was it from the type locality and is therefore not original material. Another specimen K000880989 collected by Mueller and Hill from “Upper Brisbane River” was also collected from a location not cited in the protologue. We have chosen MEL2144485 collected by Mueller and Hill from the Pine River near Moreton Bay as cited in the protologue as lectotype.

2) *Wisteriainvoluta* Sprague (Sprague 1904: 141) was described from cultivated material at K. The material was collected from the Richmond River area of NSW, Australia, collector unknown. Sprague (1905: 3) recombined the species as *Derrisinvoluta* (Sprague) Sprague the following year having seen the flat, one-seeded, winged fruits of the millettioid genus *Derris* Lour.

##### Austrocallerya pilipes

Taxon classificationPlantaeFabalesFabaceae

(F.M.Bailey) J.Compton & Schrire comb. nov.

urn:lsid:ipni.org:names:77199028-1

≡ Millettiapilipes F.M.Bailey, Second Addenda to Third Supplement of the Synopsis of the Queensland Flora 108 (1890) [published with Catalogue of the Indigenous and Naturalised Plants of Queensland]. Type: Australia, Queensland, Cook pastoral district, “Johnstone River, Dr. Thos. L. Bancroft, large climber”, 1885–1886, *T.L.Bancroft*s.n.., BRI-AQ0022887 (BRI, holo.!); BM000810924 (BM, iso.!)
≡ Wisteriapilipes (F.M.Bailey) Sprague, Gard. Chron. Ser. 3., 36: 141 (1904)

###### Note.

There is also a specimen at K collected by Frederick M. Bailey from the Johnstone River in August 1892 – K000880982. Bailey, the author of the name, had been Colonial Botanist for Queensland since 1881 but Schot’s choice of this specimen as an isotype (Schot, 1994: 29) is incorrect as the holotype cited in the protologue was collected by Thomas Lane Bancroft, moreover, Bailey’s specimen was collected two years after the protologue was published.

###### Illustrations.

Cooper, Australian Rainforest Fruits, a Field Guide: 175 (2013). http://keys.trin.org.au/key-server/data/0e0f0504-0103-430d-8004-060d07080d04/media/Html/taxon/Callerya_pilipes.htm

###### Distribution.

Australia (Queensland).

###### Habitat.

In rainforest climbing trees and over scrub from 300 to 1200 m.

### Clade E – *Wisteria*

(Fig. 1; Suppl. material 1: Figs S1–S6)

#### 14. Wisteria

Taxon classificationPlantaeFabalesFabaceae

Nutt., Gen. Amer. Pl. 2: 115 (1818), emend. nov. J.Compton & Schrire

##### Diagnosis.

In *Wisteria* the wings remain adnate to the keel after anthesis (vs. separated from the keel in *Wisteriopsis*). The Asian species all have papillate callosities similar to those in *Padbruggeafilipes* but the North American *W.frutescens* has small ridge type callosities. The pods of all Asian species are gently torulose with a velutinous surface (striated, ridged, furrowed or tessellated in *Padruggea* and *Austrocallerya*). The North American *W.frutescens* has straight, smooth, glabrous pods.

##### Type species.

*Wisteriafrutescens* (L.) Poir. ≡ *Glycinefrutescens* L.,

##### Genus description.

Robust, twining woody vines to more than 30 m in height. *Stems* green and pubescent when young, becoming grey or reddish brown at maturity, terete. *Stipules* 4–8 mm long, linear, sericeous, caducous. *Stipels* 3–4 mm long, filiform, sericeous, caducous. *Leaves* deciduous, chartaceous and villose when young, glabrous when mature, imparipinnate with (7 –)9–13(–15) leaflets, rachis 4–7 cm long, pubescent becoming glabrous. *Leaflets* 2–10 × 1–5 cm, ovate-elliptic or elliptic-lanceolate, upper surface glabrous (very finely pubescent in *W.frutescens*), lower surface with scattered hairs along veins, apex acute, acuminate or shortly caudate, margins entire or gently sinuate, base obtuse or cuneate. *Inflorescence* a terminal spreading to pendulous raceme 12–30(– 140) cm long, peduncle villose or pubescent. *Flowers* 15–30 mm long, emerging from April to May (June to August in *W.frutescens*). *Floral bracts* 5–15(– 23) mm long, brown or silvery chartaceous, linear or cupuliform, attenuate or caudate, caducous. *Bracteoles* 2–4 mm, (absent in *W.frutescens*) at top of pedicel, linear, acuminate, caducous. *Pedicels* 5–50 mm long, pubescent. *Calyx* 4–10 × 4–6 mm, campanulate or tubular, sparsely to densely pubescent or sericeous externally (sometimes with glandular hairs *W.frutescens*), five toothed, upper teeth acute, 3–5 mm long, lower teeth 3–6 mm long, (central tooth – 8 mm) acuminate, green or white. *Standard* 17–28 × 19–28 mm, suborbicular, lilac or pale purple, deflexed near the base (deflexed near the middle in *W.frutescens*), apex with a short mucro or retuse, nectar guide yellow, back of standard glabrous or sparsely pubescent along margin, callosities papillate either side of the midline at base (of ridge type in *W.frutescens*). *Wing petals* 12–20 × 5–8 mm, lilac or purple, equalling keel or slightly longer, glabrous, each semi-pandurate with basal claws 2–4 mm long (6–8 mm in *W.brachybotrys*). *Keel petals* 11–18 × 4–8 mm, lilac or purple, glabrous, united into a semi-pandurate cup, apex obtuse. *Stamens* diadelphous, nine fused together, the vexillary one free, all curved upwards at apex, glabrous. *Ovary* sericeous, style 3–5 mm long, curved upwards at apex, *stigma* punctate. *Pods* 10–24 × 1.2–3 cm, compressed, slightly torulose, (straight in *W.frutescens*) tardily dehiscent, exocarp smooth, surface densely velutinous, endocarp pithy, the seeds in shallow cavities, subseptate. *Seeds* (1 –)3–6(– 8), lenticular, (reniform-cuboid in *W.frutescens*) smooth, pale or dark brown, 8–10 × 8–12 × 2–4 mm, (8–10 × 4–6 × 4–6 mm in *W.frutescens*), hilum 1–2 × 1–3 mm, elliptic. Plate 3F–G.

##### Distribution.

China (Anhui, Fujian, Guangxi, Hebei, Henan, Hunan, Jiangsu, Jiangxi, Shaanxi, Shandong, Shangxi, Zhejiang); Japan; Korea; east North America.

##### Habitat.

In temperate forests from sea level to 1800 m, climbing among trees and shrubs.

##### Etymology.

The generic name commemorates the anatomist Professor Caspar Wistar (1761–1818), President of the American Philosphical Society. It also commemorates Caspar Wistar’s cousin Charles Jones Wister (1782–1865), friend of Thomas Nuttal who was the author of the name.

#### Key to species of *Wisteria*

**Table:** 

1	Inflorescence cylindrical in bud; bracteoles present below calyx; papillate callosities present on standard petals; pods velutinous, slightly torulose; seeds lenticular	**2**
–	Inflorescence conical in bud; bracteoles absent; standard petal with ridge callosities; wing petals with subulate spur-like auricles 4–6 mm long; pods glabrous with a few longitudinal undulations; seeds reniform or oblong	*** W. frutescens ***
2	Inflorescence 12–120 cm long; floral bracts 5–20 × 2–12 mm, linear or ovate-lanceolate, standard petal with papillate callosities; wing petals with short deltoid auricles 1–3 mm long; pods velutinous; seeds lenticular	**3**
–	Inflorescence 8–20 cm long; floral bracts 15–23 × 5–12 mm, broadly ovate, attenuate, densely sericeous	*** W. brachybotrys ***
3	Inflorescence 18–120 cm long; floral bracts 5–12 × 2–6 mm, linear, puberulent; standard petal broadly ovate, 8–16 × 8–12 mm	*** W. floribunda ***
–	Inflorescence 12–20 cm long; floral bracts 8–20 × 10–12 mm, ovate-lanceolate, puberulent; standard petal suborbicular, 20–30 × 20–30 mm	*** W. sinensis ***

##### Wisteria frutescens

Taxon classificationPlantaeFabalesFabaceae

(L.) Poir., Tab. Encycl. 3: 674 (1823)

###### Type.

[icon] “*Phaseoloides, frutescens Caroliniana foliis pinnatis floribus caeruleis conglomeratis* Carolina Kidney-Bean” in Miller Cat. Pl.: t. 15 (1730), (lecto.!, designated by Reveal (1997)).

#### Key to subspecies of *Wisteriafrutescens*

**Table:** 

1	Inflorescence 8–15 cm long; pedicel and calyx with simple hairs; calyx teeth subequal, all ± acute	** subsp. frutescens **
–	Inflorescence (8 –)10–30 cm long; pedicel and calyx covered externally with simple and clavate glandular hairs; upper lip of calyx with teeth acute, lower lip longer, teeth acuminate	** subsp. macrostachya **

##### Wisteria frutescens subsp. frutescens

Taxon classificationPlantaeFabalesFabaceae

≡ Glycinefrutescens L., Sp. Pl. 1(2): 753 (1753)
≡ Kraunhiafrutescens (L.) Raf., Med. Repos. Original Essays Intelligence Phys. [hex.] 2 [vol.] 5: 352 (1808) nom. rej. (Art. 56, McNeil & al., 2012)
≡ Apiosfrutescens (L.) Pursh, Fl. Amer. Sept. 2: 474 (1814)
≡ Thyrsanthusfrutescens (L.) Elliott, J. Acad. Nat. Sci. Philadelphia 1: 371 (1818)
≡ Phaseolusfrutescens (L.) Eaton & Wright, N. Am. Bot. ed 8: 354 (1840)
≡ Kraunhiafrutescens (L.) Greene, Pittonia 2: 175 (1891)
≡ Phaseoloides [Phaseolodes] frutescens (L.) Kuntze, Revis Gen. Pl. 1: 201 (1891)
≡ Bradlea [as Bradleya] frutescens (L.) Britton, Man. Fl. N. States & Canada 549 (1901). Note: Bradleya is treated as a homonym of Bradlea Adans. under Art. 53.2 (Turland et al. 2018)
= Glycinecaerulea Salisb., Prodromus stirpium in horto Chapel Allerton vigentium 335 (1796) [G.frutescens L. was cited].
= Wisteriaspeciosa Nutt., Gen. N. Amer. Pl. 2: 116 (1818) [G.frutescens L. was cited].

###### Illustrations.

Compton in Curtis’s Bot. Mag. 32 (3–4): 234, t. 2 & 3 (2015a) https://gobotany.newenglandwild.org/species/wisteria/frutescens/; (Plate 3F); Compton and Lane (2019: 174).

###### Distribution.

USA (Connecticut, Delaware, Illinois, Indiana, Iowa, Maryland, Massachusetts, Michigan, Missouri, New Jersey, New York, North Carolina, Ohio, Pennsylvania, Rhode Island, Virginia, West Virginia).

###### Habitat.

In clearings of evergreen lowland forest and along riverbanks at sea level to 650 m.

###### Note.

Plants from more southerly regions either side of the Appalachian mountains have previously been recognised as a separate species *Wisteriamacrostachya* (Nutt. ex Torr. & A.Gray) B.L.Rob. & Fernald, Gray Man. Bot. N. U.S. ed. 7: 515. 1908. Observation of living plants and careful examination of many herbarium specimens coupled with the DNA generated results from this study have led us to conclude that there is only the single species *W.frutescens* representing the genus *Wisteria* in North America. We also conclude that there is sufficient difference between the southern plants and those from further north to recognise the southern one at the rank of subspecies. At the rank of species the name *Diplonyxelegans* Raf. (1817: 101) has priority over the widely used name *Wisteriamacrostachya* (Robinson and Fernald 1908: 515), however, the combination *Wisteriaelegans* has never been made (for a more comprehensive discussion see Compton (2015a)). Plants from the northerly range of the species (subsp.frutescens) have smaller and shorter inflorescences without (or with very few) glandular hairs on pedicels and calyces (vs pedicels and calyces covered in clavate glandular hairs in subsp.macrostachya). The teeth on the calyces of subsp.frutescens are ± subequal (vs lower teeth much longer in subsp.macrostachya). Racemes from the colder north (subsp.frutescens) are usually considerably shorter than the elongating racemes of southerly plants (subsp.macrostachya). For a more comprehensive description of these taxa see Compton and Lane (2019).

##### Wisteria frutescens subsp. macrostachya

Taxon classificationPlantaeFabalesFabaceae

(Nutt. ex Torr. & A.Gray) J.Compton & Schrire comb. nov.

urn:lsid:ipni.org:names:77199029-1

≡ Wisteriafrutescensvar.macrostachya Nutt. ex Torr. & A.Gray, Fl. N. Am. 1(2): 283 (1838). Type: U.S.A. Louisiana, [Louis François] Tainturier [s.n..] (fide Nuttall), PH00029452 (PH, holo.!)
≡ Wisteriamacrostachya (Nutt. ex Torr. & A.Gray) B.L.Rob. & Fernald, Gray Man. Bot. N. U.S. ed. 7: 515 (1908) [as W.macrostachys]
≡ Kraunhiamacrostachya (Nutt. ex Torr. & A. Gray) Small, Bull. Torrey Bot. Club 25: 134–135 (1898) [as K.macrostachys];
≡ Bradlea [as Bradleyamacrostachys] macrostachya (Nutt. ex Torr. & A.Gray) Britton, Man. Fl. N. States & Canada 549 (1901). Note: Bradleya is treated as a homonym of Bradlea under Art. 53.2 ex 9 (Turland et al. 2018)
= Diplonyxelegans Raf., Fl. Ludov. 101–102 (1817). Type: Louisiana, Alexandria, J.Hale s.n.., P00680371 (P, neo.!, designated by Compton (2015a)).
= Thyrsanthusfloridana Croom, Amer. Journ. Sci. and Arts 25: 75 (1834). Type: “Wisteriaspeciosa, Thyrsanthusfrutescens Ell., river banks Florida”, Chapman Herbarium [collector unknown], GH00429033 (GH, neo.!, designated by Compton (2015a)).
= Glycinefrutescensvar.magnifica Hérincq, L’Horticulteur Français de mil huit cent cinquante et un 5: 220 (1855). Type: [Icon] L’Horticulteur Français de mil huit cent cinquante et un 5: 220 (1855), (lecto.!, designated by Compton (2015a)
≡ Wisteriafrutescensvar.magnifica (Hérincq) André, Revue Horticole 1862: 50 (1862).

###### Illustrations.

Compton in Curtis’s Bot. Mag. 32 (3–4): 237, t. 4 & 5 (2015a).

###### Distribution.

USA (Alabama, Arkansas, Florida, Georgia, Kentucky, Louisiana, Mississippi, Oklahoma, South Carolina, Tennessee, Texas).

##### Wisteria brachybotrys

Taxon classificationPlantaeFabalesFabaceae

Siebold & Zucc., Fl. Jap. 1: 92. t. 45. (1839)

= Wisteriavenusta Rehder & E. H. Wilson, Publ. Arnold Arbor. 4 vol. 2: 514 (1916). Type: Japan, Hondo [Honshu], Musashi, [cultivated] Iris Garden, Kamata, 27 April 1914, *E.H.Wilson 6580*, US00003998 (US, lecto.!, designated by Compton and Lack (2012)); (GH, isolecto.!); K (as *E.H.Wilson 6580*, but dated 6 May 1914)
≡ Rehsoniavenusta (Rehder & E.H.Wilson) Stritch, Phytologia 56(3): 183 (1984).

###### Type.

Japan, “1829, *Herb. de Siebold*” [Fl. Jap.: t. 45.], L0176059 (L, lecto.!, designated by Compton and Lack (2012)); “Japonia coll. Dr. von Siebold donné par Mr Blume” P02942362 (P, isolecto.!) ≡ *Phaseoloides* [*Phaseolodes*] *brachybotrys* (Siebold & Zucc.) Kuntze, Revis. Gen. Pl. 1: 202 (1891) ≡ Millettiafloribundavar.brachybotrys (Siebold & Zucc.) Matsum., Bot. Mag. (Tokyo) 16: 64 (1902) ≡ Kraunhiasinensisvar.brachybotrys (Siebold & Zucc.) Makino, Bot. Mag. (Tokyo) 24: 76 (1910) ≡ Kraunhiafloribundavar.brachybotrys (Siebold & Zucc.) Makino in Bot. Mag. (Tokyo) 25: 18 (1911) ≡ *Rehsoniabrachybotrys* (Siebold & Zucc.) Stritch, Phytologia 56(3): 184 (1984)

###### Illustrations.

Compton in Curtis’s Bot. Mag. 32 (3–4): 305, Pl. 816 & 817, t. 1 & 2 (2015d); Wei and Pedley, Fl. China 10: 189, t. 223 [8] (2010). Plate 3G.

###### Distribution.

Japan (Honshu, Kyushu, Shikoku).

###### Habitat.

Climbing over trees and shrubs in mixed evergreen and deciduous forest and along riverbanks at sea level to 1000 m.

##### Wisteria floribunda

Taxon classificationPlantaeFabalesFabaceae

(Willd) DC., Prodr. 2: 390 (1825), nom. cons. (Taxon 61(4): 882)

≡ Glycinefloribunda Willd. Sp. Pl. 3(2): 1066 (1802). Type: Japan, Aoyama, Suma Kobe city, 10 May 1967, *M.Hotta 16502* (K, neo.!, designated by Compton and Lack (2012)); (P, isoneo.!); (L, isoneo.!); (E, isoneo.!)
≡ Phaseoloides [Phaseolodes] floribunda (Willd.) Kuntze, Revis. Gen. Pl. 1: 202 (1891)
≡ Kraunhiafloribunda (Willd.) Taub., Engler & Prantl, Nat. Pflanzenfam. 3(3): 271. (1894)
≡ Millettiafloribunda (Willd.) Matsum., Bot. Mag. (Tokyo) 16: 64 (1902)
≡ Rehsoniafloribunda (Willd.) Stritch in Phytologia 56: 183 (1984)
= Wisteriamacrobotrys Siebold ex Lemoine, Catalogue et Prix Courant no. 56: 5 (1869). Type: [Icon] *Neubert Deutsches Gartenmagazin für Garten und Blumenkunde* 23: 17 (1870), (lecto.!, designated by Compton and Thijsse (2015))
≡ Wisteriasinensisvar.macrobotrys (Siebold ex Lemoine) Lavallée, Énum. Arbres: 65 (1877) ≡ Wisteriafloribundaf.macrobotrys (Siebold ex Lemoine) Rehder & E.H.Wilson, Publ. Arnold Arb. 4: 513 (1916)
≡ Kraunhiafloribundavar.macrobotrys (Siebold ex Lemoine) Nash, Journal of the New York Botanical Garden 20: 14 (1919)
≡ Wisteriafloribundavar.macrobotrys (Siebold ex Lemoine) L.H.Bailey, Manual of Cultivated Plants ed. 1: 417 (1923).
= Wisteriamultijuga Van Houtte, Fl. Serres Jard. Eur. 19: 125 (1874). Type: [Icon] Fl. Serres Jard. Eur. 19: 125 (1874) ≡ Glycinemultijuga (Van Houtte) Clémenc., Revue Horticole 46: 300 (1874), (lecto.!, designated by Compton and Lack (2012))
≡ Wisteriasinensisvar.multijuga (Van Houtte) H.Jaeger & Beissn., Die Zierghölze der Gärten und Parkanlagen 425 (1889) ≡ Wisteriapolystachiosf.multijuga (Van Houtte) Beissn. Schelle & Zabel, Handbuch der Laubholz-Benennung 269 (1903).

###### Illustrations.

Siebold and Zuccarini in Flora Japonica 1: t. 44 (1839) as *Wisteriachinensis*; Compton and Thijsse in Curtis’s Bot. Mag. 32 (3–4): 350, Pl. 818, t. 2 & 3 (2015).

###### Distribution.

Korea; Japan (Honshu, Kyushu, Shikoku).

###### Habitat.

Climbing over trees and shrubs in mixed evergreen and deciduous forest and in thickets at sea level to 1500 m.

##### Wisteria sinensis

Taxon classificationPlantaeFabalesFabaceae

(Sims) DC. Prodr. 2: 390 (1825) ≡ Glycine sinensis Sims

###### Type.

[Icon] Bot. Mag. 46 [n.s.4] t. 2083 (1819), (lecto.!, designated by Compton (2015b)).

#### Key to varieties of *Wisteriasinensis*

**Table:** 

1	Lower lip of calyx with teeth 1–2mm long, subequal, upper surface of leaves finely reticulate	** var. brevidentata **
–	Lower lip of calyx with teeth 2–4 mm long, 2 × longer than upper teeth, upper surface of leaves coarsely reticulate	**2**
2	Inflorescence axes, calyces and upper surface of leaves villose when young, pubescent when mature	** var. villosa **
–	Inflorescence axes, calyces and upper surface of leaves sparsely pubescent when young, becoming glabrescent when mature	** var. sinensis **

##### Wisteria sinensis var. sinensis

Taxon classificationPlantaeFabalesFabaceae

≡ Millettiasinensis (Sims) Benth., Pl. Junghuhn. [Miquel] 2: 249 (1852) (as M. “chinensis”)
≡ Rehsoniasinensis (Sims) Stritch, Phytologia 56(3): 183 (1984)
= Wisteriaconsequana Sabine ex Loudon, The Gardener’s Magazine 2: 422 (1827) [Glycinesinensis was cited].
= Wisteriapraecox Hand.-Mazz., Anz. Akad. Wiss. Wien, Math-Naturwiss. Kl. 58: 177 (1921). Type: China, Hunan, “frutex volubilis fl. intense rubroviolaceis (diary no. 2357.88) prope urbem Tschangscha [Changsha] in silvulis apertis reg. subtropicae inter viam militarum Hsingaipu [Xinkaipu] et fluvium [Xiangjian river], 100m.”, legi 10 et 23 March 1918, Handel-Mazzetti 11678, WU0059306 (WU, lecto.!, designated by Compton (2015b)); WU0059305 (WU, isolecto.!); WU0059304 (WU, isolecto.!); WU19240004403 (WU, isolecto.!); Paratype: China, Hunan, “in monte Gu-schan, fl. rosei, prope urbem Tschangscha, 14 April 1918, elev. 150 m. (diary no. 2364), Handel-Mazzetti 11,623, WU0059304 (WU!).

###### Illustrations.

Compton in Curtis’s Bot. Mag. 32 (3–4): 283, Pl. 815, t. 1 & 2 (2015); Wei and Pedley, Fl. China 10: 188, t. 223 [1 - 5] (2010).

###### Distribution.

China (Anhui, Fujian, Guangxi, Hebei, Henan, Hubei, Hunan, Jiangsu, Jiangxi, Shaanxi, Shandong, Shanxi, Yunnan, Zhejiang).

###### Habitat.

Climbing over trees and shrubs and along banks and over thickets 50 to 1800 m.

##### Wisteria sinensis var. villosa

Taxon classificationPlantaeFabalesFabaceae

(Rehder) J.Compton & C.Lane, Wisteria: The complete Guide 282 (2019)

≡ Wisteriavillosa Rehder, Journ. Arnold Arbor. 7: 162 (1926). Type: China, Chili, Temple of Sleeping Buddha, Wo-fu-ssu [Wofosi], Western Hills [Xishan] near Peking, received on 25 July 1923, *Ralph G.Mills*s.n.., A00786449 (A, holo.!)
≡ Rehsoniavillosa (Rehder) Stritch, Phytologia 56(3): 184 (1984)

###### Illustration.

Wei and Pedley, Fl. China 10: 189, t. 223 [7] (2010).

###### Distribution.

China (Hebei, Henan, Shaanxi, Shandong, Shanxi).

###### Habitat.

Climbing over trees and shrubs 100 to 1500 m.

##### Wisteria sinensis var. brevidentata

Taxon classificationPlantaeFabalesFabaceae

(Rehder) J.Compton & C.Lane, Wisteria: The complete Guide 283 (2019)

≡ Wisteriabrevidentata Rehder, Journ. Arnold Arbor. 7: 163 (1926). Type: China, Yunnan, Dongchuan, “Glycine, arbuste grimpant cultivé et subspontané très longs rameaux fl. violettes engrappes, jardins de Tong-tschouan alt, 2500m, Avril”, *E.E.Maire* 458, A00195989 (A, lecto.! designated by Compton (2015b)); P02942331 (P, isolecto.!); K000881058 (K, isolecto.!)
≡ Rehsoniabrevidentata (Rehder) Stritch, Phytologia 56(3): 184 (1984)

###### Distribution.

China (Fujian, Jiangxi, Hunan, Guizhou, Yunnan).

###### Habitat.

Climbing over trees and shrubs 100 to 1500 m.

## Supplementary Material

XML Treatment for Wisterieae

XML Treatment for Adinobotrys

XML Treatment for Adinobotrys atropurpureus

XML Treatment for Adinobotrys vastus

XML Treatment for Sarcodum

XML Treatment for Sarcodum scandens

XML Treatment for Sarcodum bicolor

XML Treatment for Sarcodum solomonensis

XML Treatment for Endosamara

XML Treatment for Endosamara racemosa

XML Treatment for Endosamara racemosa var. racemosa

XML Treatment for Endosamara racemosa var. pallida

XML Treatment for Sigmoidala

XML Treatment for Sigmoidala kityana

XML Treatment for Nanhaia

XML Treatment for Nanhaia speciosa

XML Treatment for Nanhaia fordii

XML Treatment for Wisteriopsis

XML Treatment for Wisteriopsis japonica

XML Treatment for Wisteriopsis japonica var. japonica

XML Treatment for Wisteriopsis japonica var. alborosea

XML Treatment for Wisteriopsis kiangsiensis

XML Treatment for Wisteriopsis reticulata

XML Treatment for Wisteriopsis reticulata var. reticulata

XML Treatment for Wisteriopsis reticulata var. stenophylla

XML Treatment for Wisteriopsis championii

XML Treatment for Wisteriopsis eurybotrya

XML Treatment for Callerya

XML Treatment for Callerya nitida

XML Treatment for Callerya nitida var. nitida

XML Treatment for Callerya nitida var. minor

XML Treatment for Callerya nitida var. hirsutissima

XML Treatment for Callerya dielsiana

XML Treatment for Callerya bonatiana

XML Treatment for Callerya cochinchinensis

XML Treatment for Callerya cinerea

XML Treatment for Serawaia

XML Treatment for Serawaia strobilifera

XML Treatment for Whitfordiodendron

XML Treatment for Whitfordiodendron scandens

XML Treatment for Whitfordiodendron nieuwenhuisii

XML Treatment for Whitfordiodendron erianthum

XML Treatment for Whitfordiodendron sumatranum

XML Treatment for Kanburia

XML Treatment for Kanburia chlorantha

XML Treatment for Kanburia tenasserimensis

XML Treatment for Afgekia

XML Treatment for Afgekia sericea

XML Treatment for Afgekia mahidoliae

XML Treatment for Padbruggea

XML Treatment for Padbruggea dasyphylla

XML Treatment for Padbruggea maingayi

XML Treatment for Padbruggea filipes

XML Treatment for Padbruggea filipes var. filipes

XML Treatment for Padbruggea filipes var. tomentosa

XML Treatment for Austrocallerya

XML Treatment for Austrocallerya australis

XML Treatment for Austrocallerya megasperma

XML Treatment for Austrocallerya pilipes

XML Treatment for Wisteria

XML Treatment for Wisteria frutescens

XML Treatment for Wisteria frutescens subsp. frutescens

XML Treatment for Wisteria frutescens subsp. macrostachya

XML Treatment for Wisteria brachybotrys

XML Treatment for Wisteria floribunda

XML Treatment for Wisteria sinensis

XML Treatment for Wisteria sinensis var. sinensis

XML Treatment for Wisteria sinensis var. villosa

XML Treatment for Wisteria sinensis var. brevidentata

## Appendix 1

**List of Herbarium specimens of newly circumscribed taxa examined for this study. All herbarium specimens seen for species transferred to newly described genera in this paper are documented as well as all vouchers used to illustrate Figure 2, distinctive morphological characters in Tribe Wisterieae.**

***
Austrocallerya australis
***

AUSTRALIA: **New South Wales**: Cooper Creek Rd, north of Mullimbimby 8 November 1967, *A.R.H.Martin* 1392 (K). **Norfolk island**: upper slopes of Mt. Pitt, 26 August 1964 *G.Uhe* 1182 (K); Longridge distr. c. 400 ft., May-June 1937, *Capt. J.D.McCornish* 16 (K); in Hooker Herbarium 1867, *Cunningham* s.n.. s.d.; south slope of Mt. Pitt elev. 600 ft, 16 December 1959, *R.D.Hoogland* 6640, (L1978550). **Queensland**: Moreton, Numinbah forest reserve, Nerang river, opposite Chesters Rd. 28 July 2012, P.I.Forster PIF38831 (US02324718).

PAPUA NEW GUINEA: Nondugl western highlands distr. c. 5000 ft. no date, *J.S.Womersley* NGF4470 (K, L1978542); 5 miles east of Okapa eastern highlands, 5000 ft., 24 September 1964, *Thomas G.Hartley* 13092 (K, L1978540); Tagalinga, Jimmi Valley, western highlands elev. 5100 ft., 16 June 1955, *J.Womersley & A.Millar* NGF7700 (K, L1978543); eastern highlands distr. track for Arau-Obura elev. 1500m. 18 October 1959, *L.J.Brass* 32129 (K, L1978545); 1 mile west of Kopiago airfiled, western highlands distr. elev. 4000 ft., 3 November 1968, *J.S.Womersley, J.Vandenberg & M.Galore* NGF37315 (L1978545); western highlands, Komun-Pin divide, east of Korn, upper Wahgi valley, elev. 1650 m. 10 September 1956, *R.D.Hoogland & R.Pullen* 6176, (L1978547, US02324718); eastern highlands distr. Arau to Obura, elev. 1500 m. 18 October 1959, *L.J.Brass* 32129 (K, L1978544); western highlands near Enjumanda village, middle Tale valley, Wabag distr. elev. 7000 ft., 27 June 1960, *R.D.Hoogland & R.Schodde* 6773 (L1978541); Lower Fly river east bank opposite Sturt Island, October 1936, *L.J.Brass* 8214 (K, L1978546). **Bougainville island**: south slopes of Crown Prince Range elev. 2000 ft. 1936, *A.H.Voyce* D13 (K).

***
Austrocallerya megasperma
***

AUSTRALIA: **New South Wales**: Korumbyn Creek 8 miles southwest of Murwillumbah, 6 September 1972, *R.Coveny & A.N.Rodd* 4516 (K, L1978814); Richmond river, received May 1867, *C.Moore s.n..*, (K000880987); 25 km north of Lismore near Whian Whian, 30 October 1981 *A.Kanis 2113* (CANB301997, L1978815). **Queensland**: Currumbin Creek road 28 September 1965, *J.Gillieatt* 399 (K); Moreton Bay district, s. d. 1859, *W.Hill* s.n.. (K); Moreton Bay, 12 October 1958, *D.Norris* s.n.. (K); Fraser Island, 15–16 October 1930, *C.E.Hubbard* 4422 (K, L1978813); Kunda Park on the Marrochydore Rd, 11 May 1977, *J.A.Elsol* 121 (K); eastern spur of McPherson Range 47 miles south of Ipswich, 5 April 1953, *R.Melville & T.Hunt* 3634 (K); Como State Forest, Kooloolah, Gympie, 13 October 1988, *P.L.Swanborn* QL883, (L1978812); Moreton sports Rd. Bli Bli, 23 July 2012, *G.Leiper*, AQ818814 (US02324711).

***
Austrocallerya pilipes
***

AUSTRALIA: **Queensland**: Cape York, Harvey Creek elev. 30 m. rainforest, 23 October 1989, *B.Gray* 20267 (K, L3884382); Cape York, State Forest Rd 143, Parish of Riflemead, Lerra Logging Area, elev. 500 m. 25 October 1989, *B.Gray* 05144 (K); SFR Riflemead, Lerra LA, elev. 500 m. 3 July 1986, *B.Gray* 04932 (K); SFR 143 Lerra LA elev. 1200 m. 27 October 1988 *B.Gray* 04932 (K); Johnstone river, August 1892, *F.M.Bailey* s.n.., (K000880982); Cook Co., start of Bartle Frere walking track, 5 December 2001, *R.Booth & R.Jensen* 2788, (L3892264, NY03556966); rain forest Mt Lewis road 5 km from main road, 5 November 2002, *B.Gray* 08360, (L3894665); North Queensland, Mossman river gorge, 3 February 1959, *L.J.Brass 2133* (US02324776).

***
Kanburia chlorantha
***

THAILAND: **Tak Prov.**: N. Tak Lansang, Pa Ban Om Yom, elev. 520 m., 9 August 1968, *S. Phengnaren*, (BKF57659, 1978942, L1978943).

***
Kanburia tenasserimensis
***

THAILAND: **Kanburi Prov.**: Ta Salao, Kanburi, 10 July 1930, *Phraisurind Put* 3049; Ta Salao 10 July, 1930, *Put* 3049 (K, L1978938, L0475175).

***
Nanhaia fordii
***

CHINA: **Guangxi Prov.**: Zhuang Autonomous Region, Pingle Co. Guangyun forest farm, Maozi Chong, 9 October 1958, *Yinkun Li* 401972, (IBK00073457); Guangxi, Zhuang Autonomous Region, Guixian Longyang distr. Tanyang township, 29 June 1957, *Chen Zhaozhou* 50790, (IBK00073456).

VIETNAM: Taai Wong Mo Shan, vicinity of Tong Fa, Ha-Coi, Tonkin, 11–23 September 1939, *W.T.Tsang* 29588, (P02753519).

***
Nanhaia speciosa
***

CHINA: **Guandong Prov.**: Thai-Yong, elev. 2000 ft., west of Swatow [Shantou], *Dr. J.M.Dalziel* s.n.., (E00124678); Tai-O [Lantau island], 17 August 1929, *Tsiang Ying* 3105, (E00124674); King P’ing Shan, T’sang Faan, Feng Ch’eng distr. 1–9 September 1936, *W.T.Tsang* 26766 (K); Hong Kong, Sai Tsui, New Territories opposite High Island, 21 October 1969, *Shiu Ying Hu* 8313 (K); Hong Kong, Herb. of U.S. North Pacific Exploration Exped. Rinngold & Rodgers 1853–1856, *C.Wright* 138 (K); Hong Kong, Pine Grove New territories, 21 September 1969, *Shiu Ying Hu* 7924 (K); Hong Kong, Ma On Shan, Sai Kung, 20 August 1973, *Kit Yock Chan* 125, (P02754272); Hong Kong, Pat sin ling N. T. 29 October 1968, *Shiu Ying Hu* 6091 (K). **Hainan Island**: Liamui, elev. 400 m, June-July 1935, *J.Linsley Gressitt* 1194, (E00124679); Lingnan Univ. 17405, Paak Shak shan, Ngau Ku Tsai Lak, 12 June 1928, *Wait-Tak Tsang* 656 (K); Dung-ka elev. 1700 ft. 1932–1933, *N.K.Chun & C.L.Tso* 43467 (K); Ngau Tsai T’ang, Nodoa, Taam-chau distr., 18 July 1927, *W.T.Tsang* 66 (K); Ngou Mou Tai Lik, Pak Shik Ling, Ku Tung village, Ching Mai distr., 27 September 1932, *C.I.Lei* 31 (K).

VIETNAM: Ouonbi, Tonkin, October 1885, *B.Balansa* 1205 (K); environs de porte de Bat Bae, September 1888, *B.Balansa* 2230 (K, P02754259, P02754261); Sai Wong Mo Shan (Sai Vong Mo Leng) Lomg Ngong village, Dam-Ha, 18 July-9 September 1940, *W.T.Tsang* 30375 (K, P02754251, P02754254); Tsai Wong Mo Shan, Tong Fa market, Ha-coi, 11–23 September 1939, *W.T.Tsang* 29510 (K, P02754253): Tsai Wong Mo Shan, Tong Fa market, Ha-coi, 11–23 September 1939, *W.T.Tsang* 29542 (K, P02754252); Tsai Wong Mo Shan,Chuk-phai, Ha-coi, 18 November – 2 December 1936, *W.T.Tsang* 27192 (K, P02754255); Phu Ho, August 1923, *Petelot* 1088 (K, P02754256, P02754257); Kau Nga Shan, Tien-yen, 1–9 January 1937, *W.T.Tsang* 27534 (K, P02754258); Ninh Thai, Muou Lang 14 September 1888, *H.Bon* 3931 (K, P02754260, P02754262, P02754263); Ninh Thai, 21 August 1889, *H.Bon s.n..* (K, P02754264); Guang You, Tonkin, August 1885, *B.Balansa* 1204 (K, P02754265, P02754274); Ouonbi, Tonkin, 13 September 1885, *B.Balansa* 1203 (K, P02754266, P02754267, P02754268).

***
Serawaia strobilifera
***

INDONESIA: [Borneo] **Kalimantan**: east Kutei, Susuk region, elev. 10 m, 26 June 1951, *A.Kostermans* 5442 (K, L1978967); Kalimantan east, Kutei, Sangkulirang [Miang] island elev. 30 m, 24 May 1951, *A.Kostermans* 4898 (K, L1978985); Kalimantan, west Kutei, near Long Liah Leng elev. 250 m, 31 August 1925, *F.H.Endert* 3031 (K, L1978972); west Kutei elev. 130 m, 18 August 1925, *F.H.Endert & L.Iboet* 24 (K); Kalimantan central, Bukit Raya Exped. 21 November 1982, upper Katingan (Mendawai) river area, upper Samba river Tumbang Habangoi to Tumbang Riang, elev. 50 m., *J.P.Mogea & W.J.J.O de Wilde* 3547 (L); west Koetei, elev. 50 m, exped. Midden-oost Borneo, 7 August 1925, *F.H.Endert* 2387 (L1978976, L1978973); Bukit Raya Exped. Tumbang Atey, elev. 100 m, 28 January 1983, *Wiriadinata* 3444 (L0475064, L1978974); West Koetai no. 24, steep river bank, elev. 130 m, 18 August 1925, *F.H.Endert & L.Iboet* 2653 (L0503365); Kalimantan east, Gunung Kongkat to Gunung Kongbotak, elev. 350 m, 27 January 1981, *Masahiro Kato and Harry Wiriadinata* B-5145 (L1978978); east Kutei, G. Tepian Lobang, on Menubar river north-east of Sangkulirang, elev. 200 m, 7 August 1951, *A.Kostermans* 6036 (L1978983).

MALAYSIA: [Borneo] **Sabah**: Ranau distr., Kampung Takutan, Tapi Sungai mekeden, Tengkunanawau, 15 May 1995, *Lomudin Tadong* 308 (K); Tawau, Kawa rd., 30 May 1962, *Jaswir & Aban* SAN 26274 (K, L1978984); Sungai [river] Raoen, 1893–1894, *H.Hallier* 3364 (K, L1978968); Diwata, Kennedy Bay [timber] Co., 9 June 1961, *F.R.Muin Chai* SAN 25083 (K); Segaluid Lokan Forest Reserve, Sandakan, 25 January 1975, *Aban & Leopold* SAN 80986 (K); mile 12 Hap Seng timber complex, Brantian rd., climber 28 ft., 17 March 1979, *Fidilis Krispinus* SAN 89763 (K, L1978970); Kabili Sepilok Forest Reserve, Elopura Forest Distr., climber 25 ft., 4 May 1939, *Enggoh* 10531 (K, L1978969); Lahad Datu, mile 4, Silam, elev. 500 ft., 22 July 1966, *Ahmad Talip* 54923 (L1978975); Sabah? Bukit Batai, Exped, Nieuwenhuis 1896–1897, July 1897, *Jaheri* 374 (K, L0503363); Pinangah forest reserve, Telupid distr. 12 December 1993, *Fidilis Krispinus* SAN 135655 (L1978981).

***
Sigmoidala kityana
***

THAILAND: **Chiangmai Prov.**: elev. c. 300 m, 8 September 1921, *A.F.G.Kerr* 5658 (K, BK258012); Siam, Me-Ban-Prae, 11 October 1929, *C.W.Franck* 599 (L1978811); Loei, Nong Hin, Ban Suan Hom village, Suan Sawan karst limestone, 15 November 2011, *R.P.Clark* 245 with *P.Wilkin, P.Suksathan, K.Keeratikiat, A.Trias-Blasi & Mr Phitak* (K); Lampun, Me Li, elev. 270 m. 13 November 1925, *Winit* 1557 (K, BK258013).

***
Wisteriopsis championii
***

CHINA: **Fujian Prov.**: March 1962, *Cai Jue* s.n.., (NAS00390280); (as Fukien) Foochow, Ja-Miao shan, no date, *H.H.Chung* 2786 (K); (as Fukien), Yuping, Huang-chin shan, elev. 130 m. 17 August 1924, *H.H.Chung* 3008 (K). **Guandong Prov.**: Haifeng County, rare beside road, 20 August 1958, *Wei Zhaofen* 121335 (PE00410165); Hong Kong, Fo-Tan south valley, fls greenish-white, leaves glabrous, stipuleoles setose, 20 June 1970, *Shiu Ying Hu* 10476 (K, PE00410166); Ruyuan Yao Autonomous Co., mountain habitat between Linxia and Baishui villages, 14 August 1935, *W.Y.Chun* [*Chen Huanyu*] 10776 (PE00410167); Ying Tak [Yingde Distr.], Wen Tong Shan [Wentangshan], 6 June 1932, *T.M.Tsui* 412 (PE00410168); Dapu County, Tai Mo Shan in clay by roadside among thickets, 13 July 1932, *W.T.Tsang* 21166 (P02754297, PE00410169); Hong Kong, main island, on cliff beside shore, 27 September 1972, *Shiu Ying Hu* 12214 (K, PE00410170); Hong Kong, Cheung Shu Tan New Territory, flowers white, standard marked with a green spot at base, 19 May 1973, *Kit Yock Chan* 092 (P02753548, PE00410171); Hong Kong, Ma on Shan New Territories 22 September 1968, *Shiu Ying Hu* 5648 (PE00410172); Yingde Co. Wentangshan 6 June 1932, *T.M.Tsui* 412 (NAS00390863); Shixing Co. Yao township, 16 July 1958, *Deng Liang* 6906 (HITBC019545); Ruyuan Yao Autonomous Co. Baixia village among rocks 14 August 1935, *Chen Huanyu* 10776 (SZ00111312); *C.Wright s.n..* for Rinngold-Rodgers exped. 1853–1856 (K); Hong Kong, High Island, New Territories, 11 November 1969, *Shiu Ying Hu* 8626 (K); Hong Kong, June 1874 Herb. Hance 10121 (P02754293); Hong Kong, *Mr [Robert] Fortune* 62. 1845 Collection de la Societe Horticulturale de Londres 1856 (P02754294); Hong Kong, Mt. Gough, 22 May 1895, *E.Bodinier* 1209 (P02753547, P02754295); Hong Kong, *C.Wright* 136 for Rinngold and Rodgers exped 1853–1856 (P02754296).

***
Wisteriopsis eurybotrya
***

CHINA: **Guangxi Prov.**: Long-tcheou [Longzhou] received 14 February 1911, *Dr. Simond s.n.*. (P02753456). **Guizhou Prov.**: (as Kouy-tcheou), Kiang Long, Keou Lau tse, August 1910, *M.Cavalerie s.n.*. (P027534, P02753465).

VIETNAM: Hue and vicinity, Tourane, Annam, below Hue divide, May-July 1927, *J. & M.S.Clemens* 3967 (P02753504, U1262152); in monte Chua Hae, 1 July 1889, *H.Bon* 2962 (P02651680); Tu Phap, August 1887, *B.Balansa* 2240 (P02753455); collines sur la rive gauche de la riviere noir, en face de Phuong Lam, 13 January 1887, *B.Balansa* 2302 (P02753444, P02753445); Kau Nga Shan and vicinity, Tien-yen, 1–9 January 1937, *W.T.Tsang* 27510 (P02753461); Canh Vrap, recd. December 1903, *Dr Spire* 1210 (P02753462); Day Bong Hong, gare de Cau Hai, Hue 14 September 1938, *E.Poilane* 27843 (P02753463); Annam, Col d’Ailao, Quang Tri Prov. 400 m. 1937, *M.Poilane* 26623 (P02753466); route de Phu Quy a Kebon, 4 August 1929, *M.E.Poilane* 16576 (P027534567); Ninh Binh Prov. Cuc Phuong Nat. Park, 26 August 1999, *N.M.Cuong* 463 (L1978805); Yen Lac village Yen Thuy distr. Hoa Binh Prov. 21 August 2000, *N.M.Cuong, D.T.Kien & M.V.Sinh* 1021 (L1978804); Mt. Bani 25 km from Tourane, May-July 1927, *J. & M.S.Clemens* 3742 (U1262153).

LAOS: Expedition du Me-Kong, Paklai a Luang Prabang 1866–1868, *Dr Thorel s.n.*. (P02753450, P02753458); Luang Prabang, 1890, *Mussie s.n*. for L.Pierre (P02753457); Luang Prabang 1866–1868, *Dr Thorel* 9441 (P02753459); Vientiane Prov. Hin Heup distr. Nam Lik river c.5 km west of Khon Phol village elev. 225 m. 22 August 1999, *J.F.Maxwell* 99–145 (L1978803).

***
Wisteriopsis japonica
***

JAPAN: **Honshu**: precise location unknown, 24 Aug. 2003, *M.Furusawa & M. Miyake* FOK-059278 (E00661878); Honshu cult. 12 Sept. 1937, *T.Makino s.n..* (L0282763); Yamamoto in Settsu 9 Sept. 1956, *M.Togasi* TNS [National Museum of Nature and Science] 1497 (K x 2); Hondo, Yamamoto, Kawabe-gun in Settsu, 3 June 1950, *M.Togasi* NSM 1. (L2010848, US02324943); Tarumi-ku, Koobe-shi, settu Prov. Pref. Hyogo. Mt. Mekko-yama, 22 Oct. 1975. Elev. 40–150m., *Miyoshi Furuse* 9745 (K); Kawanishi, Iwakuni city, Shiro-yama Nat. Forest, north bank of Misyo river, *L.A.Charette* 1834 (US02324044); Yamaguchi Pref. Kawanashi, Yokoyama village, Iwakuni city, Shiro-yama Nat. forest, north bank of Misyo river *L.A.Charette* 1557 (US02324045). **Kyushu**: US North Pacific Expedition 1853–1856, *C.Wright s.n..* (NY00016341); Nagasaki, 1863 ex herb. Horti bot. Petropolitani, *Maximowicz s.n..* Iter secundum (P02942301); Nagasaki, 1863 ex herb. Horti bot. Petropolitani, *Maximowicz s.n..* Iter secundum, (P02942305); *Siebold s.n..* Herb. Lugd.-Batav. 908.120-13 s.d., *Siebold s.n..* (L0176060); Herb. Lugd.-Batav. 908.120-13 s.d., *Siebold s.n..* (L0176061); Nagasaki, 1862, *R.Oldham* 386 (L2010846); ex Herbario Lugduno-Batavo s.d., *H.Bürger s.n.*. (M0153889); ex Herbario Lugduno-Batavo s.d., *H.Bürger s.n.*. (M0153890); ex Herbario Lugduno-Batavo s.d., *H.Bürger s.n.*. (M0153891); Nagasaki. 1826, *R.Oldham* 386 (K x 2); Ssama-yama, Shinano, July 1883, Science Coll. Imp. Univ. [TI] collector unknown, s.n.. (K); Moji [Kitakyushu] 19 Sept. 1906. Bushy hillside, *S.T.Dunn* 8526 (K); Nanokawa-tosa 22 July 1892, *K.Watanabe s.n..* (K); Nagasaki, 1892, *Maximowicz s.n..* (K); Nagasaki, July 1862, *R.Oldham* 801 (K); Miyazaki Pref., Koyu County. Uwae township, *N.Eri* 100555, s.d. (US02324041). **Shikoku**: Between Kaminokae and Umagura, Nakatosa town, Kochi Pref. 15 August 2003, *Taku Miyazaki* 308058 (L3892474); collector locality and date unknown “kofusi” Japan (L2010847); collector locality and date unknown “kofusi” Japan (L2010847); Shikoku without precise locality, 20 Nov. 1914, *E.H.Wilson* Arnold Arb. 7796 (K); *C.Wright* Rinngold & Rodgers Exped. 1853–1856 without locality (K); Minoo, Higashidani Pref. Oosaka, 20 Aug. 1961, *M.Togashi* 7888 (K); Mie, Shima, Toba, collector and date unknown, (US02324042).

***
Wisteriopsis kiangsiensis
***

CHINA: **Anhui Prov.**: Lin yungchi, southern Anhwei elev. 250 m., 30 June 1925, climber 7 m. high, *Qin Renchang* [*Ren-Chang Ching*] 2881 ex Herbarium of Biological Laboratory Nanking China 5130 (PE00417692, para.!, K, isopara.!); Taiping Co. Zhangjiabang, Jiao village, by roadside 300 m. 18 June 1959, collector unknown Herb. number 0044 (NAS00390600); Taiping Co. Qidu commune, Longguang Brigade, 19 June 1959, collector unknown 0814 (NAS00390591); Xiuning Co. among hills elev. 300 m. 17 June 1959 collector unknown 1973 (NAS00390609); Meiguang Co. Houtian Brigade, elev. 220 m. 1 July 1959, collector unknown 7032 (NAS00390597); Xiuning Co. near Wucheng, hillside forest, 27 June 1959, *Danrenhua et al.* 2627 (NAS00390608); Qimen Co. 11 October 1957, *Deng Shubin et al.* 4818 (NAS00390607); Anhwei, *A.Rehder* 3091, s.d. (PE00320076); Xiuning Co. five cities, 17 June 1959, collector unknown (PE00410855); Huangshan City, 18 June 1959, elev. 300 m. *Chen Jiadong s.n..* (PE00410856); Huangshan city, Taiping, 1959 collector unknown (PE00410857); Near Fenglong Temple elev. 120 m. *Zhou, Xu, Pan & Cheng* 304 (PE00410858); Ye-hsien, southern Anhwei, 800ft climber to 15 ft. common, July 27 1925, *Ren-Chang Ching* Arnold Arb. 3091 (K); West Siunin, 1 August 1925, *R.C.Ching* 8835, (US). **Guizhou Prov.**: Jinping Co. riverside, 25 May 1964, Wang Yumin 0014 [as *Millettiachampionii*] (GFS0002828); Jinping Co. 20 September 1964, Wang Yumin 1154 [as *Millettiachampionii*] (GFS0002830); Jinping Co. 17 September 1964, Wang Yumin 0653 [as *Millettiachampionii*] (GFS0002829). **Hunan Prov.**: Linxiang Co. mountain habitat, 27 June 1974, collector unknown (PE00410872). **Jiangxi Prov.**: Wugongshan elev. 380 m., 21 September 1983, Zhao Qizheng & Gao Xianming 1235 [as *Millettiachampionii*], (CSFI015674); Zixi Co. elev. 210 m. 3 November 2001, Shi Jianmin 011460 [as *Millettiachampionii*] (JXAU0007470); Guangchang elev. 490 m. 4 April 1965, Jiangxi Univ. 650552 collector unknown [as *Millettiachampionii*], (WUK0255610); Pengze, Yaofang Forest Farm, Taohongling Nature Reserve 18 July 2015, elev. 200 m., *Li Enxiang, Liu Yizhen & Cai Qiying* 15070834, (JJF00019576); Xihong Reservoir, Taohongling 28 July 2006, *Tan Chengming, Zhu Huanbing & Wu Haiching* 06804, (JJF00019575); Taihe Co. by river, elev. 170 m. 23 September 1973, *Shi Xinghua* 730132 [as *Millettiachampionii*] (JXAU0007475); Jinggangshan elev. 420 m. 28 July 1988, *Sun Yegen* 00495 [as *Millettiachampionii*] (JXAU0007474); Zixi Co. elev. 390 m. 30 October 2001, *Liu Renlin et al.* 011765 [as *Millettiachampionii*] (JXAU0007471); Taihe Co. by riverside elev. 170 m. 23 September 1973, *Shi Xinghua* 730132 [as *Millettiachampionii*] (JXAU0007476); Taohongling 18 July 2015 elev. 200–500 m. *Li Enxiang et al.* (JXU0007874, L2015070221); Xiushui Co. edge of bamboo forest elev. 500 m. 5 November 1995, *Ye Cong* [*Ye Cun-su*] 943 [as *Millettiachampionii*] (NAS00399073); Wuning County, Yishan, 31 July 1936, *H.K. Teng* 352 [as *Millettiachampionii*] (PE00410164); Longnan County, Cheng longxiang jiao keng, along streamside *Moximu* 21026, s.d., (PE00320077); Poyang Co. June 1959, *Li Qihe & Chen Ze* 1014 (PE00410868); Jiangxi Prov. Dongxiang Co. hillside, 30 June 1959, *Li Qihe & Chen Ze* 1434 (PE00410870); Wuning Co. 28 June 1947, *Xiong Huiguo* 05013 (PE00410869): Changkeng, 18 June 1936, *H.K.Teng* 229 (PE00410864); Changkeng, 18 June 1936, *H.K.Teng* 233 (PE00410863); Wuning Co. Yishan, 31 July 1936, *H.K.Teng* 353 (PE00410862); Wuning Co. Yishan, 8 August 1936 *H.K.Teng* 362 (PE00410866). **Jiangsu Prov.**: Qutang township by roadside elev. 142 m. 27 September 2015, *Chen Gongxi, Zhang Daigui et al.* LXP-06-5873, (JIU08535); Lianyungang, Huang wofujin,14 Sept. 1952, *Chen Changdu, Wang Jinting & Dong Huimin* 21023, (PE01566389). **Zhejiang Prov.**: Zhoushan, Jintang Island, Xishangang, 27 June 2012, Bi Yuke BYK1742, (CSH0055973); Jianye, Meishi Dashiping, 6 September 1979, *Honglin s.n..*, (HZ020355); Yaofang Forest Farm, Taohongling Nature Reserve 18 July 2015, elev. 200 m., *Li Enxiang, Liu Yizhen & Cai Qiying* 15070834 (JXU0007861); Yanxia village forest, 11 November 2015, *Tan Weizheng, Ding Qiaoling, Zhao Wanyi, Liu Zhongcheng, Zhang Zhong & Ye Xi* LXP-13-08399 (SYS00182930); near Hangzhou, 21 July 1980, *Hang Zhibiao* 1583, (NAS00396669); Yu-tsien Hsien, open thickets, 27 June 1927, *Y.L.Keng* 548 (PE00410859); Chunan Co. Tangcun farmhouse, Shangbao village 29 August 1980 collector unknown (PE00410860); Near Tatzechiao, 7 July 1915, *F.N.Meyer* 1517, (US).

***
Wisteriopsis reticulata
***

CHINA: **Anhui Prov.**: (as Anhwei) Ye-hsien elev. 50 ft. 3 July 1929, *Ren-Chang Ching* 3080 (K). **Fujian Prov.**: (as Fukien), 8 April 1923, *H.H.Chung* 1265 (K); (as Fukien), Minhow Hsien, Hwai-On, 17 July 1923, *H.H.Chung* 2464 (K); (as Fukien), Minhow Hsien, Pehling, 13 August 1923, *H.H.Chung* 2063 (K); (as Fukien), Kuliang Hills near Foochow elev. 1000–3000 ft, July-August 1919, *J.B.Norton* 1307 (K). **Guandong Prov.**: Hong Kong, Lantau Island, August 1886, collector unknown (K); Hong Kong, prope Tai Tam Tuk, June 1859, * Hance* 1489 (K); (as Kwangtung), Ying-tak Distr. 1 August 1921, *Y.K.Wang* 2914 (K); Guangdong, without locality, August 1887, flowers rose, *C.Ford* 36 (K); (as Canton) *Theophilus Sampson* s.n.. 2 June 1885 (K); (as Kwangtung) August 1887, *C.Ford* 43 (K); (as Canton), 19 June 1886, *Theophilus Sampson* s.n.. (K); (as Kwangtung) Wan Tong shan, Ying Tak Distr. 6–24 June 1932, *T.H.Tsui* 412 (K); (as Kwangtung), Lokcheng, 20 June 1929, *C.L.Tso* 21163 (K). **Hainan Island**: Chiu-shan, Ling-shui Co. Fan Haan Ts’uen, 4–20 May 1932, *F.A.McClure* 20117 (K); Santsigian (Lai), Chiu-san Tsuen (Ngai Distr.) 7–25 August 1932, *S.K.Lau* 369 (K); Hung-Mo shan, Sai In Low King Tang, 3 July 1929, *Tsang & Fung* 422, Lingnan Univ. 17956 (K); Hainan, without precise locality 21 July 1938, *C.Wang* 33196 (K); Lin-Fa shan 8 August 1927, *Tsang Wai-Tak* 398, Lingnan Univ. 15397 (K); Lo King T’ang, Sin Wah (Twan Chan distr.) 18 May 1928, *Tsang, Wai-Tak* 354 Lingnan Univ. 17103 (K); Hainan, without precise locality, November 1889, *A.Henry* 8392 (K); Hainan, south of Fan Ta 19 April 1922, *F.A.McClure* 9168 (K). **Hubei Prov.**: Patung, October 1901, *E.H.Wilson* 1017 (K); Ichang, February 1887. *A.Henry* 2280 (K); Ichang, *A.Henry* 631 rec’d March 1886 (K); Western Hupeh, July 1907, *E.H.Wilson* 3282 (K);, Ichang, February 1887, *A.Henry* 1554A (K). **Jiangsu Prov.**: (as Kiangsu) summer 1926, *C.L.Tso* 1697 (K). **Jiangxi Prov.**: (as Kiangsi), Kinkiang, elev. 300 ft. 27 July 1909, *E.H.Wilson* 1648 (K); (as Kiangsi) Si Feng Sze, 12 August 1922, *A.N.Steward* 2648 (K). **Taiwan**: without location “Formosa” 1864 *Richard Oldham* 158 (K); (as Formosa) Kewkiang, Farinhoe, *Dr Shrope* s.n.. comm. April 1875, Hance Herb. 1489 (K); Takeo-san (Apeo hills). 1912. *W.R.Price* 596 (K); Bankinsing, *A.Henry* 894 rec’d 1895 (K);), Kasenko to Santo, Kasenko Prov. 24 November 1918, *E.H.Wilson* 11099 (K). **Zhejiang Prov.**: Ningbo (as Ningpo) *H.B.Forbes* 2/76 coll. 1874 (K); (as Chekiang) Lung Cheung Hsien, elev. 500 ft. 24 September 1920, *H.H.Hu* 439 (K); south-west Chekiang, 18 August 1924, *R.C.Ching* 2432 for A.Rehder (K); Fatze-chiao, 7 July 1915, *F.N.Meyer* 1516 (K); (as Chekiang) Shihpu, 11 August 1927, *C.Y.Chiao* 14114 (K); Ningbo, August 1887 *E[rnst] Faber* 329 (K); (as Chekiang), without locality, *H.H.Hu* 1920 (K).

VIETNAM: Tonkin, Kau-Nga-shan and vicinity, Tien-yen, 23 September to 7 October 1940, *W.T.Tsang* 30541 (K).

**Vouchers used for Figure 2. Distinctive morphological characters in Tribe Wisterieae**

*Endosamararacemosa* Thailand, Prae, elev. 600–1000 ft. April 1910, *Luang Vanpruk* 188 (K); *Padbruggeadasyphylla* Myanmar, Perak, *Scortechini* 429 (K); Malaysia [Borneo], Sabah, Tenom, Palang-Palang hills, 4 January 1991, *Lamb* 395/91 (K); *Austrocalleryaaustralis* Papua New Guinea, eastern highlands, distr. track for Arau-Obura elev. 1500m. 18 October 1959, *L.J.Brass* 32129, (K); *Austrocalleryapilipes* Australia, Queensland, SFR 143, Parish of Riflemead, Lerra, LA, 3 July 1986, *B.Gray* 04319 (K); *Padbruggeafilipes* Myanmar, Sone Lone Taung, 25 February 1927, *Maung Po Khant* 15326 (K); *Afgekiasericea* Thailand, Bankhen, Bangkok 9 September 1968, *C.Chermserivithana* 996 (K); Thailand, May 1935, *Mrs Collins* 104/9 for A.F.G.Kerr (K); *Calleryanitida* China, Hong Kong, 23 September 1885, *Theophilus Sampson s.n..* (K); *Calleryacinerea* Tibet, 1917–1919, *George Forrest* 19279 (K); *Whitfordiodendronnieuwenhuisii* Indonesia [Borneo], Kalimantan, 5 km west of Batu, Badingding, 19 December 1982, *J.P.Mogea* 4182 (K); *Whitfordiodendronerianthum* Thailand, Songkhla Prov., image from photograph *Y.Sirichamorn s.n.*.; *Wisteriopsiseurybotrya* Vietnam, Tourane, May-July 1927, *J.& M.S.Clemens* 3637 (K); *Wisteriopsischampionii* China, Hong Kong, Fo-tan south valley, 20 June 1970, *Shiu Ying Hu* 10476 (K).

## Supplementary materials

Supplementary material 1 Figures S1–S6 Data type: molecular data Explanation note: **Fig. S1.** Combined ML Bootstrap tree of Tribe Wisterieae and outgroups. **Fig. S2.** Combined Bayesian Inference (BPP) tree of tribe Wisterieae and outgroups. **Fig. S3.** Plastid ML Bootstrap tree of Tribe Wisterieae and outgroups. **Fig. S4.** Plastid Bayesian Inference (BPP) tree of Tribe Wisterieae and outgroups. **Fig. S5.** ITS ML Bootstrap tree of Tribe Wisterieae and outgroups. **Fig. S6.** ITS Bayesian Inference (BPP) tree of Tribe Wisterieae and outgroups. This dataset is made available under the Open Database License (http://opendatacommons.org/licenses/odbl/1.0/). The Open Database License (ODbL) is a license agreement intended to allow users to freely share, modify, and use this Dataset while maintaining this same freedom for others, provided that the original source and author(s) are credited.James A. Compton, Brian D. Schrire, Kálmán Könyves, Félix Forest, Panagiota Malakasi, Sawai Mattapha, Yotsawate Sirichamorn
